# Profiling Blood Serum Extracellular Vesicles in Plaque Psoriasis and Psoriatic Arthritis Patients Reveals Potential Disease Biomarkers

**DOI:** 10.3390/ijms23074005

**Published:** 2022-04-04

**Authors:** Freddy Lättekivi, Irina Guljavina, Getnet Midekessa, Janeli Viil, Paul R. Heath, Rikke Bæk, Malene Møller Jørgensen, Aneta Andronowska, Kulli Kingo, Alireza Fazeli

**Affiliations:** 1 Department of Pathophysiology, Institute of Biomedicine and Translational Medicine, University of Tartu, Ravila St. 14b, 50411 Tartu, Estonia; freddy.lattekivi@ut.ee (F.L.); irina.guljavina@ut.ee (I.G.); getnet.balcha@ut.ee (G.M.); 2 Institute of Veterinary Medicine and Animal Sciences, Estonian University of Life Sciences, Kreutzwaldi 62, 51006 Tartu, Estonia; 3 Department of Pharmacology, Institute of Biomedicine and Translational Medicine, University of Tartu, Ravila St. 14b, 50411 Tartu, Estonia; janeli.viil@ut.ee; 4 Sheffield Institute of Translational Neuroscience, University of Sheffield, Sheffield S10 2HQ, UK; p.heath@sheffield.ac.uk; 5 Department of Clinical Immunology, Aalborg University Hospital, Urbansgade 32-36, 9000 Aalborg, Denmark; rikke.baek@rn.dk (R.B.); maljoe@rn.dk (M.M.J.); 6 Department of Clinical Medicine, Aalborg University, Søndre Skovvej 15, 9220 Aalborg, Denmark; 7 Department of Hormonal Action Mechanisms, Institute of Animal Reproduction and Food Research, Polish Academy of Sciences, Tuwima St. 10, 10-748 Olsztyn, Poland; a.andronowska@pan.olsztyn.pl; 8 Clinic of Dermatology, Institute of Clinical Medicine, University of Tartu, Raja 31, 50417 Tartu, Estonia; kylli.kingo@ut.ee; 9 Clinic of Dermatology, Tartu University Hospital, Raja 31, 50417 Tartu, Estonia; 10 Academic Unit of Reproductive and Developmental Medicine, Department of Oncology and Metabolism, Medical School, University of Sheffield, Sheffield S10 2SF, UK

**Keywords:** psoriatic arthritis, circulating EVs, miRNA, biomarker, surface proteome

## Abstract

Psoriasis vulgaris (PsV) and psoriatic arthritis (PsA) are inflammatory diseases with unresolved pathophysiological aspects. Extracellular vesicles (EVs) play an important role in intercellular communication. We compared the miRNA contents and surface proteome of the EVs in the blood serum of PsV and PsA patients to healthy controls. Size-exclusion chromatography was used to isolate EVs from the blood serum of 12 PsV patients, 12 PsA patients and 12 healthy control subjects. EV samples were characterized and RNA sequencing was used to identify differentially enriched EV-bound miRNAs. We found 212 differentially enriched EV-bound miRNAs present in both PsV and PsA groups—a total of 13 miRNAs at FDR ≤ 0.05. The predicted target genes of these miRNAs were significantly related to lesser known but potentially disease-relevant pathways. The EV array revealed that PsV patient EV samples were significantly enriched with CD9 EV-marker compared to controls. Analysis of EV-bound miRNAs suggests that signaling via EVs in the blood serum could play a role in the pathophysiological processes of PsV and PsA. EVs may be able to fill the void in clinically applicable diagnostic and prognostic biomarkers for PsV and PsA.

## 1. Introduction

Psoriatic arthritis (PsA) is a chronic, immune-mediated inflammatory disease of the joints with diverse clinical effects that can affect the axial and peripheral joints, as well as accompanying symptoms of enthesitis and dactylitis [1]. It is a progressive disease with destructive effects on the joints, greatly affecting the quality of life of the patients [2]. During its early stages, the disease can develop and progress without any noticeable symptoms, reducing the chances of early diagnosis. Although PsA is a well-established disease, only 60–80% of patients present signs of psoriatic skin lesions or nail psoriasis. These clinical symptoms can greatly help in diagnosing PsA, and their absence compounds the problem [3]. However, early diagnosis of PsA is crucial to managing the disease, as even a 6-month delay adversely affects the patient’s outcome [4]. That is why PsA patients would greatly benefit from the availability of early biomarker-enabled diagnosis.

As in most cases manifestations of psoriasis vulgaris (PsV) precede PsA, the involvement of joints is often considered a comorbidity of skin psoriasis itself. Up to 30% of patients with PsV develop PsA during the course of the disease [5]. Psoriasis is a relatively common inflammatory skin disease with a prevalence of 2–3% [6]. Various treatments have been proposed in the management of psoriasis and its arthropathic component, including immunosuppressants such as methotrexate or colchicine as well as new drugs [7,8]. Different subtypes of PsV can also present a wide range of clinical phenotypes, with plaque psoriasis being the most common [9]. With no viable means of early detection, psoriasis patients can find themselves in the distressful state of not knowing if and when they would develop PsA. The patients would also benefit from diagnostic clarity enabling timely recognition of the different subtypes PsA can evolve into. An important challenge in the field is the lack of effective and clinically relevant biomarkers for detecting PsA early or monitoring disease progression and treatment outcomes [10,11].

For early diagnosis and preventive screening, non-invasive means of obtaining biomarker measurements are preferred in the clinical setting [12]. While the core pathophysiological processes of PsV and PsA occur in the skin or joints of patients, respectively, the systemic nature of these diseases has revealed several potential biomarker candidates in blood serum and plasma [13]. Conventional circulatory markers of inflammation such as CRP and P-selectin have given mixed results in patients with PsA and patients with moderate to severe forms of PsV [14,15,16,17]. Yet cytokines associated with the central IL-17/23 axis in psoriasis have been elevated in the blood of PsV and PsA patients [18,19,20]. A subset of promising biomarkers has also been identified in the form of miRNAs [21,22]. Extracellular vesicles (EVs) are perhaps the most important representative of circulatory biomarkers discovered in recent years, yet they have remained relatively understudied in the context of PsA and PsV [23,24].

EVs carry signals mediated by proteins, lipids and nucleic acids, and thus play a pivotal role in intercellular communication [25]. Consequently, they have proven to be valuable sources for identifying biomarkers for a number of diseases [26,27,28,29,30]. More notably, recent in vitro studies have identified EVs as an important player in the pathophysiological processes in PsA and PsV. It has been reported that keratinocyte exosomes are implicated in the activation of neutrophils and intensifying inflammation [31]. In addition, Mangino and colleagues have reported that L17A-treated keratinocytes secrete fewer EVs; however, PsV-related mRNAs (DEFB4, CXCL-family ligands) are abundant in these EVs, providing further evidence for EVs mediating pathological signals in PsV [32]. In a study by Marton et al., it was shown that the circulatory EVs from PsA patients are unable to suppress osteoclast differentiation in vitro, compared to those extracted from the blood of healthy individuals and rheumatoid arthritis patients [33]. Therefore, it is reasonable to suggest that disease-specific EV profiles are present in the blood serum of PsA and PsV patients, as in the aforementioned diseases.

According to the current understanding of EV-mediated intercellular communication, it is postulated that the cargo carried by EVs is responsible for the greater part of communication moderated through the cellular release of EVs [24]. There is growing evidence showing that circulating EV-bound miRNAs represent a distinct subset of the total population of extracellular miRNAs in the blood [34,35], suggesting EV-bound miRNAs could be more specific biomarkers in comparison to free-floating miRNA [36]. Therefore, in this study, we focused on characterizing the small RNA contents of the EVs in search of disease-related miRNAs in the blood serum of PsA and PsV patients. In addition, we screened the physical properties and surface proteome profiles of EVs for possible novel biomarker candidates.

As a result, we identified several differentially enriched EV-bound miRNAs in the PsA and PsV groups with previous associations with inflammatory skin disease and joint inflammation along with additional pathway-level insights. We also found the EV surface proteome to be of promising value for uncovering future biomarker candidates. As EV research is expanding rapidly, the development of a wide range of new methods and approaches in EV characterization is advancing the field and shortening the interval of the initial findings from biomarker screenings being translated to the clinical setting to aid patients sooner rather than later.

## 2. Results

### 2.1. Purification of Extracellular Vesicles

**Figure 1:**
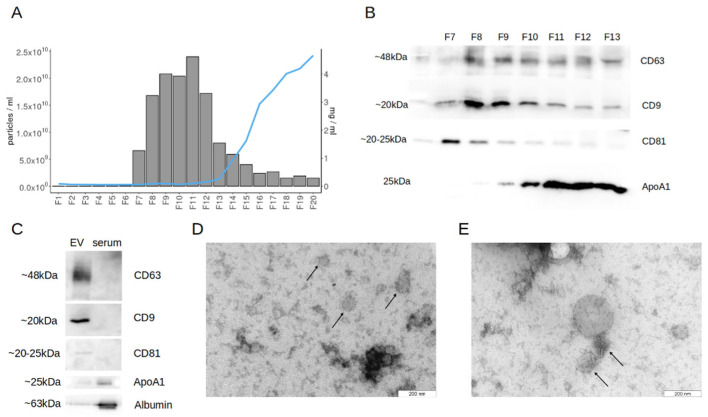
Purification and confirmation of blood serum EVs. (**A**) Elution profile of differentially centrifuged and qEV original 70 nm size-exclusion chromatography (SEC) purification of blood serum EVs in across 500 μL fractions. Consecutively collected fractions are presented on the *x*-axis, while bars represent the nanoparticle concentration (particles/mL) per fraction, and the blue line marks protein concentration (mg/mL) across fractions. (**B**) Western blotting analysis for relative abundance of EV-specific surface markers (CD63, CD9, and CD81) and ApoA1 as the major component of HDL particles in the nanoparticle-rich SEC fractions. Equal volumes of concentrated EV samples were used as the input in case of the tetraspanins, while the input was diluted 10-fold for ApoA1. (**C**) Comparative Western blotting of EV-specific surface markers and ApoA1 in the purified EV samples in contrast to unprocessed blood serum with total protein concentration adjusted to be equal for both samples for each target protein. (**D**,**E**) Transmission electron microscopy imaging of purified EV samples. Black arrows highlight spherical particles with size and morphological characteristics inherent to EVs.

We first evaluated the elution profile of blood serum samples from the qEVoriginal/70 nm SEC columns in order to determine the optimal fractions to use in downstream analysis in terms of EV yield and purity. Using SEC-based EV purification, we observed nanoparticle-enriched fractions to follow the void volume of the column with the total protein concentration increasing after the nanoparticle peak (Figure 1A). The co-isolation of HDL particles alongside EVs from blood serum or plasma is a known issue [37,38], which is why we used Western blotting to characterize the SEC fractions for known EV markers (CD63, CD9, and CD81) and apolipoprotein A1 (ApoA1). It was evident that HDL particles were abundantly present in the nanoparticle-enriched fractions alongside EVs (Figure 1B). We observed that the chemiluminescence of EV markers was relatively stronger in the fractions eluting first (F7–11), while the opposite was evident in case of ApoA1. For all subsequent EV purifications and experiments, we considered the pooled fractions of 7 to 10 to be EV samples. This resulted in an enrichment of EV markers, while the presence of known contaminants such as ApoA1 and albumin was diminished compared to unpurified serum. (Figure 1C). Furthermore, spherical particles with EV-like morphology were evident in the TEM images of EV samples (Figure 1D,E).

### 2.2. Size and ZP Profile of Purified EV Samples

Physical properties of nanoparticles in the EV samples were characterized using a ZetaView^®^ NTA instrument. We found the total concentration of nanoparticles (NPs) in the unconcentrated EV samples to be highly variable across patients, ranging from 2.7 *×* 10^9^ to 2.5 × 10^11^ particles per milliliter (Figure S1A). No differences were observed in the size distribution of the EV samples between patient groups (Figure S1B). Most of the samples were characterized by a distinct peak of NPs in the 80–120 nm size range, whereas a wider distribution range of NP sizes was observed for some samples (Figure S1C). Similarly, no significant differences in the ZP values of EV samples were observed between the patient groups (Figure S1D,E).

### 2.3. EV Array Phenotyping

**Figure 2:**
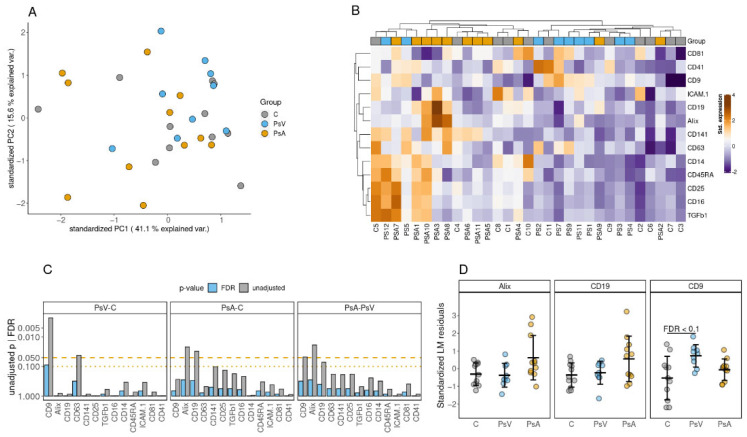
EV Array phenotyping for blood serum EVs. (**A**) The first two principal components calculated from 13 markers considered to be uniformly detected on the surface EVs in at least one of the patient groups. (**B**) Heatmap and hierarchical clustering (Euclidean distance) of linear model residuals resulting from models with patient age and storage time of serum samples as predictors and abundance of the surface marker as the response variable. (**C**) Statistical significances of the observed differences in the abundance of individual EV surface markers between the patient groups. The effect of the patient group on the abundance of each individual surface marker was evaluated by fitting a linear model adjusted by the storage time of serum samples. Both unadjusted *p*-values and *p*-values adjusted to multiple testing with the Benjamini–Hochberg procedure are presented. (**D**) Top three markers resulting from the statistical analysis. The abundance of individual markers are presented as standardized (z-score) linear model residuals after adjusting for patient age and storage time of serum samples.

A total of 30 protein markers were screened using the EV array platform. Thirteen of the 30 markers were considered detectable and subjected to downstream analysis based on the criteria that at least for 6 (≥50%) patients in one of the groups had measured intensity values higher than background signal level. Based on overall profile of the 13 markers, 6 samples were considered outliers and omitted from further analysis (Figure S2). PCA of all of the 13 protein markers revealed the patient samples to be generally heterogeneous, irrespective of the patient groups (Figure 2A). We observed modest correlation between the abundance of some of the markers and the storage time of serum samples (Figure S3A). Therefore, the estimated effect of storage time was deducted by obtaining the residuals resulting from modeling the relationship between the protein markers and storage time. Hierarchical clustering of these residuals resulted in a visible segregation of PsV and PsA samples between the main two clusters, with the control samples (C) samples uniformly scattered between the two (Figure 2B).

Similarly, the storage time was adjusted in the linear regression models used to infer the statistical significance of the differences between the patient groups for individual protein markers. After adjusting for multiple testing (Benjamini–Hochberg procedure), none of the comparisons remained significant at FDR ≤ 0.05 (Figure 2C). However, the differences between the PsV and C groups in the abundance of CD9 on the surface of EVs could be considered statistically significant at FDR ≤ 0.1 (Figure 2D).

### 2.4. Small RNA Profile of EVs

The yield of small RNA extracted from the concentrated EV samples was considerably variable across samples with an average of 5.8 ng (0.4 ng/µL). Interestingly, the RNA yield was not correlated with the concentration of nanoparticles in the EV samples (Figure S3B).

Sequencing yielded 8.7 ± 1.5 million reads (mean ± SD) per sample. Following the trimming of low-quality bases, removal of adaptor sequences, and read length filtering, 44.2 ± 16.8% of the reads remained in the analysis and were aligned to the GRCh38 human reference genome. We observed that the alignment rate among full length reads (50 bp) was extremely low (0.4%) and for the remainder of the analysis, these reads were omitted (Figure S4A). For the remainder of the reads (3.4 ± 1.9 million per sample), the alignment rate was 50.3 ± 9.7%. Sample PS11 was excluded from the analysis due to only 50,000 reads remaining after the removal of full-length reads. The largest proportion of alignments (45.1%) remained unannotated, followed by 37.0% of reads aligning to long non-coding RNA sequences. The estimated proportion of mature miRNA-derived reads was 7.3% (Figure S4B). No distinctive patterns were observed in the overall enrichment of different RNA elements between the patient groups (Figure S4C,D).

### 2.5. Differentially Enriched miRNAs

**Figure 3:**
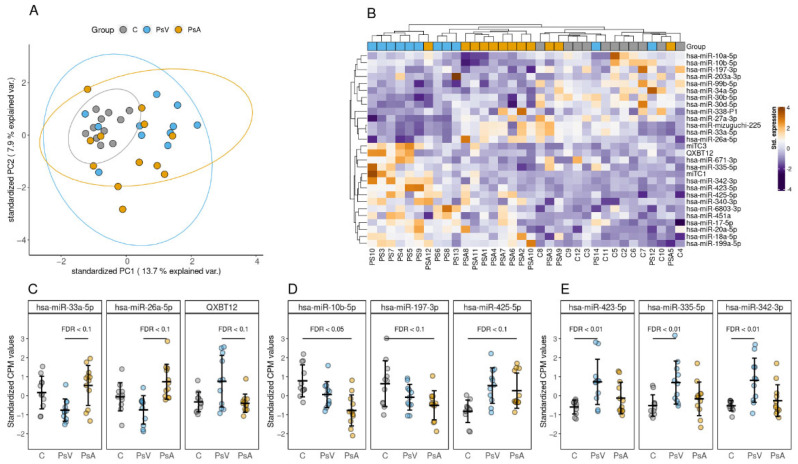
miRNA profile for blood serum EV samples. (**A**) The first two principal components calculated based on 212 miRNAs considered to be uniformly detected in at least one of the patient groups. Ellipses represent 95% confidence intervals. (**B**) Heatmap and hierarchical clustering (Manhattan distance) of miRNAs with an FDR value ≤ 0.1 resulting from any of the pairwise comparisons between the patient groups. (**C**) Top three miRNAs differentially enriched in EV samples isolated from the blood serum of noninflammatory control patients compared to patients with PsV and psoriatic arthritis (PsA). The miRNA abundance is presented as standardized (z-score) counts per million (CPM) values. (**D**) Top three miRNAs differentially enriched EV samples isolated from the blood serum of PSA patients and noninflammatory controls (**C**). (**E**) Top three miRNAs differentially enriched EV samples isolated from the blood serum of PsV patients and noninflammatory controls (**C**).

In total, 212 miRNAs were considered sufficiently detected and subjected to differential enrichment analysis. The enrichment profile of these 211 miRNAs was notably different between the groups, with C group samples displaying lower variance and clustering more tightly, whereas PsV and PsA samples were more dispersed according to the first two components of PCA (Figure 3A).

Comparing the PsA group with PsV group did not result in any miRNAs differentially enriched at FDR ≤ 0.05, but 8 could be considered differentially enriched at FDR ≤ 0.1. The three topmost miRNAs among these results were QXBT12, hsa-miR-33a-5p, and hsa-miR-26a-5p (Figure 3B). The PsA to C group comparison yielded 1 differentially enriched miRNA at FDR ≤ 0.05—hsa-miR-10b-5p—that was observed to be less abundant in the EVs of PsA group patients (Figure 3C). An additional 6 miRNAs could be considered differentially enriched at FDR ≤ 0.1. The highest number of statistically significant results were observed in the comparison between PsV and C groups, where 12 miRNAs were differentially enriched at FDR ≤ 0.05 with an additional 6 at FDR ≤ 0.1. The three topmost miRNAs resulting from the PsV to C comparison were hsa-miR-423-5p, hsa-miR-335-5p, and hsa-miR-342-3p (Figure 3D). Hierarchical clustering based on the FDR ≤ 0.1 provides decent distinction between the conditions and results in the three groups being clustered in individual subclusters with some exceptions (Figure 3E). All miRNAs found to be differentially enriched are listed in Table 1. Results of differential enrichment testing for all of the 211 miRNAs can be found in Supplementary Tables S3–S5.

Enrichment analysis of associated Reactome-annotated pathways was conducted on the miRDB-derived predicted targets (score ≥ 90) of the differentially enriched miRNAs from the PsA to PsV groups. This resulted in the identification of differentially enriched miRNAs from the PsA to PsV group (487 genes), which gave rise to 3 statistically significant processes at FDR ≤ 0.05 (Table S6): transcriptional regulation by MECP2, circadian rhythm, and intercellular signaling by second messengers. Analysis of the predicted targets of the significant miRNAs resulting from the PsA to C comparison (346 genes) resulted in a single statistically significant process at FDR ≤ 0.05 (Table S7), the dopamine neurotransmitter release cycle. The pathway enrichment analysis of the target genes (419 genes) of miRNAs was found to be statistically significant when comparing PsV to C groups. This comparison produced 12 statistically significant processes at FDR ≤ 0.05 (Table S8), along with class I MHC mediated antigen presentation via processing by ubiquitination and proteasome degradation. The miRDB-derived predicted targets (score ≥ 90) of each miRNA used for the pathway-level enrichment analysis can be found in Supplementary Tables S9–S11.

## 3. Discussion

In this study, we characterized EVs in the blood serum of chronic plaque psoriasis patients both with and without psoriatic arthritis in comparison to a control healthy group. A number of studies have reported the roles of EVs as mediators of intercellular communication for pathophysiological processes in complex diseases [39,40]. Plaque psoriasis and psoriatic arthritis affect the skin and joints with underlying comorbidities affecting many other additional organs and the innate and adaptive immune systems. These diseases are no exception to exhibiting a complex network of intercellular communication. The results of this study suggest that signaling via EVs in the blood serum could be involved in the pathophysiological processes of plaque psoriasis and psoriatic arthritis. Accordingly, we discovered several EV-bound miRNAs that were differentially enriched between plaque psoriasis (PsV) and psoriatic arthritis (PsA) patients as well as between controls (C). Furthermore, we report a significant over-representation of potentially disease-related metabolic and functional pathways comprising predicted target genes of the differentially enriched miRNAs. Profiling other biomarker-like attributes of blood serum EVs was performed by phenotyping the surface proteome and measuring the physical properties of the blood serum EVs.

While we observed no changes in the overall EV-bound small RNA profile in the samples, distinguishable enrichment profiles were visible at the miRNA level for plaque psoriasis and PsA patient groups in comparison to controls. Compared with previous studies involving whole plasma or serum [21,22,41,42], we noticed little overlap in the miRNAs differentially enriched in the blood serum EVs from plaque psoriasis and PsA patients. This was in line with our hypothesis that we could identify distinct biomarker candidates specific to EVs. The current study found a total of 212 different EV-bound miRNAs in the analyzed blood serum EV samples. The highest number of differentially enriched miRNAs emerged from the comparison of plaque psoriasis and controls (12 miRNAs at FDR ≤ 0.05). We observed reduced amounts of hsa-miR-99b-5p in PsV samples—an miRNA that has previously been described to be downregulated in psoriatic skin, associated with keratinocyte hyperproliferation [43]. In contrast, we observed that hsa-miR-423-5p and hsa-miR-335-5p, whose downregulation has also been associated with abnormalities of keratinocyte differentiation and proliferation [43,44], were enriched in plaque psoriasis blood serum EVs compared to controls. The function of these EV-bound miRNAs in the blood serum remains uncertain in contrast to skin tissue in which they have been described. Furthermore, our observations of elevated hsa-miR-423 and hsa-miR-342 abundance in the blood serum EVs of plaque psoriasis patients have previously been linked to psoriasis-related comorbidities such as cardiovascular conditions [45,46] and irritable bowel syndrome [47]. These findings conform to the hypothesis that EVs circulating in the blood serum seem to be chauffeuring a mix of potentially psoriasis-related signals.

From the comparison of PsA and plaque psoriasis groups, we found several differentially enriched miRNAs among the FDR ≤ 0.1 results to be previously associated with osteoarthritis (OA) or rheumatoid arthritis (RA). For example, hsa-miR-671-3p was diminished by more than 2-fold in the blood serum EVs of PsA patients compared to the PsV group. Previously, it was shown that reduced levels of hsa-miR-671-3p in osteoarthritic knee cartilage led to increased inflammation and cartilage degradation [48,49]. It has also been shown that OA patients’ serum levels of hsa-miR-671-3p are reduced compared to controls [50]. We found hsa-miR-33a-5p to be enriched 1.8-fold in the PsA group compared to PsV. This miRNA has been implicated as a modulator of OA-associated genes in chondrocytes and osteoblasts [51]. In addition, we observed the enrichment of hsa-miR-26a-5p and hsa-miR-338-5p, which have previously been shown to induce proliferation, viability, and invasion of fibroblast-like synoviocytes in RA via the regulation of the PTEN/PI3K/AKT pathway [52] and the NFAT5 transcription factor [53], respectively. These results reveal an EV-bound miRNA signature in the blood serum of PsA patients that potentially reflects the pathological processes taking place in the affected joints.

Comparing PsA with the control group revealed similarities between the observed serum EV-bound miRNA profile in the PsA group and those previously reported observations in RA, but not in OA. We observed a 2-fold reduction in EV-bound hsa-miR-10a-5p and hsa-miR-34a-5p in the PsA group compared to controls. Increased proliferation and inflammation have been observed for synoviocytes with reduced expression of hsa-miR-10a-5p and hsa-miR-34a-5p in RA [54,55], whereas in OA, increased expression of these miRNAs induces chondrocyte apoptosis and contributes to severity of the disease [56,57]. In addition, reduced amounts of circulating hsa-miR-10b-5p in the blood serum have been previously reported in a case of RA compared to OA [58]. These similarities between PsA and RA are in line with the current knowledge of these diseases as they are both driven by autoimmunity in contrast to OA [59]. In summary, all three differences between the circulatory EV-bound miRNA profiles between patient groups and controls in this study seem to reflect pathological processes within the joints, revealing blood serum EVs to be potential biomarkers for PsA and PsV.

Pathway analysis of the genes targeted by differentially enriched miRNAs in the plaque psoriasis group revealed known core psoriasis processes, including class I MHC-mediated antigen processing and presentation, an integral part of psoriasis pathogenesis that presents autoantigens to CD8+ T cells [60]. Furthermore, the ubiquitin-proteasome pathway was found among the top affected pathways, which is a relatively new addition to the known regulatory network underpinning psoriasis [61]. The target genes of differentially enriched miRNAs in the comparison of PsA and PsV groups indicate transcriptional regulation by methyl CpG binding protein 2 (MeCP2) and the circadian rhythm. MeCP2 has previously been shown to be involved in transcriptional regulation that induced the activation of fibroblast-like synoviocytes in rat models of RA [62,63]. Interestingly, temporal variations have also been previously described in RA [64], and the influence of circadian clock has also been proposed in the pathogenesis of psoriasis as well [65]. There was also a noteworthy association found when comparing PsA to controls using targets of the differentially enriched miRNAs, with the mechanism of dopamine release cycle being the most significantly targeted. Higher levels of dopamine have been found to be a risk factor for psoriasis with involvement in the T cell activation process [66]. Furthermore, a connection between the dopaminergic pathway and RA was also observed [67] in a recent in vitro study revealing the inducing effect of dopamine on the migration of synovial fibroblasts [68]. While uncovering the specific mechanisms behind these reported associations remained out of scope for this study, these in silico-revealed pathway-level associations provide further confidence in the involvement of circulating EVs in the mediation of intercellular communication in PsV and PsA.

In a similar study, Pasquali and colleagues recently investigated the miRNA profile of circulatory EVs in plaque psoriasis and PsA patients for early biomarkers of PsA, resulting in several potential biomarker candidates such as hsa-miR-23a-3p, hsa-miR-26a-5p and hsa-miR-27a-3p [69]. Though we observed the differential enrichment of several miRNAs implicated in the immunopathology of joints, only one miRNA (hsa-miR-27a-3p) was common to both studies when comparing psoriasis patients with and without PsA. We previously observed that biomarker discovery studies with relatively small patient population might be prone to variation due to distinct subsets being described [70]; however, in this case these discrepancies are more likely due to the different methods of EV purification employed in the two studies [71]. Indeed, as different strategies of EV purification favor different subsets of EVs and are affected by different sources of impurities, this would also resonate in the distinct subset of EV-bound miRNAs that are intercepted. In this study, we have taken measures to confirm the enrichment and purification of EVs, which allows for more confidence in considering the detected miRNA as truly EV-bound.

Various EV purification and enrichment strategies have been developed in respect to different biofluids and experimental aims, but no specific strategy has surfaced as the gold standard method [72]. The choice of method will impact the composition of the enriched EV population, isolation yield and purity [71,73]. EV purification from blood serum can be considered notably challenging as researchers have to weigh yield against purity to rid blood serum samples of contaminating LDL [74], HDL [38] and aggregated protein particles [75]. In the case of blood plasma, two-step EV purification methods have demonstrated high purity, but at the expense of a low yield [37,76]. In this study, we were constrained by the amount of available sample volume and we used size-exclusion chromatography (SEC) as an efficient single-step isolation method to purify EVs with a balanced ratio of yield to purity [77,78]. Western blot analysis confirmed the enrichment of serum-derived EVs with common EV surface marker proteins (CD9, CD63 and CD81) and the reduction of ApoA1 and albumin, which were used to measure HDL co-isolation and protein contamination, respectively. The presence of such EV surface proteins indicates that EVs were successfully isolated using SEC, and the EV samples were pure and of sufficient yield for downstream applications.

In addition to EV-bound miRNAs, we screened the physical characteristics of the isolated EVs, such as their size, ZP and surface proteome, to find potential biomarkers in plaque psoriasis and psoriatic arthritis. The size distributions of serum EVs and their ZP profile can be viewed as proxy biomarkers with the presence of changes in the size distributions of EVs revealing the state of their origin cells [32,79]. The ZP value of EVs, which is related to their surface charge, is in turn affected by the protein composition of its lipid bilayer [80]. In this study, we did not detect a significant change in the size profiles or ZP values of blood serum EVs between the patient groups. The circulating populations of EVs in the blood are known to be dynamic and heterogeneous [81,82], making it challenging to detect genuine effects among the heterogeneity and variance. Another possible explanation for this might be that the long-term storage effect of samples on the EVs’ stability can influence the biological surface properties of EVs and their cargo proteins, thus indirectly affecting the ZP values of EVs’ colloidal stability [83,84]. Further research is required to develop more sensitive technologies that can bring more clarity to the biomarker potential of physical characteristics of EVs such as size, ZP and even the total amount of circulating EVs [32,85,86].

As a more targeted approach of EV surface-derived biomarker discovery, we used EV Array to phenotype the surface proteome of the blood serum EVs—a method that has previously proven successful in case of various diseases [28,87]. As with other systemic diseases, changes in the surface proteome of circulatory EVs are expected in plaque psoriasis and psoriatic arthritis [88,89,90]. These changes in the surface proteome of EVs ultimately reflect the processes in their cells of origin that are undergoing active pathophysiological changes during disease. Our analysis of the surface markers found relative abundance of Alix and CD19 differentiated the most between the PsV and PsA group, whereas CD9 was the only marker to remain statistically significant at FDR ≤ 0.1 after correcting for multiple testing after comparing the PsV and the control group. CD9 is a well-characterized exosomal marker [91] that has more importantly been recognized as a modulator of inflammation in PBMCs [92]. CD9 levels in plasma have been shown to correlate with exosome abundance, as well as oxidative stress and immune activation [93]. These observations support the use of exosome-bound CD9 as a proxy marker for the underlying inflammation processes in PBMCs, which have been studied in the context of psoriasis [94].

Despite the identification of several differentially enriched EV-bound miRNAs in the PsA and PsV groups, this study was constrained by a number of limitations. This study was limited by its small sample size, male-to-female ratio and wide range of patient ages, which is also common in this type of study [69]. A major limitation of this study relates to understanding the long-term storage temperature effects of blood serum on the stability of purified EVs. Blood serum samples are stored at −80 °C storage conditions, but the long-term storage temperature of the samples can alter their concentration, proteins, nucleic acids, surface charge and EV function. For this reason, it is crucial to consider a wide range of factors that can impact EV stability before using them as potential biomarkers.

## 4. Materials and Methods

### 4.1. Patient Groups and Blood Serum Samples

Blood-serum samples from two distinct groups of patients and healthy controls were used in this study: (1) moderate-to-severe chronic plaque psoriasis patients without psoriatic arthritis (PsV); (2) chronic plaque psoriasis patients with psoriatic arthritis (PsA); (3) healthy controls without inflammatory immune-related skin disorders (C). Twelve age (±5 years) and sex-matched patients were selected from each group, forming a total of 36 patients enrolled in the study. The patients and controls were aged between 24 and 64 at the time of sampling, with the mean age of patients being 51 years. Each group consisted of 2 females and 10 males. More detailed characteristics of the patients and controls can be found in Table S1.

Blood-serum samples from these patients and controls were collected and stored as part of a biobanking initiative in the Dermatology Clinic of the Tartu University Hospital. Whole blood was collected into Z Serum Clot Activator vacutainers, and serum was separated during routine clinical analyses performed by the hospital laboratory services, with leftover serum being stored at −80 °C. The blood serum samples used in this study had been stored at −80 °C under strictly monitored conditions for up to 5 years prior to the start of the study. Sample collection and research were approved by the Ethics Review Committee on Human Research of the University of Tartu. All participants signed an informed consent form.

### 4.2. Purification of Extracellular Vesicles

EVs were purified from the blood serum samples using size-exclusion chromatography (SEC) with a preceding step of differential centrifugation. First, the frozen blood serum samples were thawed on ice. This was followed by differential centrifugation at 400× *g*, 2000× *g*, and 10,000× *g* for 10 min at each stage at 4 °C with the supernatants sequentially carried over to the next stage. The supernatant resulting from the final stage of centrifugation was loaded onto qEVoriginal/70 nm SEC columns (Izon Science, Oxford, UK) in two 500 μL aliquotes per patient sample to meet the optimum input volume requirements specified by the manufacturer. The SEC columns were eluted with 1x Dulbecco’s phosphate-buffered saline (DPBS, Sigma^®^ Life Science, St. Louis, MO, USA) and 500 μL fractions were collected as per manufacturer’s protocol. The EV-enriched fractions 7 to 10 were pooled and 100 μL aliquots from each EV sample was spared for nanoparticle tracking analysis (NTA) and stored at −80 °C. The rest of the sample volume was concentrated to approximately 200 μL using Amicon^®^ Ultra-2 centrifugal filter units (10 kDa cutoff, Merck Millipore, Darmstadt, Germany). The filters were centrifuged in a swing-bucket type rotor at 3000× *g* at 4 °C until the desired concentration of sample volume was achieved. EV purification procedures were performed according to International Society for Extracellular Vesicles (ISEV) guidelines [95].

### 4.3. Nanoparticle Tracking Analysis

The total nanoparticle (NP) concentration, size and zeta potential (ZP) profile in the purified EV samples was measured using an NTA instrument ZetaView^®^ PMX 110 (Particle Metrix GmbH, Inning am Ammersee, Bavaria, Germany) according to previously optimized methodology [96]. The unconcentrated aliquotes of EV samples were first thawed on ice and resuspended in a final volume of 200 µL of MQ to obtain an initial concentration of EVs in the range of 1 × 10^8^ particles/mL and a DPBS concentration of 1 mM. The samples were then incubated at 4 °C for 2 h on a BIOSAN Multi Bio RS-24 rotator (BioSan, Riga, Latvia) at 20 rpm. Following incubation, the samples were further diluted into a final volume of 1000 µL using 0.1 × DPBS in order to achieve the 1 mM concentration of the suspension medium. Lastly, the pH of samples was adjusted to 7.4 by adding 0.1 M HCl/NaOH. The pH was monitored with a SevenCompactTM pH/Ion S220 pH meter (Mettler-Toledo AG, Schwerzenbach, Switzerland).

Using the ZetaView^®^ PMX 110 NTA instrument, size and concentration of NPs were measured in 3 consecutive cycles at 11 frames per cycle with camera sensitivity of 85, shutter value of 70, and a framerate of 30. ZP was measured with the camera set to two stationary layers. The measurements took place at room temperature conditions and each sample was measured in three repeats. For subsequent data analysis, resulting measurements were averaged across technical repeats.

### 4.4. Western Blot Analysis

Western Blot analyses were conducted with neat samples, without preceding protein precipitation. In order to determine the appropriate sample input volume, the protein concentration was measured using a Quick Start™ Bradford Protein Assay (Bio-Rad, Berkeley, CA, USA) according to manufacturer’s protocol. The input sample volume was mixed with 6x nonreducing Laemmil buffer to a final buffer concentration of 1×. The mixture was heated for 5 min at 95 °C and loaded onto 12% SDS-PAGE gel for electrophoresis. Subsequently, the proteins were transferred onto PVDF membranes during a 25 min semi-dry transfer at 25 V. The membranes were then incubated in a blocking buffer consisting of 5% nonfat dry milk in PBS-Tween 0.05%. This was followed by an overnight incubation with primary antibodies in 5% milk-PBST solution at 4 °C. The membranes were subsequently washed using PBST and incubated with a secondary HRP-conjugated antibody for 1 h at room temperature. Lastly, membranes were washed with PBS-Tween 0.05% and incubated in ECL Select Western Blotting Detection Reagent (GE Healthcare, Buckinghamshire, UK) according to manufacturer’s protocol. Resulting chemiluminescence was captured with an ImageQuant RT ECL Imager (GE Healthcare, Buckinghamshire, UK).

The primary antibodies used in this study were: mouse anti-human CD9 antibody (1:250, sc-59140, Santa Cruz Biotechnology Inc., Dallas, TX, USA), mouse anti-human CD63 antibody (1:1000, 556019, BD Biosciences, Franklin Lakes, NJ, USA), mouse anti-human CD81 antibody (1:500, 555675, BD Biosciences, Franklin Lakes, NJ, USA), mouse anti-human apoA-I antibody (1:100, sc-376818, Santa Cruz Biotechnology Inc., Dallas, TX, USA), and rabbit anti-human albumin antibody (1:10,000, 16475-1-AP, ProteinTech Group, Rosemont, IL, USA). The secondary antibodies used in this study were: HRP-conjugated goat anti-mouse IgG antibody (1:10,000, G21040, Thermo Fisher Scientific, Eugene, OR, USA) and goat anti-rabbit IgG antibody (1:2000, NIF824, GE Healthcare, Buckinghamshire, UK).

### 4.5. Transmission Electron Microscopy

Aliquotes of concentrated EV samples were first fixed by mixing the samples 1:1 with 4% paraformaldehyde in DPBS. Fixed samples were transported on dry ice to the Polish Academy of Sciences in Olsztyn, Poland, for transmission electron microscopy (TEM) imaging. Fixed samples were contrasted in uranyl oxalate solution consisting of 4% uranyl acetate (Polysciences, Warrington, PA, USA) and 0.15 M oxalic acid (Sigma-Aldrich, Schnelldorf, Germany). The samples were subsequently embedded in a mixture of methylcellulose (Sigma-Aldrich, Schnelldorf, Germany) and uranyl acetate (Polysciences, Warrington, PA, USA). Embedded samples were imaged with a JEM 1400 transmission electron microscope (JEOL Ltd., Tokyo, Japan) at 80 kV, and digital images were acquired with numeric Morada TEM CCD camera, (Olympus, Hamburg, Germany).

### 4.6. Multiplexed Phenotyping of EVs by EV Array

EV Array is a microarray-based technology for multiplexed phenotyping of EVs. The screening of blood serum EVs with the EV Array was performed according to previously described methodology [97]. Unprocessed aliquotes of the blood serum samples used in this study were refrozen at −80 °C and transported on dry ice to the EV Array facilities in Aalborg University Hospital, Denmark. The blood serum input volume for EV Array was 75 µL; however, in two of the samples (C1 and C2), the input volume measured at 45 µL due to limited amount of source material. This difference in input volumes was later accounted for in the data analysis stage. A total of 30 antibodies (Table S2) were chosen for EV phenotyping from a panel of readily available antibodies by narrowing the selection to proteins that have been associated with the pathophysiology of psoriasis or arthritis in previously published research papers.

### 4.7. Small RNA Sequencing

RNA was extracted from concentrated EV samples (~200 µL) immediately following the purification of EVs. Small RNAs were favored in the process of RNA extraction, and this was facilitated by using the miRNeasy Micro Kit in combination with RNeasy MinElute spin columns (Qiagen, Hilden, Germany). A modified version of the manufacturer’s protocol that has been optimized for exosomal RNA [98] was followed. Extracted RNA was eluted into a final volume of 25 µL of nuclease-free water. The yield of RNA was first quantified by measuring total RNA concentration using a Qubit™ 2.0 fluorometer (Thermo Fisher Scientific, Eugene, OR, USA). Qubit™ RNA HS Assay Kit was used to prepare the samples for measurement according to a spike-in protocol [99] aiming to improve the quantification limit of the method. In addition, the size profile of RNA samples was measured with an Agilent 2100 Bioanalyzer (Agilent Technologies, Santa Clara, CA, USA) instrument using the Agilent RNA 6000 Pico Kit. Next, the RNA samples were mixed with RNA stable^®^ (Biomatrica, San Diego, CA, USA) and dehydrated in an Eppendorf Concentrator Plus vacuum centrifuge (Sigma-Aldrich, Schnelldorf, Germany). The samples were then transported in a moisture-sealed environment to the sequencing facilities in Sheffield University, UK.

Libraries for small RNA sequencing were prepared using the RealSeq Biofluids kit (SomaGenics Inc., Santa Cruz, CA, USA) and following the manufacturer’s protocol. Twenty PCR cycles were performed to amplify the reverse-transcripted cDNA. The resulting libraries were examined using the Agilent 2100 Bioanalyzer with the Agilent High Sensitivity DNA kit prior to the individual libraries being pooled to a concentration of 4 nM for sequencing. Sequencing was carried out on an Illumina HiSeq 2500 (Illumina Inc., San Diego, CA, USA) using v3 chemistry producing single-end 50 bp reads.

### 4.8. Statistical Analysis

#### 4.8.1. NTA Data

The size and ZP profiles were first normalized to adjust for differences in the total NP concentration of samples by dividing counts per size or ZP bin by the total number of counts across all bins, resulting in a value of proportion of total counts per bin. We used 20 nm bin sizes for NP size data and used 5 mV bins in the case of ZP data. We derived 95% confidence intervals from a t-distribution (df = N − 1) using the qt() function in R [100]. Density curves were derived using the density() function in R with default parameters.

#### 4.8.2. EV Array Data

Measurements with intensity values equal to or less than background levels were excluded from analysis. In order for a quantified protein marker to be retained in the analysis, at least 6 (50%) samples in at least one of the patient groups were required to pass the set threshold. The statistical significance of the differences in intensity values between the patient groups was tested for by fitting a linear model with the intensity value as the response variable and patient group as the predictor, adjusted for blood-serum-sample storage time. Individual pairwise models were constructed for patient groups and protein markers remaining in the analysis. Resulting *p*-values of the patient-group terms were adjusted for multiple testing with the Benjamini–Hochberg procedure across all comparisons of patient groups and markers. False discovery rate (FDR) cut-off values of 0.05 and 0.1 were both considered when interpreting the results. For the purpose of hierarchical clustering and heatmap visualization, measured intensity values were adjusted for storage time by substituting the actual measurements with residuals from linear models with protein marker intensity values as the response variable and storage time as the predictor. Linear models were fitted using the lm() function in R.

In the two samples with lesser input volume, multipliers were used to adjust the measured intensity values. The multipliers were derived by fitting a logarithmic function to measurements of sequential dilutions of positive control samples included on the microarray for each antibody.

#### 4.8.3. Small RNA Sequencing Data

Raw reads were first inspected by FastQC v0.11.9 (FastQC, Babraham Bioinformatics, Cambridge, UK ) [101] followed by trimming of adapter sequences and low quality bases using Trimmomatic v0.39 [102] with the following parameters: adapters.fa:2:30:10 SLIDINGWINDOW:3:15 LEADING:3 TRAILING:3 MINLEN:15. Remaining reads were mapped to the GRCh38.p13 human reference genome primary assembly using Bowtie2 v2.4.2 (Bowtie) [103], with no mismatches allowed in the seed alignment but two mismatches allowed in the rest of the sequence. Only primary alignments with mapping quality (MAPQ) ≥ 10 were retained for further analysis. A custom annotation file was constructed by merging protein-coding exon elements from the GENCODE [104] GRCh38 primary assembly annotation release 35 with non-coding RNA annotations obtained from RNAcentral [105]. Counts were summarized using HTSeq v0.12.4 [106] and “fraction counts” principle was applied, i.e., read alignments with overlapping annotations were equally divided between the RNA elements by 1/n, where n is the number of overlapping annotations on top of the alignment.

Differential miRNA enrichment analysis was conducted based on counts obtained for miRNA annotations. In order for an miRNA to be retained for differential enrichment analysis, at least 6 (50%) samples in at least one of the patient groups were required to pass the threshold of 5.0 counts. Differential enrichment analysis was conducted in R using the “edgeR-robust” approach [107]. False discovery rate (FDR) cut-off values of 0.05 and 0.1 were both considered when interpreting the results.

The analysis of pathway-level targeted processes was conducted using the subset of miRNAs found to be differentially enriched at FDR ≤ 0.1 in the respective comparison of patient groups. For this set of miRNAs, predicted target genes with a score of ≥90 were obtained from the miRDB database v6.0 [108]. Pathway enrichment analysis was then performed on the obtained sets of targeted genes using the enrichPathway() function of the ReactomePA package (ReactomePA) [109] in R, which is based on a hypergeometric test approach and annotations from Reactome Pathway Database [110]. Pathways enriched at FDR ≤ 0.05 were considered significant.

#### 4.8.4. Data Visualization

All of the graphs were generated using ggplot2 package [111] in R, with the exception of heatmaps, for which the ComplexHeatmap [112] package was used. Principal components were calculated using the prcomp() function in R with the input data being standardized (converted to z-scores) beforehand. For the EV Array data, Euclidean distance was used for hierarchical clustering, whereas Manhattan distance was used for sequencing counts data.

### 4.9. Experimental Design

#### 4.9.1. Investigating the Physical Characteristics of Blood Serum-Derived EVs

Samples from 36 individuals including PsA (*n* = 12) and PsV patients (*n* = 12), and control (*n* = 12) were investigated. To determine the physical characteristics of blood serum-derived EVs, the size distribution and ZP values of both EVs in the blood serum of PsA and PsV were compared with a control group. The size and ZP of the blood serum-derived EVs (PsA, PsV and control groups) purified utilizing SEC were measured using ZetaView^®^ PMX 110 NTA instrument. Experiments were performed in triplicate, and the size, concentration and ZP of EVs were measured at 25 °C.

#### 4.9.2. EV miRNA and Surface Proteome Profiling of Patient and Control Groups

Blood serum-derived EVs were subjected to RNA extraction and sequencing for patients and control groups. Differential enrichment analysis of miRNAs was performed and compared among the following groups: PsA vs. C, PsV vs. C and PsA vs. PsV. Similar group comparisons were also conducted for EV surface proteomes using the EV Array platform.

## 5. Conclusions

This study set out to characterize and compare the small RNA contents and surface proteome of the EVs in the blood serum of PsV and PsA patients to healthy controls. The results of this investigation show the identification of several differentially enriched EV-bound miRNAs in the PsA and PsV groups with previous associations with inflammatory skin diseases and joint inflammation. Using EV Array, the current study also found the presence of EV surface markers in the PsA and PsV groups. The EV array revealed that PsV patient EV samples were significantly enriched with CD9 EV-marker compared to controls. Taken together, these findings provide insight into circulating EVs and their cargo-carrying signals that can modulate the pathogenesis of plaque psoriasis and psoriatic arthritis. Circulating EVs and their cargo offer insight into the pathophysiology of diseases, along with the discovery of potential biomarkers for therapeutic interventions [88]. The insight gained from this study may be of assistance to EV research, such as the EV array and different microfluidics solutions [113], eliminating the need for intricate EV purification techniques, EV research could yield early markers of PsA and other psoriasis-related comorbidities and potentially enable monitoring of the progression of the disease and efficacy of treatment. With these solutions delivered to the bedside of the patient, EVs could be the key to filling the void in clinically applicable diagnostic and prognostic biomarkers of psoriasis and psoriatic arthritis.

## Figures and Tables

**Table 1:** Results of miRNA differential enrichment testing. Results are limited to FDR ≤ 0.1.

Comparison	miRNA	FC	*p*-Value	FDR
PsA to PsV	QXBT12	0.46	0.00053	0.058
	hsa-miR-33a-5p	1.82	0.000547	0.058
	hsa-miR-26a-5p	1.36	0.00101	0.0596
	hsa-miR-mizuguchi-225	1.81	0.00113	0.0596
	miTC3	0.43	0.00242	0.0879
	hsa-miR-338-5p	2.32	0.00273	0.0879
	hsa-miR-671-3p	0.43	0.0029	0.0879
	hsa-miR-342-3p	0.52	0.00375	0.0993
PsA to C	hsa-miR-10b-5p	0.46	0.000204	0.0433
	hsa-miR-197-3p	0.53	0.000873	0.0811
	hsa-miR-425-5p	1.51	0.00128	0.0811
	hsa-miR-199a-5p	1.57	0.00153	0.0811
	hsa-miR-203a-3p	0.35	0.002	0.0849
	hsa-miR-10a-5p	0.54	0.0025	0.0882
	hsa-miR-34a-5p	0.46	0.00292	0.0884
PsV to C	hsa-miR-423-5p	2.09	3.58 × 10^−5^	0.00419
	hsa-miR-335-5p	2.29	6.08 × 10^−5^	0.00419
	hsa-miR-342-3p	2.57	6.52 × 10^−5^	0.00419
	miTC1	2.90	7.91 × 10^−5^	0.00419
	has-miR-425-5p	1.62	0.000183	0.00775
	hsa-miR-99b-5p	0.56	0.000898	0.0317
	hsa-miR-17-5p	1.44	0.00148	0.0384
	hsa-miR-18a-5p	1.55	0.00176	0.0384
	hsa-miR-27a-3p	0.72	0.0018	0.0384
	hsa-miR-451a	1.77	0.00181	0.0384
	QXBT12	2.01	0.00204	0.0394
	miTC3	2.48	0.00279	0.0493
	hsa-miR-6803-3p	1.94	0.00459	0.0697
	hsa-miR-199a-5p	1.50	0.0046	0.0697
	hsa-miR-30b-5p	0.67	0.00554	0.0783
	hsa-miR-20a-5p	1.37	0.00595	0.0789
	hsa-miR-340-3p	1.93	0.00641	0.0799
	hsa-miR-30d-5p	0.71	0.00728	0.0858

## Data Availability

The authors confirm that the data supporting the findings of this study are available in the Supplementary Materials. Analyzed data are either presented in the article or can be provided upon request.

## Supplementary Materials

The following supporting information can be downloaded at: https://www.mdpi.com/article/10.3390/ijms23074005/s1.

## References

[1] Ocampo D.V., Gladman D. (2019). Psoriatic arthritis. F1000Res.

[2] Gudu T., Gossec L. (2018). Quality of life in psoriatic arthritis. Expert Rev. Clin. Immunol..

[3] Coates L.C., Helliwell P.S. (2017). Psoriatic arthritis: State of the art review. Clin. Med..

[4] Haroon M., Gallagher P., FitzGerald O. (2015). Diagnostic delay of more than 6 months contributes to poor radiographic and functional outcome in psoriatic arthritis. Ann. Rheum. Dis..

[5] Villani A.P., Rouzaud M., Sevrain M., Barnetche T., Paul C., Richard M.A., Beylot-Barry M., Misery L., Joly P., Le Maitre M. (2015). Prevalence of undiagnosed psoriatic arthritis among psoriasis patients: Systematic review and meta-analysis. J. Am. Acad. Dermatol..

[6] Griffiths C.E., Barker J.N. (2007). Pathogenesis and clinical features of psoriasis. Lancet.

[7] Dastoli S., Nisticò S.P., Morrone P., Patruno C., Leo A., Citraro R., Gallelli L., Russo E., De Sarro G., Bennardo L. (2022). Colchicine in managing skin conditions: A systematic review. Pharmaceutics.

[8] Iannone L.F., Bennardo L., Palleria C., Roberti R., De Sarro C., Naturale M.D., Dastoli S., Donato L., Manti A., Valenti G. (2020). Safety profile of biologic drugs for psoriasis in clinical practice: An Italian prospective pharmacovigilance study. PLoS ONE.

[9] Christophers E. (2001). Psoriasis—Epidemiology and clinical spectrum. Clin. Exp. Dermatol..

[10] Mc Ardle A., Flatley B., Pennington S.R., FitzGerald O. (2015). Early biomarkers of joint damage in rheumatoid and psoriatic arthritis. Arthritis Res. Ther..

[11] Generali E., Scirè C.A., Favalli E.G., Selmi C. (2016). Biomarkers in psoriatic arthritis: A systematic literature review. Expert Rev. Clin. Immunol..

[12] Selleck M.J., Senthil M., Wall N.R. (2017). Making meaningful clinical use of biomarkers. Biomark. Insights.

[13] Jiang S., Hinchliffe T.E., Wu T. (2015). Biomarkers of an autoimmune skin disease—Psoriasis. Genom. Proteom. Bioinform..

[14] Punzi L., Poswiadek M., Oliviero F., Lonigro A., Modesti V., Ramonda R., Todesco S. (2011). Laboratory findings in psoriatic arthritis. Reumatismo.

[15] Sokolova M.V., Simon D., Nas K., Zaiss M.M., Luo Y., Zhao Y., Rech J., Schett G. (2020). A set of serum markers detecting systemic inflammation in psoriatic skin, entheseal, and joint disease in the absence of C-reactive protein and its link to clinical disease manifestations. Arthritis Res. Ther..

[16] Tamagawa-Mineoka R., Katoh N., Kishimoto S. (2010). Platelet activation in patients with psoriasis: Increased plasma levels of platelet-derived microparticles and soluble P-selectin. J. Am. Acad. Dermatol..

[17] Pamuk G.E., Pamuk N., Orüm H., Arican O., Turgut B., Demir M. (2009). Elevated platelet-monocyte complexes in patients with psoriatic arthritis. Platelets.

[18] Arican O., Aral M., Sasmaz S., Ciragil P. (2005). Serum levels of TNF-alpha, IFN-gamma, IL-6, IL-8, IL-12, IL-17, and IL-18 in patients with active psoriasis and correlation with disease severity. Mediators Inflamm..

[19] Bosè F., Capsoni F., Molteni S., Raeli L., Diani M., Altomare A., Garavaglia M., Garutti C., Frigerio E., Banfi G. (2014). Differential expression of interleukin-2 by anti-CD3-stimulated peripheral blood mononuclear cells in patients with psoriatic arthritis and patients with cutaneous psoriasis. Clin. Exp. Dermatol..

[20] Alenius G.-M., Eriksson C., Rantapää Dahlqvist S. (2009). Interleukin-6 and soluble interleukin-2 receptor alpha-markers of inflammation in patients with psoriatic arthritis?. Clin. Exp. Rheumatol..

[21] Xiao S., Liu X., Wang X., Lv H., Zhao J., Guo X., Xian F., Ji Y., Zhang G. (2020). Plasma MicroRNA expression profiles in psoriasis. J. Immunol. Res..

[22] Wade S.M., McGarry T., Wade S.C., Fearon U., Veale D.J. (2020). Serum MicroRNA signature as a diagnostic and therapeutic marker in patients with psoriatic arthritis. J. Rheumatol..

[23] Alberro A., Iparraguirre L., Fernandes A., Otaegui D. (2021). Extracellular vesicles in blood: Sources, effects, and applications. Int. J. Mol. Sci..

[24] Palviainen M., Saraswat M., Varga Z., Kitka D., Neuvonen M., Puhka M., Joenväärä S., Renkonen R., Nieuwland R., Takatalo M. (2020). Extracellular vesicles from human plasma and serum are carriers of extravesicular cargo-Implications for biomarker discovery. PLoS ONE.

[25] El Andaloussi S., Mäger I., Breakefield X.O., Wood MJ A. (2013). Extracellular vesicles: Biology and emerging therapeutic opportunities. Nat. Rev. Drug. Discov..

[26] He X., Park S., Chen Y., Lee H. (2021). Extracellular vesicle-associated miRNAs as a biomarker for lung cancer in liquid biopsy. Front. Mol. Biosci..

[27] Probert C., Dottorini T., Speakman A., Hunt S., Nafee T., Fazeli A., Wood S., Brown J.E., James V. (2019). Communication of prostate cancer cells with bone cells via extracellular vesicle RNA; a potential mechanism of metastasis. Oncogene.

[28] Mørk M., Andreasen J.J., Rasmussen L.H., Lip G.Y., Pedersen S., Bæk R., Jørgensen M.M., Kristensen S.R. (2019). Elevated blood plasma levels of tissue factor-bearing extracellular vesicles in patients with atrial fibrillation. Thromb. Res..

[29] Gidlöf O., Evander M., Rezeli M., Marko-Varga G., Laurell T., Erlinge D. (2019). Proteomic profiling of extracellular vesicles reveals additional diagnostic biomarkers for myocardial infarction compared to plasma alone. Sci. Rep..

[30] Badhwar A., Haqqani A.S. (2020). Biomarker potential of brain-secreted extracellular vesicles in blood in Alzheimer’s disease. Alzheimers Dement..

[31] Jiang M., Fang H., Shao S., Dang E., Zhang J., Qiao P., Yang A., Wang G. (2019). Keratinocyte exosomes activate neutrophils and enhance skin inflammation in psoriasis. FASEB J..

[32] Mangino G., Iuliano M., Carlomagno S., Bernardini N., Rosa P., Chiantore M.V., Skroza N., Calogero A., Potenza C., Romeo G. (2019). Interleukin-17A affects extracellular vesicles release and cargo in human keratinocytes. Exp. Dermatol..

[33] Marton N., Kovács O.T., Baricza E., Kittel Á., Győri D., Mócsai A., Meier FM P., Goodyear C.S., McInnes I.B., Buzás E.I. (2017). Extracellular vesicles regulate the human osteoclastogenesis: Divergent roles in discrete inflammatory arthropathies. Cell. Molec. Life Sci..

[34] Andreu Z., Rivas E., Sanguino-Pascual A., Lamana A., Marazuela M., González-Alvaro I., Sánchez-Madrid F., de la Fuente H., Yáñez-Mó M. (2016). Comparative analysis of EV isolation procedures for miRNAs detection in serum samples. J. Extracell. Vesicles.

[35] Endzeliņš E., Berger A., Melne V., Bajo-Santos C., Soboļevska K., Ābols A., Rodriguez M., Šantare D., Rudņickiha A., Lietuvietis V. (2017). Detection of circulating miRNAs: Comparative analysis of extracellular vesicle-incorporated miRNAs and cell-free miRNAs in whole plasma of prostate cancer patients. BMC Cancer.

[36] Ramshani Z., Zhang C., Richards K., Chen L., Xu G., Stiles B.L., Hill R., Senapati S., Go D.B., Chang H.-C. (2019). Extracellular vesicle microRNA quantification from plasma using an integrated microfluidic device. Commun. Biol..

[37] Karimi N., Cvjetkovic A., Jang S.C., Crescitelli R., Hosseinpour Feizi M.A., Nieuwland R., Lötvall J., Lässer C. (2018). Detailed analysis of the plasma extracellular vesicle proteome after separation from lipoproteins. Cell. Molec. Life Sci. CMLS.

[38] Yuana Y., Levels J., Grootemaat A., Sturk A., Nieuwland R. (2014). Co-isolation of extracellular vesicles and high-density lipoproteins using density gradient ultracentrifugation. J. Extracell. Vesicles.

[39] Berumen Sánchez G., Bunn K.E., Pua H.H., Rafat M. (2021). Extracellular vesicles: Mediators of intercellular communication in tissue injury and disease. Cell. Commun. Signal..

[40] Pitt J.M., Kroemer G., Zitvogel L. (2016). Extracellular vesicles: Masters of intercellular communication and potential clinical interventions. J. Clin. Invest..

[41] Pelosi A., Lunardi C., Fiore P.F., Tinazzi E., Patuzzo G., Argentino G., Moretta F., Puccetti A., Dolcino M. (2018). MicroRNA expression profiling in psoriatic arthritis. Biomed. Res. Int..

[42] Koga Y., Jinnin M., Ichihara A., Fujisawa A., Moriya C., Sakai K., Fukushima S., Inoue Y., Ihn H. (2014). Analysis of expression pattern of serum microRNA levels in patients with psoriasis. J. Dermatol. Sci..

[43] Lerman G., Avivi C., Mardoukh C., Barzilai A., Tessone A., Gradus B., Pavlotsky F., Barshack I., Polak-Charcon S., Orenstein A. (2011). MiRNA expression in psoriatic skin: Reciprocal regulation of hsa-miR-99a and IGF-1R. PLoS ONE.

[44] Liew W.C., Sundaram G.M., Quah S., Lum G.G., Tan J.S.L., Ramalingam R., Common J.E.A., Tang M.B.Y., Lane E.B., Thng S.T.G. (2020). Belinostat resolves skin barrier defects in atopic dermatitis by targeting the dysregulated miR-335:SOX6 axis. J. Allergy Clin. Immunol..

[45] Tijsen A.J., Creemers E.E., Moerland P.D., de Windt L.J., van der Wal A.C., Kok W.E., Pinto Y.M. (2010). MiR423-5p as a circulating biomarker for heart failure. Circ. Res..

[46] Rizzacasa B., Morini E., Mango R., Vancheri C., Budassi S., Massaro G., Maletta S., Macrini M., D’Annibale S., Romeo F. (2019). MiR-423 is differentially expressed in patients with stable and unstable coronary artery disease: A pilot study. PLoS ONE.

[47] Fourie N.H., Peace R.M., Abey S.K., Sherwin L.B., Rahim-Williams B., Smyser P.A., Wiley J.W., Henderson W.A. (2014). Elevated circulating miR-150 and miR-342-3p in patients with irritable bowel syndrome. Exp. Mol. Pathol..

[48] Hu G., Zhang N., Li J., Wang J., Wu W., Li J., Tong W., Zhao X., Dai L., Zhang X. (2020). Tumor necrosis factor receptor associated factor 3 modulates cartilage degradation through suppression of Interleukin 17 signaling. Am. J. Pathol..

[49] Liu Z., Chen S., Yang Y., Lu S., Zhao X., Hu B., Pei H. (2019). MicroRNA-671-3p regulates the development of knee osteoarthritis by targeting TRAF3 in chondrocytes. Mol. Med. Rep..

[50] Ntoumou E., Tzetis M., Braoudaki M., Lambrou G., Poulou M., Malizos K., Stefanou N., Anastasopoulou L., Tsezou A. (2017). Serum microRNA array analysis identifies miR-140-3p, miR-33b-3p and miR-671-3p as potential osteoarthritis biomarkers involved in metabolic processes. Clin. Epigenetics.

[51] Costa V., De Fine M., Carina V., Conigliaro A., Raimondi L., De Luca A., Bellavia D., Salamanna F., Alessandro R., Pignatti G. (2021). How miR-31-5p and miR-33a-5p regulates SP1/CX43 expression in osteoarthritis disease: Preliminary insights. Int. J. Mol. Sci..

[52] Huang Z., Xing S., Liu M., Deng W., Wang Y., Huang Z., Huang Y., Huang X., Wu C., Guo X. (2019). MiR-26a-5p enhances cells proliferation, invasion, and apoptosis resistance of fibroblast-like synoviocytes in rheumatoid arthritis by regulating PTEN/PI3K/AKT pathway. Biosci. Rep..

[53] Guo T., Ding H., Jiang H., Bao N., Zhou L., Zhao J. (2018). miR-338-5p regulates the viability, proliferation, apoptosis and migration of rheumatoid arthritis fibroblast-like synoviocytes by targeting NFAT5. Cell. Physiol. Biochem..

[54] Hussain N., Zhu W., Jiang C., Xu J., Geng M., Wu X., Hussain S., Wang B., Rajoka M.S.R., Li Y. (2018). Down-regulation of miR-10a-5p promotes proliferation and restricts apoptosis via targeting T-box transcription factor 5 in inflamed synoviocytes. Biosci. Rep..

[55] Song A.-F., Kang L., Wang Y.-F., Wang M. (2020). MiR-34a-5p inhibits fibroblast-like synoviocytes proliferation via XBP1. Eur. Rev. Med. Pharmacol. Sci..

[56] Li H.-Z., Xu X.-H., Lin N., Wang D.-W., Lin Y.-M., Su Z.-Z., Lu H.-D. (2020). Overexpression of miR-10a-5p facilitates the progression of osteoarthritis. Aging.

[57] Endisha H., Datta P., Sharma A., Nakamura S., Rossomacha E., Younan C., Ali S.A., Tavallaee G., Lively S., Potla P. (2021). MicroRNA-34a-5p promotes joint destruction during osteoarthritis. Arthritis Rheumatol..

[58] Rousseau J.-C., Millet M., Croset M., Sornay-Rendu E., Borel O., Chapurlat R. (2020). Association of circulating microRNAs with prevalent and incident knee osteoarthritis in women: The OFELY study. Arthritis Res. Ther..

[59] Mohammed A., Alshamarri T., Adeyeye T., Lazariu V., McNutt L.-A., Carpenter D.O. (2020). A comparison of risk factors for osteo- and rheumatoid arthritis using NHANES data. Prev. Med. Rep..

[60] Prinz J.C. (2018). Human leukocyte antigen-Class I alleles and the autoreactive T cell response in psoriasis pathogenesis. Front. Immunol..

[61] Yang L., Guo W., Zhang S., Wang G. (2018). Ubiquitination-proteasome system: A new player in the pathogenesis of psoriasis and clinical implications. J. Dermatol. Sci..

[62] Sun Z.-H., Liu Y., Liu J., Xu D., Li X., Meng X., Ma T., Huang C., Li J. (2017). MeCP2 regulates PTCH1 expression through DNA methylation in rheumatoid arthritis. Inflammation.

[63] Miao C., Huang C., Huang Y., Yang Y., He X., Zhang L., Lv X.-W., Jin Y., Li J. (2013). MeCP2 modulates the canonical Wnt pathway activation by targeting SFRP4 in rheumatoid arthritis fibroblast-like synoviocytes in rats. Cell. Signal..

[64] Gibbs J.E., Ray D.W. (2013). The role of the circadian clock in rheumatoid arthritis. Arthritis Res. Ther..

[65] Ando N., Nakamura Y., Aoki R., Ishimaru K., Ogawa H., Okumura K., Shibata S., Shimada S., Nakao A. (2015). Circadian gene clock regulates psoriasis-like skin inflammation in mice. J. Investig. Dermatol..

[66] Bain K.A., Milling S. (2019). T cell addiction: Can pathogenic T cells be controlled using dopamine receptors?. Immunology.

[67] Capellino S. (2020). Dopaminergic agents in rheumatoid arthritis. J. Neuroimmune. Pharmacol..

[68] Van Nie L., Salinas-Tejedor L., Dychus N., Fasbender F., Hülser M.-L., Cutolo M., Rehart S., Neumann E., Müller-Ladner U., Capellino S. (2020). Dopamine induces in vitro migration of synovial fibroblast from patients with rheumatoid arthritis. Sci. Rep..

[69] Pasquali L., Svedbom A., Srivastava A., Rosén E., Lindqvist U., Ståhle M., Pivarcsi A., Sonkoly E. (2020). Circulating microRNAs in extracellular vesicles as potential biomarkers for psoriatic arthritis in patients with psoriasis. J. Eur. Acad. Dermatol. Venereol..

[70] Reimann E., Lättekivi F., Keermann M., Abram K., Kõks S., Kingo K., Fazeli A. (2019). Multicomponent biomarker approach improves the accuracy of diagnostic biomarkers for psoriasis vulgaris. Acta Derm. Venereol..

[71] Helwa I., Cai J., Drewry M.D., Zimmerman A., Dinkins M.B., Khaled M.L., Seremwe M., Dismuke W.M., Bieberich E., Stamer W.D. (2017). A comparative study of serum exosome isolation using differential ultracentrifugation and three commercial reagents. PLoS ONE.

[72] Konoshenko M., Yu Lekchnov E.A., Vlassov A.V., Laktionov P.P. (2018). Isolation of extracellular vesicles: General methodologies and latest trends. Biomed. Res. Int..

[73] Buschmann D., Kirchner B., Hermann S., Märte M., Wurmser C., Brandes F., Kotschote S., Bonin M., Steinlein O.K., Pfaffl M.W. (2018). Evaluation of serum extracellular vesicle isolation methods for profiling miRNAs by next-generation sequencing. J. Extracell. Vesicles.

[74] Sódar B.W., Kittel Á., Pálóczi K., Vukman K.V., Osteikoetxea X., Szabó-Taylor K., Németh A., Sperlágh B., Baranyai T., Giricz Z. (2016). Low-density lipoprotein mimics blood plasma-derived exosomes and microvesicles during isolation and detection. Sci. Rep..

[75] Brennan K., Martin K., FitzGerald S.P., O’Sullivan J., Wu Y., Blanco A., Richardson C., Mc Gee M.M.A. (2020). A comparison of methods for the isolation and separation of extracellular vesicles from protein and lipid particles in human serum. Sci. Rep..

[76] Onódi Z., Pelyhe C., Terézia Nagy C., Brenner G.B., Almási L., Kittel Á., Manček-Keber M., Ferdinandy P., Buzás E.I., Giricz Z. (2018). Isolation of high-purity extracellular vesicles by the combination of iodixanol density gradient ultracentrifugation and bind-elute chromatography from blood plasma. Front. Physiol..

[77] Vogel R., Coumans F.A.W., Maltesen R.G., Böing A.N., Bonnington K.E., Broekman M.L., Broom M.F., Buzás E.I., Christiansen G., Hajji N. (2016). A standardized method to determine the concentration of extracellular vesicles using tunable resistive pulse sensing. J. Extracell. Vesicles.

[78] Navajas R., Corrales F.J., Paradela A. (2019). Serum exosome isolation by size-exclusion chromatography for the discovery and validation of preeclampsia-associated biomarkers. Methods Mol. Biol..

[79] Belov L., Matic K.J., Hallal S., Best O.G., Mulligan S.P., Christopherson R.I. (2016). Extensive surface protein profiles of extracellular vesicles from cancer cells may provide diagnostic signatures from blood samples. J. Extracell. Vesicles.

[80] Akagi T., Kato K., Kobayashi M., Kosaka N., Ochiya T., Ichiki T. (2015). On-chip immunoelectrophoresis of extracellular vesicles released from human breast cancer cells. PLoS ONE.

[81] Kowal J., Arras G., Colombo M., Jouve M., Morath J.P., Primdal-Bengtson B., Dingli F., Loew D., Tkach M., Théry C. (2016). Proteomic comparison defines novel markers to characterize heterogeneous populations of extracellular vesicle subtypes. Proc. Natl. Acad. Sci. USA.

[82] Matsumoto A., Takahashi Y., Chang H., Wu Y., Yamamoto A., Ishihama Y., Takakura Y. (2020). Blood concentrations of small extracellular vesicles are determined by a balance between abundant secretion and rapid clearance. J. Extracell. Vesicles.

[83] Santucci L., Bruschi M., Del Zotto G., Antonini F., Ghiggeri G.M., Panfoli I., Candiano G. (2019). Biological surface properties in extracellular vesicles and their effect on cargo proteins. Sci. Rep..

[84] Maroto R., Zhao Y., Jamaluddin M., Popov V.L., Wang H., Kalubowilage M., Zhang Y., Luisi J., Sun H., Culbertson C.T. (2017). Effects of storage temperature on airway exosome integrity for diagnostic and functional analyses. J. Extracell. Vesicles.

[85] Sharma S., LeClaire M., Wohlschlegel J., Gimzewski J. (2020). Impact of isolation methods on the biophysical heterogeneity of single extracellular vesicles. Sci. Rep..

[86] Chiang C.-Y., Chen C. (2019). Toward characterizing extracellular vesicles at a single-particle level. J. Biomed. Sci..

[87] Jung A.L., Møller Jørgensen M., Bæk R., Griss K., Han M., Auf Dem Brinke K., Timmesfeld N., Bertrams W., Greulich T., Koczulla R. (2020). Surface proteome of plasma extracellular vesicles as biomarkers for pneumonia and acute exacerbation of chronic obstructive pulmonary disease. J. Infect. Dis..

[88] Shao S., Fang H., Li Q., Wang G. (2020). Extracellular vesicles in inflammatory skin disorders: From pathophysiology to treatment. Theranostics.

[89] Tsuno H., Arito M., Suematsu N., Sato T., Hashimoto A., Matsui T., Omoteyama K., Sato M., Okamoto K., Tohma S. (2018). A proteomic analysis of serum-derived exosomes in rheumatoid arthritis. BMC Rheumatol..

[90] Withrow J., Murphy C., Liu Y., Hunter M., Fulzele S., Hamrick M.W. (2016). Extracellular vesicles in the pathogenesis of rheumatoid arthritis and osteoarthritis. Arthritis Res. Ther..

[91] Andreu Z., Yáñez-Mó (2014). Tetraspanins in extracellular vesicle formation and function. Front. Immunol..

[92] Brosseau C., Colas L., Magnan A., Brouard S. (2018). CD9 Tetraspanin: A new pathway for the regulation of inflammation?. Front. Immunol..

[93] Chettimada S., Lorenz D.R., Misra V., Dillon S.T., Reeves R.K., Manickam C., Morgello S., Kirk G.D., Mehta S.H., Gabuzda D. (2018). Exosome markers associated with immune activation and oxidative stress in HIV patients on antiretroviral therapy. Sci. Rep..

[94] Di Meglio P., Villanova F., Nestle F.O. (2014). Psoriasis. Cold Spring Harb. Perspect. Med..

[95] Théry C., Witwer K.W., Aikawa E., Alcaraz M.J., Anderson J.D., Andriantsitohaina R., Antoniou A., Arab T., Archer F., Atkin-Smith G.K. (2018). Minimal information for studies of extracellular vesicles 2018 (MISEV2018): A position statement of the international society for extracellular vesicles and update of the MISEV2014 guidelines. J. Extracell. Vesicles.

[96] Midekessa G., Godakumara K., Ord J., Viil J., Lättekivi F., Dissanayake K., Kopanchuk S., Rinken A., Andronowska A., Bhattacharjee S. (2020). Zeta potential of extracellular vesicles: Toward understanding the attributes that determine colloidal stability. ACS Omega.

[97] Jørgensen M., Bæk R., Pedersen S., Søndergaard E.K.L., Kristensen S.R., Varming K. (2013). Extracellular vesicle (EV) Array: Microarray capturing of exosomes and other extracellular vesicles for multiplexed phenotyping. J. Extracell. Vesicles.

[98] Breakefield X.O., Das S., Gandhi R., Sood A.K., Balaj L., Filant J., Nejad P., Paul A., Simonson B., Srinivasan S. (2017). Isolation of exosomal RNA from serum or plasma using the Qiagen miRNeasy Micro kit. Protocol. Exch..

[99] Li X., Ben-Dov I.Z., Mauro M., Williams Z. (2015). Lowering the quantification limit of the QubitTM RNA HS Assay using RNA spike-in. BMC Molec. Biol..

[100] R Core Team (2020). R: A language and environment for statistical computing. R Found. Stat. Comput..

[101] Andrews S. (2010). FastQC: A Quality Control Tool for High Throughput Sequence Data. http://www.bioinformatics.babraham.ac.uk/projects/fastqc.

[102] Bolger A.M., Lohse M., Usadel B. (2014). Trimmomatic: A flexible trimmer for Illumina sequence data. Bioinformatics.

[103] Langmead B., Salzberg S.L. (2012). Fast gapped-read alignment with Bowtie 2. Nat. Methods.

[104] Harrow J., Frankish A., Gonzalez J.M., Tapanari E., Diekhans M., Kokocinski F., Aken B.L., Barrell D., Zadissa A., Searle S. (2012). GENCODE: The reference human genome annotation for The ENCODE Project. Genome Res..

[105] Sweeney B., The RNAcentral Consortium (2019). A.; Petrov, A.I.; Burkov, B.; Finn, R.D.; Bateman, A.; Szymanski, M.; Karlowski, W.M.; Gorodkin, J.; Seemann, S.E.; et al. RNAcentral: A hub of information for non-coding RNA sequences. Nucleic Acids Res..

[106] Anders S., Pyl P.T., Huber W. (2015). HTSeq—A Python framework to work with high-throughput sequencing data. Bioinformatics.

[107] Zhou X., Lindsay H., Robinson M.D. (2014). Robustly detecting differential expression in RNA sequencing data using observation weights. Nucleic Acids Res..

[108] Chen Y., Wang X. (2020). miRDB: An online database for prediction of functional microRNA targets. Nucleic Acids Res..

[109] Yu G., He Q.-Y. (2016). ReactomePA: An R/Bioconductor package for reactome pathway analysis and visualization. Mol. Biosyst..

[110] Fabregat A., Jupe S., Matthews L., Sidiropoulos K., Gillespie M., Garapati P., Haw R., Jassal B., Korninger F., May B. (2018). The reactome pathway knowledgebase. Nucleic Acids Res..

[111] Wickham H. (2016). ggplot2: Elegant Graphics for Data Analysis.

[112] Gu Z., Eils R., Schlesner M. (2016). Complex heatmaps reveal patterns and correlations in multidimensional genomic data. Bioinformatics.

[113] Meng Y., Asghari M., Aslan M.K., Yilmaz A., Mateescu B., Stavrakis S., deMello A.J. (2021). Microfluidics for extracellular vesicle separation and mimetic synthesis: Recent advances and future perspectives. Chem. Eng. J..

