# Supplementary material for: Profiling Blood Serum Extracellular Vesicles in Plaque Psoriasis and Psoriatic Arthritis Patients Reveals Potential Disease Biomarkers
Source: Int J Mol Sci. 2022 Apr 4;23(7):4005. doi: 10.3390/ijms23074005 (PMC9000144; doi:10.3390/ijms23074005)
Supplement: Supplementary file 1 [file ijms-23-04005-s001.zip › ijms-1621255-supplementary.pdf]

Supplementary materials

Profiling blood serum extracellular vesicles in plaque psoriasis and psoriatic arthritis patients reveals potential disease biomarkers

Freddy Lättekivi [1], Irina Guljavina [1], Getnet Midekessa [1,2] , Janeli Viil [3] , Paul R. Heath [4] , Rikke Bæk [5] , Malene Møller Jørgensen [5,6] Aneta Andronowska [7] , Kulli Kingo [8,9] and Alireza Fazeli [1,2,10,*]

[1] Department of Pathophysiology, Institute of Biomedicine and Translational Medicine, University of Tartu, Ravila St. 14b, 50411, Tartu, Estonia ; freddy.lattekivi@ut.ee (F.L.) ; irina.guljavina@ut.ee (I.G.) ; getnet.balcha@ut.ee (G.M.)
[2] Institute of Veterinary Medicine and Animal Sciences, Estonian University of Life Sciences, Kreutzwaldi 62, 51006, Tartu, Estonia ;
[3] Department of Pharmacology, Institute of Biomedicine and Translational Medicine, University of Tartu, Ravila St. 14b, 50411, Tartu, Estonia ; janeli.viil@ut.ee (J.V.)
[4] Sheffield Institute of Translational Neuroscience, University of Sheffield, S10 2HQ, UK ; p.heath@sheffield.ac.uk
[5] Department of Clinical Immunology, Aalborg University Hospital, Urbansgade 32-36, DK-9000, Aalborg, Denmark ; rikke.baek@rn.dk (R.B.) ; maljoe@rn.dk (M.M.J.)
[6] Department of Clinical Medicine, Aalborg University, Aalborg, Denmark ; maljoe@rn.dk
[7] Department of Hormonal Action Mechanisms, Institute of Animal Reproduction and Food Research, Polish Academy of Sciences, Olsztyn, Poland ; a.andronowska@pan.olsztyn.pl
[8] Clinic of Dermatology, Institute of Clinical Medicine, University of Tartu, Tartu, Estonia; kylli.kingo@ut.ee
[9] Clinic of Dermatology, Tartu University Hospital, Tartu, Estonia.; kylli.kingo@ut.ee
[10] Academic Unit of Reproductive and Developmental Medicine, Department of Oncology and Metabolism, Medical School, University of Sheffield, UK ; a.fazeli@sheffield.ac.uk
* Correspondence: fazeli@emu.ee; Tel.: +372-737-4425

Citation: Lättekivi, F.; Guljavina, I.; Midekessa, G.; Viil, J.; Heath, P.R.; Bæk, R.; Jørgensen, M.M.; Andronowska, A.; Kingo, K.; Fazeli, A. Profiling Blood Serum Extracellular Vesicles in Plaque Psoriasis and Psoriatic Arthritis Patients Reveals Potential Disease Biomarkers. *Int. J. Mol. Sci.* **2022**, *23*, 4005. https://doi.org/10.3390/ijms23074005

Academic Editors: Michal Zmijewski and Naoko Kanda

Received: 16 February 2022
Accepted: 29 March 2022
Published: 4 April 2022

Publisher's Note: MDPI stays neutral with regard to jurisdictional claims in published maps and institutional affiliations.

Copyright: © 2022 by the authors. Licensee MDPI, Basel, Switzerland. This article is an open access article distributed under the terms and conditions of the Creative Commons Attribution (CC BY) license (https://creativecommons.org/licenses/by/4.0/).

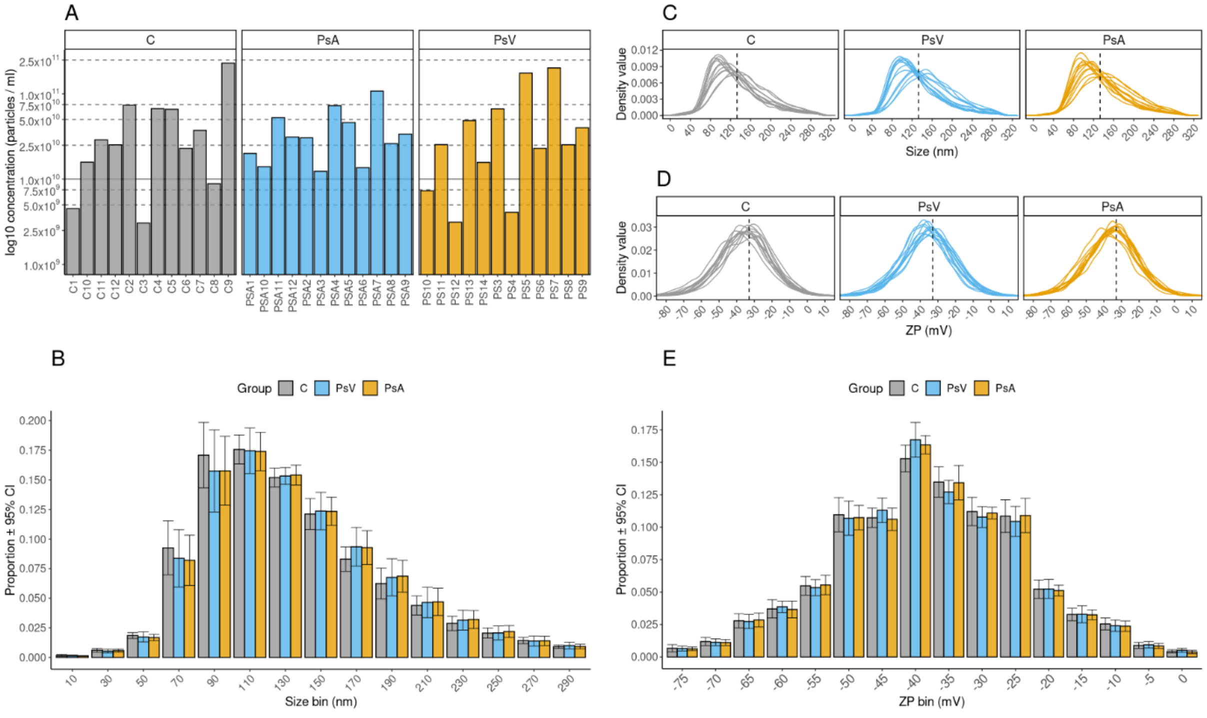

Figure S1: Nanoparticle size and zeta potential profile of EV samples. (A) Total concentration of nanoparticles in the EV samples purified from the blood serum of patients. Concentration is presented in log scale. **(B)** Size profile of nanoparticles in the EV samples represented as normalized concentration, i.e. total concentration in each size bin divided by the total concentration. Error bars are 95% confidence intervals. **(C)** Size distribution of nanoparticles in individual EV samples as density curves. Dashed vertical lines mark the average nanoparticle size across all samples. **(D)** Zeta potential profile of nanoparticles in individual EV samples as density curves. Dashed vertical lines mark the average zeta potential across all samples. **(E)** Zeta potential profile on nanoparticles in the EV samples represented as normalized concentration per millivolt (mV) range, i.e. total concentration in each range divided by the total concentration. Error bars are 95% confidence intervals.

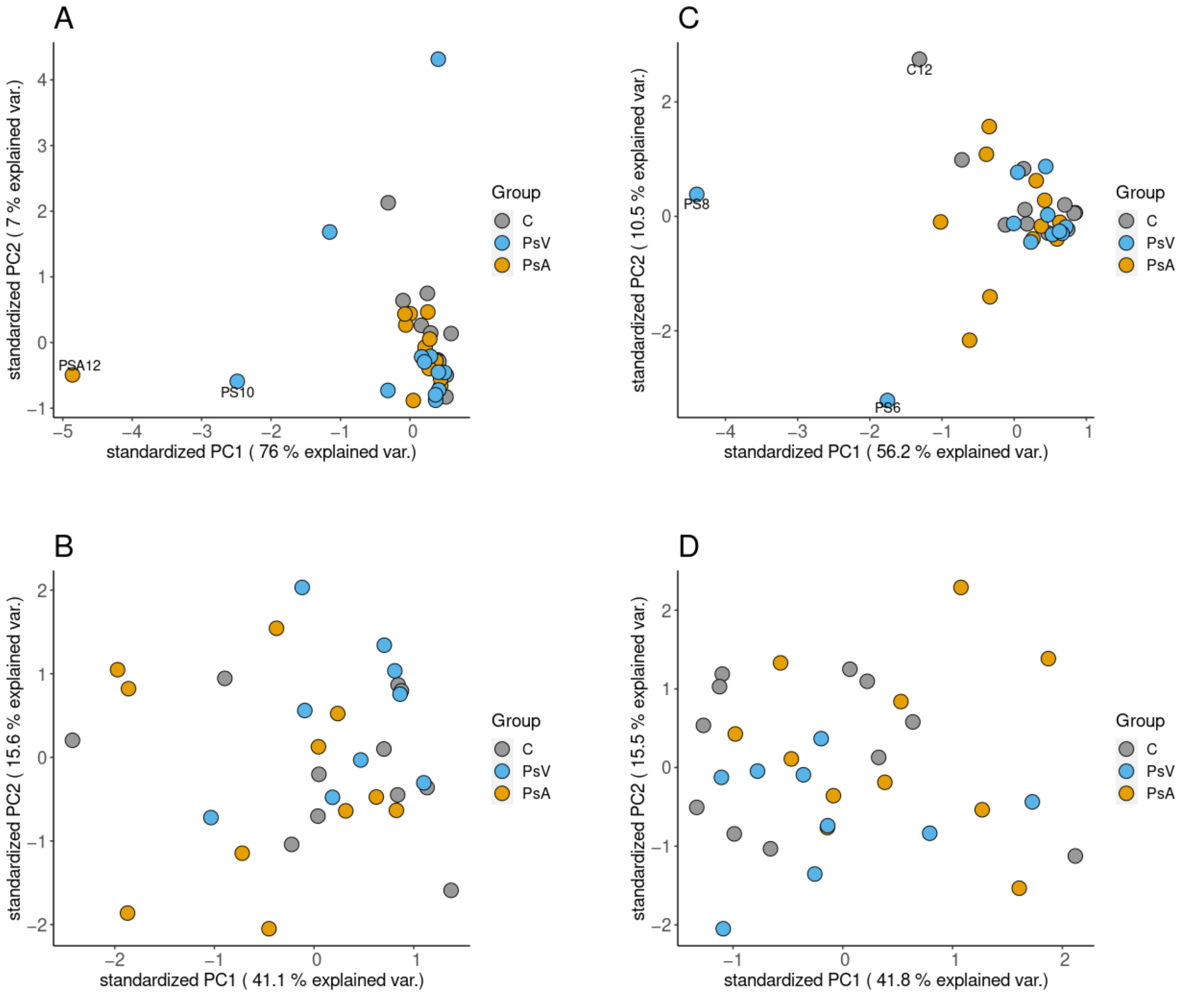

Figure S2: Outlier samples in the analysis of EV Array data. (A) The first two principal components calculated based on all 36 samples and 30 markers analyzed with the EV array. Two samples failed to be measured (PS10 and PSA12) are annotated. **(B)** The first two principal components calculated based on 34 samples with obtained measurements for 30 markers. Three visual outliers (PS6, PS8 and C12) are annotated. **(C)** The first two principal components calculated based on remaining 31 samples filtered for 13 markers considered to be uniformly detected. **(D)** The first two principal components calculated based on remaining 31 samples filtered for 13 markers with linear model residuals resulting from models with patient age and storage time of serum samples as predictors and abundance of the surface marker as the response variable. All values were standardized (z-score) prior to principal component calculations.

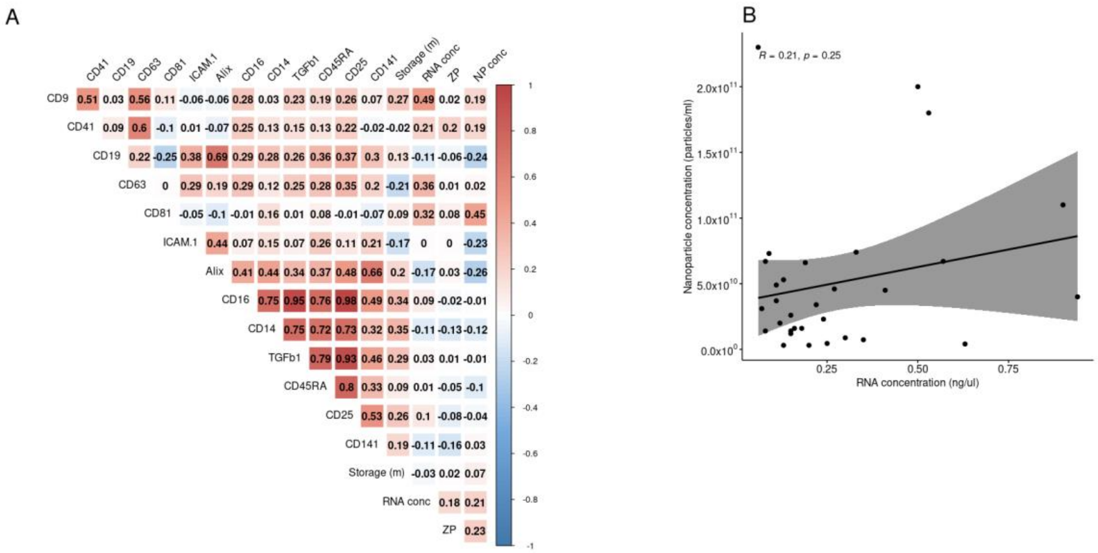

Figure S3: Correlations of EV characteristics, technical factors and abundance of measured EV surface protein markers. (A) Correlation matrix of 13 EV surface protein markers considered to be uniformly detected based on 31 samples, EV characteristics [ZP – zeta potential (mV), NP conc – nanoparticle concentration (particles/mL)], and technical factors [Storage (m) – serum sample storage time in months, RNA conc – concentration of isolated RNA (ng/μL)]. The displayed values are Pearson's correlation coefficients. **(B)** Correlation analysis of nanoparticle concentration (particles/mL) and RNA yield RNA (ng/μL) in all 36 purified EV samples.

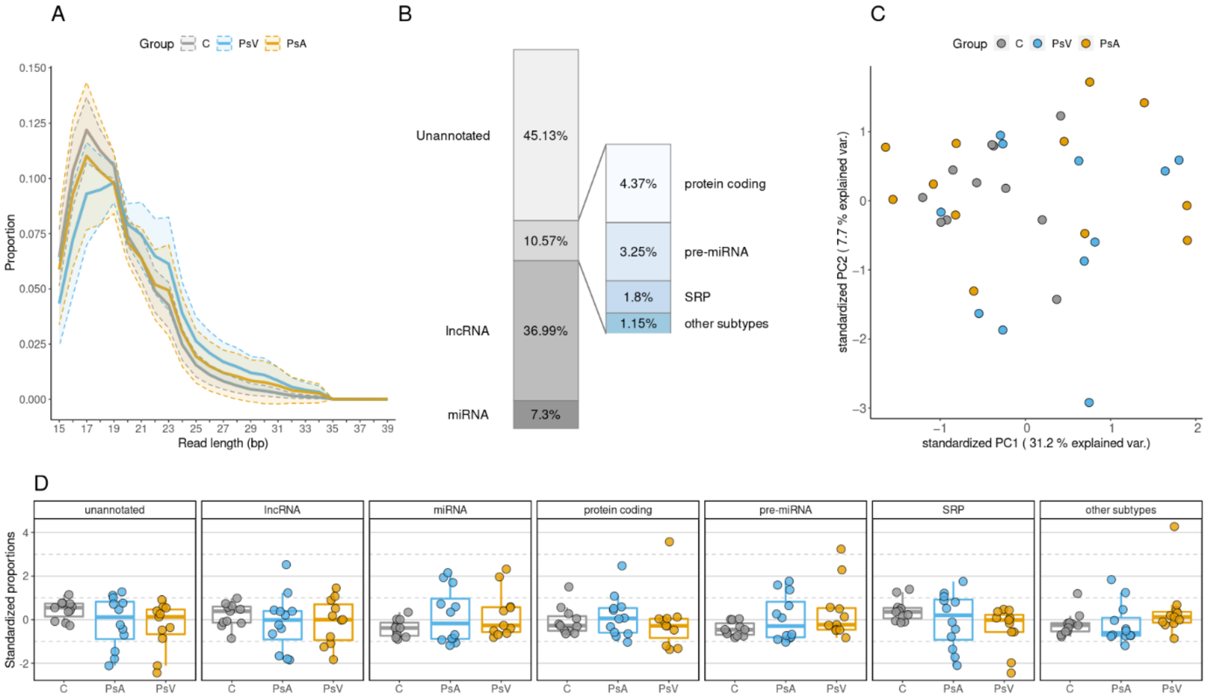

Figure S4: Small RNA profile of EV samples. (A) Length of sequenced small RNA molecules. Solid lines represent the average proportion of different read lengths in the three patient groups. Dashed lines represent the boundaries of 1 standard deviation around the mean. **(B)** Approximate proportions of different RNA subtypes among the small RNA molecules in the EV samples. **(C)** The first two principal components calculated based on the read counts of all annotated RNA sequences considered to be uniformly detected in at least one of the patient groups. **(D)** Standardized (z-score) proportions of different RNA subtypes at the individual sample level.

Table S1. Patient characteristics.

Sample ID	Group	Gender	Age	PASI	Main Diagnosis	Secondary Diagnoses
PS3	PsV	M	54	16.8	L40.0	
PS4	PsV	M	47	21.6	L40.0	
PS5	PsV	M	48	15.8	L40.0	
PS6	PsV	M	60	24.6	L40.0	
PS7	PsV	F	61	14.8	L40.0	
PS8	PsV	F	55	15.4	L40.0	
PS9	PsV	M	29	21.2	L40.0	
PS10	PsV	M	34	10.4	L40.0	
PS11	PsV	M	59	21.5	L40.0	
PS12	PsV	M	53	12.6	L40.0	
PS13	PsV	M	50	15.4	L40.0	
PS14	PsV	M	57	22.4	L40.0	
PSA1	PsA	M	34	22.6	L40.5	
PSA2	PsA	M	47	18.6	L40.0	L85.3; L40.5
PSA3	PsA	M	53	24.1	L40.5	
PSA4	PsA	M	49	12.7	L40.0	L40.5
PSA5	PsA	M	54	11.0	L40.5	I10
PSA6	PsA	M	29	23.1	L40.0	L40.5
PSA7	PsA	M	53	16.1	L40.0	L40.5; I11.9; F32.2
PSA8	PsA	F	61	20.3	L40.0	E11.8; L40.5; I11.9
PSA9	PsA	M	61	20.9	L40.0	L40.5
PSA10	PsA	F	60	21.6	L40.0	I11.0; K21.9; K29.4; L40.5; L55.1
PSA11	PsA	M	45	16.8	L40.0	L40.5
PSA12	PsA	M	55	24.8	L40.0	I11.9; L40.5
C1	C	M	24		L71.8	
C2	C	M	35		B86	L21.8; B35.1
C3	C	M	51		L73.2	
C4	C	M	57		L28.1	
C5	C	F	61		L73.8	L98.1
C6	C	M	64		L85.3	B35.6; Z03.8

C7	C	F	62	B86	E11.9; I10; L97
C8	C	M	49	L85.1	B35.1; B35.3
C9	C	M	52	L73.2	L21.8
C10	C	M	55	L85.8	D69.4
C11	C	M	54	B35.3	B35.1
C12	C	M	55	B86	

Group: PsV – *psoriasis vulgaris* group, PsA – *psoriasis vulgaris* + psoriatic arthritis group, C – noninflammatory control group.
Age: age of patients on the date of serum sampling.
Main Diagnosis: main diganosis assigned during the patient visit.
Seconday Diagnosis: secondary diagnoses assigned during patient visit.

Table S2. Antibodies used in EV Array phenotyping.

Target protein	Manufacturer	Catalog number
Alix	Biolegend, USA	634501
CD8a	R&D Systems, USA	MAB1509
CD9	Ancell Corporation, USA	156-020
CD13	R&D Systems, USA	MAB3815
CD14	R&D Systems, USA	MAB3833
CD16	BD Biosciences, USA	555404
CD19	R&D Systems, USA	MAB4867
CD25	BD Biosciences, USA	555430
CD41	Biolegend, USA	303702
CD42a	LSBio, USA	LS-C45240
CD42b	R&D Systems, USA	MAB4067
CD45	R&D Systems, USA	MAB1430
CD45RA	eBioscience, USA	14-0458-82
CD63	Bio-Rad	MCA2142
CD80	R&D Systems, USA	MAB140
CD81	Ancell Corporation, USA	302-020
CD83	R&D Systems, USA	MAB1774
CD141	R&D Systems, USA	MAB3947
CD146	Abcam, UK	ab24577
CD147	Abcam, UK	ab11572
EGFR	antibodies-online GmbH, Germany	ABIN191750
Flotillin-1	Abcam, UK	ab41927
Hsp90	Abcam, UK	ab13495

ICAM-1	eBioscience, USA	BMS1011
LFA1	Abbiotec, USA	250944
TGFβ1	BD Biosciences, USA	555052
THBS1	R&D Systems, USA	AF3074
TSG101	Abnova, Taiwan	H00007251-mol
VE-Cadherin	R&D Systems, USA	AF938
VEGFR2	Biolegend, USA	359902

Table S3. Results of miRNA differential enrichment testing between PsA and PsV group.

miRNA	log_2FC	log_2CPM	p-value	FDR
QXBT12	-1.11	10.61	0.00053	0.058
hsa-miR-33a-5p	0.87	9.15	0.000547	0.058
hsa-miR-26a-5p	0.44	16.17	0.00101	0.060
hsa-miR-mizuguchi-225	0.85	9.10	0.00113	0.060
miTC3	-1.21	7.14	0.00242	0.088
hsa-miR-338-5p	1.21	6.88	0.00273	0.088
hsa-miR-671-3p	-1.23	6.35	0.0029	0.088
hsa-miR-342-3p	-0.95	11.39	0.00375	0.099
hsa-miR-502-3p	-0.82	7.34	0.0053	0.125
hsa-miR-335-5p	-0.71	8.24	0.00851	0.151
hsa-miR-2355-5p	1.20	6.75	0.0086	0.151
hsa-miR-mizuguchi-223	0.39	14.88	0.00862	0.151
hsa-miR-21-5p	0.38	16.51	0.00977	0.151
hsa-miR-126-5p	0.46	15.24	0.00995	0.151
hsa-miR-203a-3p	-1.22	6.68	0.0114	0.158
hsa-miR-361-3p	-0.97	8.41	0.0119	0.158
hsa-miR-200c-3p	-0.88	7.63	0.015	0.186
hsa-miR-93-3p	-1.01	7.40	0.0162	0.188
hsa-miR-27a-3p	0.35	12.03	0.0194	0.188
hsa-miR-10b-5p	-0.69	9.75	0.0201	0.188
hsa-miR-423-5p	-0.58	12.47	0.0205	0.188
hsa-miR-342-5p	-0.75	7.94	0.0208	0.188
hsa-miR-27b-3p	0.42	12.90	0.021	0.188
hsa-miR-451a	-0.59	17.39	0.0213	0.188
hsa-miR-181b-5p	-1.02	6.77	0.0227	0.192
hsa-miR-339-3p	0.70	8.14	0.025	0.202
hsa-miR-92a-3p	-0.46	11.88	0.0257	0.202
miTC1	-0.79	12.27	0.0346	0.262
hsa-miR-134-5p	0.73	8.12	0.0421	0.295
hsa-miR-1301-3p	-0.66	7.26	0.0422	0.295
hsa-miR-320a	-0.43	12.59	0.0431	0.295

hsa-let-7d-5p	-0.49	9.71	0.0495	0.306
miTC8	-0.90	7.66	0.05	0.306
hsa-miR-532-5p	0.45	9.69	0.0513	0.306
hsa-miR-543	-0.80	7.49	0.0517	0.306
hsa-miR-374b-5p	-0.69	7.23	0.0522	0.306
hsa-miR-628-3p	0.43	8.01	0.0533	0.306
hsa-miR-486-3p	-0.78	6.77	0.0583	0.323
hsa-let-7e-5p	0.58	7.17	0.0615	0.323
hsa-miR-17-5p	-0.30	12.32	0.0618	0.323
hsa-mir-4433b precursor	-0.70	10.68	0.0625	0.323
hsa-miR-107	0.38	10.82	0.0702	0.354
hsa-miR-126-3p	0.31	15.51	0.0879	0.413
hsa-miR-20b-5p	-0.38	9.74	0.0907	0.413
hsa-miR-18a-3p	-0.70	6.51	0.0924	0.413
hsa-miR-151a-5p	0.43	7.86	0.0934	0.413
hsa-miR-181c-5p	0.44	7.78	0.0936	0.413
hsa-miR-345-5p	0.43	8.14	0.0972	0.420
hsa-miR-598-3p	0.36	9.38	0.105	0.437
hsa-miR-874-3p	-0.49	8.33	0.105	0.437
hsa-miR-93-5p	-0.26	15.59	0.109	0.437
hsa-miR-15b-5p	-0.49	12.18	0.109	0.437
Hsa-Mir-154-P3b_3p	0.62	9.06	0.115	0.444
hsa-miR-484	-0.26	13.64	0.116	0.444
hsa-miR-185-5p	-0.34	10.41	0.118	0.444
hsa-miR-532-3p	-0.61	7.30	0.119	0.444
hsa-miR-382-5p	0.54	8.87	0.121	0.444
hsa-miR-28-5p	0.30	9.66	0.128	0.459
hsa-miR-301a-3p	-0.44	9.63	0.13	0.459
hsa-miR-197-3p	-0.40	9.04	0.136	0.462
hsa-miR-22-5p	0.47	7.47	0.138	0.462
hsa-miR-369-3p	-0.64	7.13	0.142	0.462
hsa-miR-223-3p	-0.32	13.58	0.142	0.462
hsa-miR-181a-2-3p	0.43	8.49	0.143	0.462
hsa-miR-31-5p	-0.58	7.01	0.144	0.462
hsa-miR-505-3p	0.32	9.50	0.15	0.469
hsa-miR-485-3p	0.61	8.40	0.151	0.469
hsa-miR-23a-3p	0.21	14.51	0.155	0.475
hsa-miR-324-3p	-0.49	7.09	0.158	0.479
hsa-miR-224-5p	0.56	9.06	0.169	0.501
hsa-miR-629-5p	-0.50	6.95	0.17	0.501
hsa-miR-338-3p	-0.42	7.68	0.173	0.501
hsa-miR-409-3p	0.51	8.24	0.188	0.530
hsa-miR-411-5p	0.48	8.41	0.188	0.530
hsa-miR-21-3p	0.35	8.85	0.192	0.532

hsa-miR-30e-3p	-0.32	8.52	0.193	0.532
hsa-miR-210-3p	0.31	9.96	0.229	0.605
hsa-miR-100-5p	-0.48	8.40	0.229	0.605
hsa-miR-152-3p	0.32	8.19	0.231	0.605
hsa-miR-17-3p	0.27	9.36	0.231	0.605
hsa-miR-15b-3p	0.31	8.06	0.236	0.611
Hsa-Mir-28-P2_5p	0.24	9.70	0.247	0.627
hsa-miR-183-5p	-0.42	7.71	0.249	0.627
hsa-miR-6803-3p	-0.33	7.49	0.254	0.633
hsa-miR-181c-3p	0.41	7.07	0.26	0.635
hsa-miR-652-3p	-0.23	11.08	0.26	0.635
hsa-miR-20a-5p	-0.18	12.23	0.27	0.639
hsa-miR-29a-3p	-0.25	12.08	0.271	0.639
hsa-miR-29c-3p	-0.22	11.92	0.275	0.639
hsa-miR-10a-5p	-0.32	9.14	0.277	0.639
hsa-miR-127-3p	-0.40	8.06	0.28	0.639
hsa-miR-143-3p	0.26	9.99	0.281	0.639
hsa-miR-30a-3p	-0.36	7.74	0.289	0.639
hsa-let-7b-5p	-0.19	13.26	0.29	0.639
hsa-miR-146a-5p	0.25	11.89	0.292	0.639
hsa-miR-33b-5p	-0.43	7.30	0.293	0.639
hsa-miR-106a-5p	-0.30	8.35	0.298	0.642
hsa-let-7i-5p	0.20	14.17	0.3	0.642
hsa-miR-483-3p	-0.43	6.96	0.314	0.665
hsa-miR-425-3p	0.18	10.28	0.318	0.668
hsa-miR-140-3p	-0.18	12.66	0.323	0.671
hsa-miR-30a-5p	0.23	12.67	0.331	0.682
hsa-miR-181a-3p	0.31	8.48	0.34	0.687
Hsa-Mir-574_5p*	-0.34	7.54	0.342	0.687
hsa-miR-454-3p	-0.23	9.95	0.344	0.687
hsa-miR-487b-3p	0.31	9.02	0.348	0.689
miR-L-2	0.40	9.36	0.362	0.711
hsa-miR-150-3p	-0.33	8.24	0.37	0.719
hsa-miR-122-5p	-0.42	9.37	0.382	0.736
hsa-miR-24-2-5p	0.25	7.55	0.39	0.739
Hsa-Mir-154-P18_3p	0.30	7.52	0.393	0.739
hsa-miR-181d-5p	0.22	7.91	0.401	0.739
hsa-miR-26b-5p	0.12	13.96	0.401	0.739
hsa-miR-431-5p	0.38	8.06	0.408	0.739
hsa-miR-95-3p	0.33	7.42	0.41	0.739
hsa-miR-654-3p	0.37	8.52	0.411	0.739
hsa-miR-132-3p	0.18	9.32	0.411	0.739
hsa-miR-34a-5p	-0.31	7.93	0.416	0.742
hsa-miR-140-5p	0.24	8.20	0.421	0.744

hsa-miR-410-3p	-0.31	7.19	0.438	0.768
hsa-miR-mizuguchi-154	-0.29	7.26	0.455	0.785
hsa-miR-142-3p	-0.14	12.34	0.456	0.785
hsa-miR-23b-3p	0.12	13.21	0.461	0.785
hsa-miR-660-5p	0.18	10.01	0.464	0.785
hsa-miR-148a-3p	0.16	11.00	0.467	0.785
hsa-miR-1908-5p	-0.26	7.67	0.473	0.790
hsa-miR-186-5p	0.13	10.71	0.478	0.791
hsa-miR-101-3p	0.17	8.09	0.493	0.811
hsa-miR-432-5p	0.31	7.27	0.509	0.822
hsa-miR-196b-5p	0.16	8.72	0.511	0.822
hsa-miR-16-5p	0.18	8.95	0.512	0.822
hsa-miR-99b-3p	0.22	6.87	0.522	0.824
hsa-miR-142-5p	-0.10	12.27	0.522	0.824
hsa-miR-19a-3p	-0.10	12.48	0.529	0.824
hsa-miR-99b-5p	0.16	9.58	0.529	0.824
hsa-miR-150-5p	-0.17	14.42	0.539	0.826
hsa-miR-32-5p	-0.18	7.87	0.546	0.826
hsa-miR-584-5p	0.14	9.40	0.55	0.826
hsa-mir-6852 precursor	-0.21	7.09	0.55	0.826
hsa-miR-421	-0.17	8.59	0.552	0.826
hsa-miR-425-5p	-0.11	13.13	0.556	0.826
hsa-miR-15a-5p	0.10	13.98	0.559	0.826
hsa-miR-223-5p	-0.16	7.53	0.561	0.826
hsa-miR-324-5p	0.13	9.05	0.574	0.834
hsa-miR-204-5p	0.17	7.71	0.578	0.834
hsa-miR-139-3p	-0.21	7.68	0.592	0.834
hsa-miR-323a-3p	0.19	8.04	0.595	0.834
hsa-miR-423-3p	-0.13	11.11	0.596	0.834
hsa-miR-199b-5p	0.21	7.36	0.597	0.834
hsa-miR-136-3p	0.20	7.32	0.6	0.834
hsa-miR-101-5p	-0.18	7.37	0.6	0.834
hsa-miR-7-1-3p	-0.11	9.68	0.608	0.834
hsa-let-7f-5p	0.09	14.49	0.61	0.834
hsa-miR-144-3p	0.13	14.76	0.614	0.834
hsa-miR-195-5p	0.18	7.12	0.616	0.834
hsa-miR-1180-3p	-0.20	6.54	0.618	0.834
hsa-miR-98-5p	-0.09	10.62	0.63	0.845
hsa-miR-92b-3p	-0.11	9.76	0.636	0.847
hsa-miR-18a-5p	-0.09	9.60	0.639	0.847
hsa-miR-130b-3p	-0.10	9.85	0.644	0.847
hsa-miR-96-5p	-0.15	8.24	0.65	0.847
hsa-miR-22-3p	0.07	12.92	0.654	0.847
hsa-miR-139-5p	0.10	12.01	0.655	0.847

hsa-miR-222-3p	0.07	11.85	0.661	0.847
hsa-let-7g-5p	-0.09	13.32	0.664	0.847
hsa-miR-25-3p	0.09	14.42	0.67	0.847
hsa-miR-148b-3p	-0.08	9.88	0.674	0.847
hsa-miR-1306-5p	-0.10	8.24	0.675	0.847
hsa-miR-30b-5p	0.08	11.64	0.697	0.864
hsa-let-7a-5p	-0.07	13.12	0.704	0.864
hsa-miR-486-5p	-0.18	6.43	0.705	0.864
hsa-miR-376c-3p	-0.14	9.33	0.708	0.864
hsa-miR-3615	0.09	9.08	0.709	0.864
hsa-miR-296-5p	0.19	6.68	0.715	0.866
hsa-miR-155-5p	0.08	9.16	0.74	0.883
hsa-miR-199a-5p	0.06	11.78	0.749	0.883
hsa-miR-664a-5p	-0.13	6.51	0.752	0.883
hsa-miR-28-3p	0.05	10.88	0.755	0.883
hsa-miR-191-5p	-0.05	15.11	0.756	0.883
hsa-miR-363-3p	0.10	10.89	0.757	0.883
hsa-miR-744-5p	0.06	11.35	0.758	0.883
hsa-miR-362-3p	-0.11	6.79	0.777	0.900
hsa-miR-16-2-3p	0.07	10.56	0.789	0.905
hsa-let-7d-3p	0.08	8.65	0.79	0.905
hsa-miR-30d-5p	0.05	12.55	0.795	0.906
hsa-miR-130a-3p	-0.05	11.02	0.824	0.931
hsa-miR-106b-5p	0.05	9.84	0.826	0.931
hsa-miR-106b-3p	-0.04	10.94	0.851	0.952
hsa-miR-214-3p	-0.06	7.69	0.857	0.952
hsa-miR-128-3p	-0.07	7.63	0.863	0.952
hsa-miR-340-5p	0.05	8.33	0.866	0.952
hsa-miR-99a-5p	-0.04	11.27	0.874	0.952
hsa-miR-340-3p	0.05	7.75	0.876	0.952
hsa-miR-424-3p	-0.04	8.04	0.879	0.952
hsa-miR-2110	0.04	7.83	0.88	0.952
hsa-miR-30e-5p	0.03	11.56	0.889	0.956
hsa-miR-192-5p	-0.04	8.38	0.916	0.981
hsa-miR-328-3p	0.02	8.95	0.936	0.992
hsa-miR-145-5p	0.02	8.77	0.94	0.992
hsa-miR-766-3p	0.02	8.77	0.941	0.992
hsa-miR-1307-3p	0.01	11.29	0.953	0.993
hsa-miR-146b-5p	0.02	9.46	0.954	0.993
hsa-miR-361-5p	-0.01	9.80	0.96	0.993
hsa-miR-144-5p	-0.01	12.64	0.967	0.993
hsa-miR-330-3p	0.01	8.07	0.967	0.993
hsa-miR-497-5p	-0.01	9.63	0.974	0.993
hsa-miR-589-5p	0.02	6.96	0.978	0.993

hsa-miR-125a-5p	-0.01	11.87	0.98	0.993
hsa-miR-182-5p	0.01	9.52	0.984	0.993
hsa-miR-151a-3p	0.00	10.98	0.988	0.993
hsa-miR-221-3p	0.00	13.07	0.999	0.999

log_2FC – log_2 fold change of miRNA quantity between the two groups (contrast: PsA – PS). Log_2CPM – average log_2 counts per million of samples in both groups. FDR – false discovery rate.

Table S4. Results of miRNA differential enrichment testing between PsA and C group.

miRNA	log_2FC	log_2CPM	p-value	FDR
hsa-miR-10b-5p	-1.11	9.75	0.000204	0.043
hsa-miR-197-3p	-0.91	9.04	0.000873	0.081
hsa-miR-425-5p	0.59	13.13	0.00128	0.081
hsa-miR-199a-5p	0.65	11.78	0.00153	0.081
hsa-miR-203a-3p	-1.53	6.68	0.002	0.085
hsa-miR-10a-5p	-0.90	9.14	0.0025	0.088
hsa-miR-34a-5p	-1.13	7.93	0.00292	0.088
hsa-miR-532-3p	-1.17	7.30	0.00392	0.104
hsa-miR-340-3p	0.99	7.75	0.0045	0.106
hsa-miR-200c-3p	-1.06	7.63	0.00536	0.114
hsa-miR-99b-5p	-0.67	9.58	0.00699	0.135
hsa-miR-18a-5p	0.55	9.60	0.00812	0.143
hsa-miR-150-3p	-0.98	8.24	0.00894	0.146
hsa-miR-30d-5p	-0.45	12.55	0.0137	0.207
hsa-miR-30b-5p	-0.49	11.64	0.0155	0.218
hsa-miR-181d-5p	0.72	7.91	0.0182	0.218
hsa-miR-424-3p	0.73	8.04	0.0182	0.218
hsa-let-7d-5p	-0.59	9.71	0.0185	0.218
hsa-miR-409-3p	0.94	8.24	0.025	0.255
hsa-miR-107	0.49	10.82	0.0252	0.255
hsa-miR-150-5p	-0.62	14.42	0.0282	0.26
hsa-miR-15b-5p	-0.67	12.18	0.0283	0.26
hsa-miR-483-3p	-0.95	6.96	0.0304	0.269
hsa-miR-2355-5p	1.05	6.75	0.0342	0.286
hsa-miR-22-3p	0.34	12.92	0.035	0.286
hsa-miR-411-5p	0.79	8.41	0.0414	0.305
hsa-miR-93-3p	-0.89	7.40	0.042	0.305
hsa-miR-181c-5p	0.60	7.78	0.0421	0.305
hsa-miR-874-3p	-0.64	8.33	0.0431	0.305
hsa-miR-122-5p	-0.97	9.37	0.0462	0.309
miTC1	0.75	12.27	0.0467	0.309

hsa-miR-423-3p	0.50	11.11	0.0487	0.313
hsa-miR-423-5p	0.49	12.47	0.0517	0.322
hsa-miR-487b-3p	0.66	9.02	0.0581	0.352
hsa-let-7b-5p	-0.34	13.26	0.0667	0.393
hsa-miR-181b-5p	-0.87	6.77	0.0722	0.408
hsa-let-7g-5p	-0.38	13.32	0.0739	0.408
hsa-miR-6803-3p	0.63	7.49	0.075	0.408
hsa-miR-99b-3p	0.72	6.87	0.0782	0.415
hsa-miR-18a-3p	-0.78	6.51	0.0836	0.422
hsa-miR-151a-5p	0.49	7.86	0.0836	0.422
hsa-miR-20a-5p	0.28	12.23	0.09	0.429
hsa-miR-128-3p	0.71	7.63	0.0919	0.429
hsa-miR-660-5p	0.42	10.01	0.0926	0.429
hsa-miR-339-3p	0.55	8.14	0.093	0.429
hsa-miR-505-3p	0.39	9.50	0.0972	0.438
hsa-miR-195-5p	-0.61	7.12	0.1	0.441
hsa-miR-26a-5p	0.22	16.17	0.102	0.441
hsa-miR-328-3p	-0.46	8.95	0.107	0.442
hsa-miR-628-3p	0.39	8.01	0.111	0.442
hsa-miR-30e-5p	-0.30	11.56	0.114	0.442
hsa-miR-139-3p	-0.65	7.68	0.117	0.442
hsa-miR-29c-3p	0.33	11.92	0.118	0.442
hsa-miR-410-3p	0.73	7.19	0.118	0.442
hsa-let-7a-5p	-0.28	13.12	0.118	0.442
hsa-miR-335-5p	0.49	8.24	0.119	0.442
hsa-miR-196b-5p	0.40	8.72	0.121	0.442
hsa-miR-374b-5p	-0.59	7.23	0.13	0.467
hsa-miR-142-5p	0.24	12.27	0.147	0.513
hsa-miR-16-5p	0.43	8.95	0.148	0.513
hsa-miR-30a-5p	-0.34	12.67	0.15	0.513
hsa-miR-191-5p	0.23	15.11	0.157	0.527
hsa-miR-145-5p	0.45	8.77	0.162	0.538
hsa-miR-17-5p	0.23	12.32	0.166	0.541
hsa-miR-146a-5p	0.33	11.89	0.168	0.541
hsa-miR-664a-5p	-0.59	6.51	0.184	0.584
hsa-miR-532-5p	0.31	9.69	0.189	0.589
hsa-mir-6852 precursor	0.54	7.09	0.194	0.597
hsa-miR-mizuguchi-154	-0.52	7.26	0.199	0.602
hsa-let-7e-5p	0.43	7.17	0.204	0.61
hsa-miR-199b-5p	0.54	7.36	0.209	0.616
hsa-miR-342-3p	0.41	11.39	0.213	0.619
hsa-miR-25-3p	0.27	14.42	0.219	0.624
hsa-miR-130a-3p	0.27	11.02	0.221	0.624
hsa-miR-95-3p	-0.49	7.42	0.226	0.63

hsa-miR-382-5p	0.43	8.87	0.233	0.641
hsa-miR-185-5p	-0.26	10.41	0.246	0.668
hsa-miR-223-5p	0.37	7.53	0.249	0.669
hsa-miR-301a-3p	-0.33	9.63	0.277	0.731
hsa-miR-130b-3p	0.25	9.85	0.279	0.731
hsa-miR-106b-5p	-0.22	9.84	0.293	0.758
hsa-miR-369-3p	-0.48	7.13	0.301	0.769
hsa-miR-151a-3p	-0.24	10.98	0.31	0.78
hsa-miR-454-3p	0.25	9.95	0.316	0.78
hsa-miR-134-5p	0.37	8.12	0.316	0.78
hsa-miR-26b-5p	0.14	13.96	0.324	0.788
hsa-miR-221-3p	0.17	13.07	0.33	0.788
hsa-miR-155-5p	0.24	9.16	0.331	0.788
hsa-miR-29a-3p	-0.22	12.08	0.341	0.791
miTC8	-0.46	7.66	0.343	0.791
hsa-miR-324-5p	0.22	9.05	0.343	0.791
hsa-miR-7-1-3p	0.21	9.68	0.354	0.802
hsa-miR-432-5p	0.47	7.27	0.357	0.802
hsa-miR-20b-5p	0.22	9.74	0.36	0.802
hsa-miR-33b-5p	-0.39	7.30	0.365	0.802
hsa-miR-451a	0.23	17.39	0.367	0.802
hsa-miR-598-3p	0.20	9.38	0.374	0.81
hsa-let-7i-5p	0.17	14.17	0.382	0.819
hsa-miR-31-5p	-0.38	7.01	0.391	0.829
hsa-miR-27a-3p	-0.13	12.03	0.399	0.83
hsa-miR-1908-5p	0.34	7.67	0.402	0.83
hsa-miR-629-5p	-0.33	6.95	0.403	0.83
hsa-miR-214-3p	-0.29	7.69	0.413	0.84
hsa-miR-mizuguchi-225	0.22	9.10	0.416	0.84
hsa-miR-33a-5p	0.20	9.15	0.423	0.845
hsa-miR-497-5p	-0.21	9.63	0.428	0.848
hsa-miR-27b-3p	0.14	12.90	0.439	0.853
hsa-miR-182-5p	0.23	9.52	0.439	0.853
hsa-miR-30a-3p	-0.28	7.74	0.444	0.857
hsa-miR-1180-3p	-0.32	6.54	0.456	0.864
hsa-miR-766-3p	0.20	8.77	0.459	0.864
hsa-miR-1301-3p	-0.27	7.26	0.461	0.864
hsa-miR-183-5p	-0.29	7.71	0.465	0.864
hsa-miR-142-3p	-0.14	12.34	0.476	0.864
hsa-miR-24-2-5p	0.23	7.55	0.477	0.864
hsa-miR-338-5p	0.29	6.88	0.481	0.864
hsa-miR-652-3p	0.15	11.08	0.481	0.864
hsa-miR-98-5p	0.14	10.62	0.493	0.872
hsa-miR-28-3p	-0.11	10.88	0.495	0.872

hsa-miR-486-5p	0.36	6.43	0.497	0.872
hsa-miR-502-3p	-0.22	7.34	0.52	0.896
hsa-miR-148b-3p	-0.13	9.88	0.52	0.896
hsa-miR-3615	0.16	9.08	0.526	0.899
hsa-miR-376c-3p	0.23	9.33	0.548	0.922
hsa-miR-296-5p	-0.30	6.68	0.562	0.922
hsa-miR-148a-3p	-0.13	11.00	0.568	0.922
hsa-miR-584-5p	0.14	9.40	0.568	0.922
hsa-miR-106a-5p	0.17	8.35	0.574	0.922
hsa-miR-21-5p	0.08	16.51	0.581	0.922
hsa-miR-324-3p	-0.21	7.09	0.583	0.922
hsa-miR-mizuguchi-223	0.08	14.88	0.586	0.922
hsa-miR-139-5p	-0.12	12.01	0.587	0.922
hsa-miR-100-5p	0.23	8.40	0.59	0.922
hsa-miR-671-3p	-0.28	6.35	0.596	0.922
hsa-miR-15a-5p	0.09	13.98	0.598	0.922
hsa-miR-1307-3p	0.10	11.29	0.605	0.922
hsa-miR-361-5p	0.12	9.80	0.609	0.922
hsa-miR-543	-0.23	7.49	0.614	0.922
hsa-let-7f-5p	-0.09	14.49	0.623	0.922
hsa-miR-140-3p	0.09	12.66	0.626	0.922
hsa-miR-126-3p	0.09	15.51	0.627	0.922
hsa-miR-485-3p	0.21	8.40	0.63	0.922
hsa-miR-23b-3p	-0.08	13.21	0.631	0.922
hsa-miR-425-3p	0.09	10.28	0.633	0.922
hsa-miR-126-5p	0.08	15.24	0.637	0.922
hsa-miR-192-5p	-0.16	8.38	0.647	0.922
hsa-miR-16-2-3p	-0.12	10.56	0.651	0.922
hsa-miR-654-3p	-0.20	8.52	0.653	0.922
hsa-miR-96-5p	0.15	8.24	0.658	0.922
hsa-miR-32-5p	0.14	7.87	0.662	0.922
hsa-miR-320a	0.09	12.59	0.662	0.922
hsa-miR-22-5p	-0.14	7.47	0.669	0.922
miR-L-2	-0.19	9.36	0.67	0.922
hsa-miR-17-3p	-0.10	9.36	0.676	0.924
hsa-miR-589-5p	0.19	6.96	0.68	0.924
hsa-mir-4433b precursor	-0.15	10.68	0.687	0.927
hsa-miR-338-3p	-0.14	7.68	0.695	0.928
hsa-miR-362-3p	-0.16	6.79	0.697	0.928
hsa-miR-92a-3p	0.08	11.88	0.7	0.928
hsa-miR-99a-5p	-0.10	11.27	0.72	0.942
hsa-miR-101-3p	-0.09	8.09	0.726	0.942
Hsa-Mir-154-P18_3p	0.13	7.52	0.73	0.942
hsa-miR-144-3p	-0.08	14.76	0.744	0.942

hsa-miR-431-5p	0.15	8.06	0.745	0.942
hsa-miR-345-5p	0.09	8.14	0.747	0.942
hsa-miR-2110	-0.10	7.83	0.75	0.942
hsa-miR-127-3p	-0.12	8.06	0.756	0.942
hsa-miR-152-3p	0.08	8.19	0.759	0.942
hsa-miR-342-5p	-0.11	7.94	0.76	0.942
QXBT12	-0.10	10.61	0.762	0.942
hsa-miR-204-5p	-0.10	7.71	0.765	0.942
hsa-miR-323a-3p	0.11	8.04	0.769	0.943
hsa-miR-30e-3p	0.07	8.52	0.784	0.946
Hsa-Mir-28-P2_5p	0.06	9.70	0.789	0.946
hsa-miR-143-3p	-0.06	9.99	0.793	0.946
hsa-let-7d-3p	0.08	8.65	0.795	0.946
hsa-miR-125a-5p	-0.05	11.87	0.801	0.946
hsa-miR-181c-3p	0.10	7.07	0.802	0.946
hsa-miR-224-5p	0.10	9.06	0.807	0.946
hsa-miR-144-5p	-0.05	12.64	0.807	0.946
hsa-miR-421	0.07	8.59	0.816	0.95
miTC3	0.10	7.14	0.82	0.95
Hsa-Mir-154-P3b_3p	0.08	9.06	0.831	0.953
hsa-miR-23a-3p	-0.03	14.51	0.834	0.953
hsa-miR-330-3p	0.07	8.07	0.836	0.953
hsa-miR-101-5p	-0.07	7.37	0.849	0.963
hsa-miR-744-5p	-0.03	11.35	0.856	0.964
hsa-miR-140-5p	0.05	8.20	0.859	0.964
hsa-miR-28-5p	0.03	9.66	0.864	0.964
hsa-miR-186-5p	0.03	10.71	0.876	0.972
hsa-miR-106b-3p	-0.03	10.94	0.894	0.983
hsa-miR-340-5p	-0.04	8.33	0.895	0.983
hsa-miR-484	-0.02	13.64	0.912	0.985
hsa-miR-146b-5p	0.03	9.46	0.914	0.985
hsa-miR-1306-5p	-0.03	8.24	0.919	0.985
hsa-miR-21-3p	0.03	8.85	0.919	0.985
Hsa-Mir-574_5p*	-0.04	7.54	0.92	0.985
hsa-miR-210-3p	-0.02	9.96	0.93	0.988
hsa-miR-486-3p	0.04	6.77	0.932	0.988
hsa-miR-222-3p	-0.01	11.85	0.946	0.993
hsa-miR-93-5p	0.01	15.59	0.95	0.993
hsa-miR-223-3p	-0.01	13.58	0.951	0.993
hsa-miR-136-3p	-0.02	7.32	0.958	0.996
hsa-miR-92b-3p	0.01	9.76	0.971	0.997
hsa-miR-363-3p	0.01	10.89	0.974	0.997
hsa-miR-361-3p	0.01	8.41	0.981	0.997
hsa-miR-181a-2-3p	0.01	8.49	0.981	0.997

hsa-miR-181a-3p	-0.01	8.48	0.987	0.997
hsa-miR-132-3p	0.00	9.32	0.991	0.997
hsa-miR-15b-3p	0.00	8.06	0.997	0.997
hsa-miR-19a-3p	0.00	12.48	0.997	0.997

log_2FC – log_2 fold change of miRNA quantity between the two groups (contrast: PsA – C). Log_2CPM – average log_2 counts per million of samples in both groups. FDR – false discovery rate.

Table S5. Results of miRNA differential enrichment testing between PsV and C group.

miRNA	log_2FC	log_2CPM	p-value	FDR
hsa-miR-423-5p	1.07	12.47	0.0000358	0.0042
hsa-miR-335-5p	1.20	8.24	0.0000608	0.0042
hsa-miR-342-3p	1.36	11.39	0.0000652	0.0042
miTC1	1.54	12.27	0.0000791	0.0042
hsa-miR-425-5p	0.70	13.13	0.000183	0.0077
hsa-miR-99b-5p	-0.83	9.58	0.000898	0.0317
hsa-miR-17-5p	0.53	12.32	0.00148	0.0384
hsa-miR-18a-5p	0.64	9.60	0.00176	0.0384
hsa-miR-27a-3p	-0.48	12.03	0.0018	0.0384
hsa-miR-451a	0.83	17.39	0.00181	0.0384
QXBT12	1.01	10.61	0.00204	0.0394
miTC3	1.31	7.14	0.00279	0.0493
hsa-miR-6803-3p	0.96	7.49	0.00459	0.0697
hsa-miR-199a-5p	0.59	11.78	0.0046	0.0697
hsa-miR-30b-5p	-0.57	11.64	0.00554	0.0783
hsa-miR-20a-5p	0.46	12.23	0.00595	0.0789
hsa-miR-340-3p	0.95	7.75	0.00641	0.0799
hsa-miR-30d-5p	-0.50	12.55	0.00728	0.0858
hsa-miR-29c-3p	0.55	11.92	0.0092	0.1018
hsa-miR-424-3p	0.77	8.04	0.0106	0.1018
hsa-miR-92a-3p	0.54	11.88	0.0107	0.1018
hsa-miR-33a-5p	-0.66	9.15	0.0108	0.1018
hsa-miR-20b-5p	0.60	9.74	0.011	0.1018
hsa-miR-361-3p	0.98	8.41	0.0129	0.1135
hsa-miR-423-3p	0.63	11.11	0.0141	0.1195
hsa-miR-320a	0.53	12.59	0.0164	0.1336
hsa-miR-30a-5p	-0.58	12.67	0.0183	0.1409
hsa-miR-mizuguchi-225	-0.64	9.10	0.0186	0.1409
hsa-miR-410-3p	1.04	7.19	0.0242	0.1766
hsa-miR-34a-5p	-0.82	7.93	0.0262	0.1854
hsa-miR-195-5p	-0.79	7.12	0.0308	0.2105

hsa-miR-338-5p	-0.93	6.88	0.0349	0.231
hsa-miR-671-3p	0.95	6.35	0.0385	0.2406
hsa-miR-126-5p	-0.37	15.24	0.0386	0.2406
hsa-miR-142-5p	0.34	12.27	0.0397	0.2406
hsa-miR-mizuguchi-223	-0.31	14.88	0.0409	0.241
hsa-miR-95-3p	-0.82	7.42	0.0425	0.2434
hsa-miR-10a-5p	-0.58	9.14	0.0441	0.2461
hsa-miR-21-5p	-0.30	16.51	0.046	0.2502
hsa-miR-502-3p	0.60	7.34	0.0533	0.2815
hsa-miR-197-3p	-0.50	9.04	0.0554	0.2815
hsa-miR-454-3p	0.49	9.95	0.0558	0.2815
hsa-miR-342-5p	0.64	7.94	0.0571	0.2817
hsa-mir-6852 precursor	0.75	7.09	0.0608	0.2923
hsa-miR-486-3p	0.82	6.77	0.0629	0.2923
hsa-miR-128-3p	0.78	7.63	0.0634	0.2923
hsa-miR-22-5p	-0.61	7.47	0.0683	0.3079
hsa-miR-150-3p	-0.64	8.24	0.0713	0.3147
hsa-miR-652-3p	0.38	11.08	0.0727	0.3147
hsa-miR-328-3p	-0.48	8.95	0.0839	0.3544
hsa-miR-100-5p	0.71	8.40	0.0892	0.3544
hsa-miR-30e-5p	-0.33	11.56	0.0892	0.3544
hsa-miR-223-5p	0.54	7.53	0.09	0.3544
hsa-miR-191-5p	0.27	15.11	0.0903	0.3544
hsa-miR-22-3p	0.27	12.92	0.1	0.3819
hsa-miR-181d-5p	0.50	7.91	0.102	0.3819
hsa-miR-93-5p	0.27	15.59	0.103	0.3819
hsa-miR-26a-5p	-0.22	16.17	0.104	0.3819
hsa-miR-17-3p	-0.37	9.36	0.109	0.3881
hsa-miR-23a-3p	-0.25	14.51	0.11	0.3881
hsa-miR-106a-5p	0.47	8.35	0.118	0.4089
hsa-miR-1908-5p	0.60	7.67	0.121	0.4089
hsa-miR-150-5p	-0.45	14.42	0.122	0.4089
hsa-miR-130b-3p	0.35	9.85	0.124	0.4112
hsa-miR-30e-3p	0.39	8.52	0.129	0.4205
hsa-miR-532-3p	-0.56	7.30	0.132	0.4227
hsa-miR-27b-3p	-0.28	12.90	0.134	0.4231
hsa-miR-140-3p	0.27	12.66	0.148	0.4474
hsa-miR-10b-5p	-0.42	9.75	0.149	0.4474
hsa-miR-130a-3p	0.32	11.02	0.151	0.4474
hsa-miR-7-1-3p	0.32	9.68	0.154	0.4474
hsa-miR-484	0.24	13.64	0.154	0.4474
hsa-mir-4433b precursor	0.55	10.68	0.154	0.4474
hsa-miR-181a-2-3p	-0.43	8.49	0.16	0.4589
hsa-miR-223-3p	0.31	13.58	0.169	0.4782

hsa-miR-543	0.57	7.49	0.179	0.4881
hsa-miR-145-5p	0.43	8.77	0.181	0.4881
Hsa-Mir-154-P3b_3p	-0.54	9.06	0.182	0.4881
hsa-let-7g-5p	-0.29	13.32	0.184	0.4881
miR-L-2	-0.58	9.36	0.185	0.4881
hsa-miR-143-3p	-0.33	9.99	0.186	0.4881
hsa-miR-28-5p	-0.27	9.66	0.196	0.5055
hsa-miR-148a-3p	-0.29	11.00	0.201	0.5077
hsa-miR-106b-5p	-0.27	9.84	0.204	0.5077
hsa-miR-210-3p	-0.33	9.96	0.204	0.5077
hsa-miR-483-3p	-0.52	6.96	0.207	0.5077
hsa-miR-654-3p	-0.57	8.52	0.208	0.5077
hsa-miR-345-5p	-0.35	8.14	0.216	0.5202
hsa-miR-99b-3p	0.50	6.87	0.226	0.5389
hsa-miR-126-3p	-0.22	15.51	0.231	0.5408
hsa-miR-23b-3p	-0.20	13.21	0.232	0.5408
hsa-miR-21-3p	-0.32	8.85	0.241	0.5543
hsa-let-7a-5p	-0.21	13.12	0.245	0.5591
hsa-miR-98-5p	0.23	10.62	0.251	0.5661
hsa-miR-1301-3p	0.39	7.26	0.254	0.5664
hsa-miR-15b-3p	-0.31	8.06	0.263	0.5762
hsa-miR-122-5p	-0.54	9.37	0.265	0.5762
hsa-miR-224-5p	-0.46	9.06	0.266	0.5762
hsa-miR-664a-5p	-0.46	6.51	0.279	0.5957
hsa-miR-139-3p	-0.43	7.68	0.281	0.5957
hsa-miR-486-5p	0.54	6.43	0.302	0.6219
hsa-miR-32-5p	0.32	7.87	0.304	0.6219
hsa-miR-151a-3p	-0.25	10.98	0.305	0.6219
hsa-miR-101-3p	-0.27	8.09	0.31	0.6219
hsa-miR-409-3p	0.42	8.24	0.314	0.6219
hsa-miR-487b-3p	0.35	9.02	0.322	0.6219
hsa-miR-28-3p	-0.16	10.88	0.323	0.6219
hsa-let-7f-5p	-0.18	14.49	0.327	0.6219
hsa-miR-139-5p	-0.22	12.01	0.332	0.6219
hsa-miR-660-5p	0.24	10.01	0.333	0.6219
hsa-miR-221-3p	0.17	13.07	0.336	0.6219
hsa-miR-376c-3p	0.37	9.33	0.336	0.6219
hsa-miR-296-5p	-0.50	6.68	0.336	0.6219
miTC8	0.45	7.66	0.337	0.6219
hsa-miR-134-5p	-0.36	8.12	0.337	0.6219
hsa-miR-181a-3p	-0.32	8.48	0.342	0.6245
hsa-miR-196b-5p	0.24	8.72	0.346	0.6278
hsa-miR-485-3p	-0.41	8.40	0.357	0.6409
hsa-miR-96-5p	0.30	8.24	0.374	0.6662

hsa-miR-338-3p	0.28	7.68	0.383	0.6775
Hsa-Mir-28-P2_5p	-0.19	9.70	0.395	0.6915
hsa-miR-152-3p	-0.23	8.19	0.406	0.6992
hsa-miR-204-5p	-0.27	7.71	0.41	0.6992
hsa-miR-16-5p	0.24	8.95	0.412	0.6992
hsa-miR-144-3p	-0.21	14.76	0.416	0.6992
hsa-miR-421	0.24	8.59	0.421	0.6992
Hsa-Mir-574_5p*	0.30	7.54	0.427	0.6992
hsa-miR-132-3p	-0.18	9.32	0.427	0.6992
hsa-miR-25-3p	0.18	14.42	0.429	0.6992
hsa-miR-181c-3p	-0.32	7.07	0.432	0.6992
hsa-miR-411-5p	0.30	8.41	0.436	0.6992
hsa-miR-199b-5p	0.33	7.36	0.442	0.6992
hsa-miR-497-5p	-0.20	9.63	0.443	0.6992
hsa-let-7b-5p	-0.14	13.26	0.444	0.6992
hsa-miR-324-3p	0.28	7.09	0.445	0.6992
hsa-miR-182-5p	0.22	9.52	0.45	0.7011
hsa-miR-127-3p	0.28	8.06	0.453	0.7015
hsa-miR-16-2-3p	-0.18	10.56	0.478	0.7337
hsa-miR-203a-3p	-0.31	6.68	0.483	0.7368
hsa-miR-766-3p	0.18	8.77	0.499	0.7525
hsa-miR-598-3p	-0.16	9.38	0.504	0.7525
hsa-miR-155-5p	0.16	9.16	0.507	0.7525
hsa-miR-214-3p	-0.23	7.69	0.508	0.7525
hsa-miR-19a-3p	0.10	12.48	0.54	0.7922
hsa-miR-mizuguchi-154	-0.23	7.26	0.551	0.7922
hsa-miR-107	0.14	10.83	0.555	0.7922
hsa-miR-532-5p	-0.14	9.69	0.555	0.7922
hsa-miR-140-5p	-0.18	8.20	0.556	0.7922
hsa-miR-15b-5p	-0.18	12.18	0.557	0.7922
hsa-miR-361-5p	0.13	9.80	0.577	0.8148
hsa-miR-136-3p	-0.22	7.32	0.581	0.8161
hsa-miR-186-5p	-0.10	10.71	0.593	0.8252
hsa-miR-181c-5p	0.16	7.78	0.605	0.8252
hsa-miR-200c-3p	-0.18	7.63	0.606	0.8252
hsa-miR-31-5p	0.21	7.01	0.617	0.8252
hsa-miR-92b-3p	0.12	9.76	0.618	0.8252
hsa-miR-222-3p	-0.08	11.85	0.62	0.8252
hsa-miR-874-3p	-0.15	8.33	0.62	0.8252
hsa-miR-425-3p	-0.09	10.28	0.623	0.8252
hsa-miR-431-5p	-0.23	8.06	0.63	0.8252
hsa-miR-744-5p	-0.09	11.35	0.631	0.8252
hsa-miR-2110	-0.14	7.83	0.64	0.8308
Hsa-Mir-154-P18_3p	-0.17	7.52	0.646	0.8308

hsa-miR-1307-3p	0.09	11.29	0.647	0.8308
hsa-miR-339-3p	-0.15	8.14	0.66	0.8422
hsa-let-7d-5p	-0.11	9.71	0.666	0.8422
hsa-miR-629-5p	0.16	6.95	0.668	0.8422
hsa-let-7e-5p	-0.15	7.17	0.675	0.8422
hsa-miR-324-5p	0.10	9.05	0.675	0.8422
hsa-miR-589-5p	0.17	6.96	0.685	0.8497
hsa-miR-301a-3p	0.12	9.63	0.696	0.8581
hsa-miR-369-3p	0.16	7.13	0.708	0.8646
hsa-miR-185-5p	0.08	10.41	0.713	0.8646
hsa-miR-192-5p	-0.13	8.38	0.714	0.8646
hsa-miR-183-5p	0.14	7.71	0.718	0.8647
hsa-miR-181b-5p	0.14	6.77	0.739	0.8744
hsa-miR-146a-5p	0.08	11.89	0.744	0.8744
hsa-miR-382-5p	-0.11	8.87	0.754	0.8744
hsa-miR-432-5p	0.15	7.27	0.757	0.8744
hsa-miR-1180-3p	-0.12	6.54	0.763	0.8744
hsa-miR-340-5p	-0.09	8.33	0.764	0.8744
hsa-miR-101-5p	0.11	7.37	0.766	0.8744
hsa-miR-2355-5p	-0.15	6.75	0.772	0.8744
hsa-miR-374b-5p	0.10	7.23	0.773	0.8744
hsa-miR-93-3p	0.12	7.40	0.775	0.8744
hsa-miR-1306-5p	0.07	8.24	0.775	0.8744
hsa-miR-505-3p	0.07	9.50	0.776	0.8744
hsa-miR-3615	0.07	9.08	0.779	0.8744
hsa-miR-363-3p	-0.09	10.89	0.785	0.8763
hsa-miR-148b-3p	-0.05	9.88	0.81	0.8985
hsa-miR-125a-5p	-0.05	11.87	0.823	0.9021
hsa-miR-30a-3p	0.08	7.74	0.824	0.9021
hsa-miR-323a-3p	-0.08	8.04	0.826	0.9021
hsa-miR-151a-5p	0.06	7.86	0.833	0.904
hsa-miR-144-5p	-0.04	12.64	0.842	0.904
hsa-miR-99a-5p	-0.06	11.27	0.843	0.904
hsa-miR-18a-3p	-0.08	6.51	0.844	0.904
hsa-miR-330-3p	0.06	8.07	0.865	0.9212
hsa-let-7i-5p	-0.03	14.17	0.874	0.9264
hsa-miR-26b-5p	0.02	13.96	0.882	0.9266
hsa-miR-29a-3p	0.03	12.08	0.887	0.9266
hsa-miR-628-3p	-0.04	8.01	0.889	0.9266
hsa-miR-362-3p	-0.05	6.79	0.892	0.9266
hsa-miR-33b-5p	0.04	7.30	0.917	0.9487
hsa-miR-24-2-5p	-0.02	7.55	0.942	0.9694
hsa-miR-15a-5p	-0.01	13.98	0.956	0.9735
hsa-miR-106b-3p	0.01	10.94	0.959	0.9735

hsa-miR-146b-5p	0.01	9.46	0.96	0.9735
hsa-miR-142-3p	0.01	12.34	0.978	0.9873
hsa-miR-584-5p	0.00	9.40	0.996	0.9973
hsa-let-7d-3p	0.00	8.65	0.997	0.9973

log_2FC – log_2 fold change of miRNA quantity between the two groups (contrast: PS – C). Log_2CPM – average log_2 counts per million of samples in both groups. FDR – false discovery rate.

Table S6. Overrepresentation analysis of Reactome pathway annotations based on results of miRNA differential enrichment testing between PsA and PsV group.

Reactome ID	Description	Gene Ratio	Background Ratio	FDR	Genes
R-HSA-8986944	Transcriptional Regulation by MECP2	9/303	62/10654	0.047	PTEN; GRIA2; HIPK2; TRPC3; MEF2C; TNRC6C; TNRC6B; CAMK4; CREB1
R-HSA-400253	Circadian Clock	9/303	70/10654	0.047	MEF2C; SIRT1; ATF2; EP300; CPT1A; RORA; BHLHE40; CREB1; NR1D1
R-HSA-9006925	Intracellular signaling by second messengers	21/303	305/10654	0.047	PTEN; HGF; TNRC6C; GSK3B; TNRC6B; REST; COMMD3-BMI1; ATF2; RICTOR; PHC3; PRKCD; KPNA2; CAMK4; RING1; STRN; FGF18; USP13; CREB1; RHEB; PDGFRA; RCOR1
R-HSA-2559585	Oncogene Induced Senescence	6/303	33/10654	0.059	RB1; TNRC6C; ETS1; TNRC6B; CDK6; ID1
R-HSA-9018519	Estrogen-dependent gene expression	13/303	150/10654	0.059	STAG2; KCTD6; TNRC6C; TNRC6B; ATF2; CHD1; NR5A2; GTF2A1; KPNA2; EP300; KAT2B; H3-3B; KDM4B
R-HSA-1257604	PIP3 activates AKT signaling	18/303	264/10654	0.077	PTEN; HGF; TNRC6C; GSK3B; TNRC6B; REST; COMMD3-BMI1; ATF2; RICTOR; PHC3; RING1; STRN; FGF18; USP13; CREB1; RHEB; PDGFRA; RCOR1

Gene Ratio – ratio of genes associated with the pathway and total number of genes targeted by the subset of miRNAs.
Background Ratio – ratio of total number of genes associated with the pathway and total number of annotated genes. FDR – false discovery rate.
Genes – miRNA-targeted genes associated with the pathway.

Table S7. Overrepresentation analysis of Reactome pathway annotations based on results of miRNA differential enrichment testing between PsA and C group.

Reactome ID	Description	Gene Ratio	Background Ratio	FDR	Genes
R-HSA-212676	Dopamine Neurotransmitter Release Cycle	6/197	23/10654	0.002	CASK; SYT1; LIN7A; PPFIA1; VAMP2; LIN7C
R-HSA-6794362	Protein-protein interactions at synapses	8/197	87/10654	0.074	CASK; SYT1; IL1RAP; LIN7A; PPFIA1; FLOT2; LRRTM2; LIN7C
R-HSA-112310	Neurotransmitter release cycle	6/197	51/10654	0.083	CASK; SYT1; LIN7A; PPFIA1; VAMP2; LIN7C

Gene Ratio – ratio of genes associated with the pathway and total number of genes targeted by the subset of miRNAs.
Background Ratio – ratio of total number of genes associated with the pathway and total number of annotated genes. FDR – false discovery rate.
Genes – miRNA-targeted genes associated with the pathway.

Table S8. Overrepresentation analysis of Reactome pathway annotations based on results of miRNA differential enrichment testing between PsV and C group.

Reactome ID	Description	Gene Ratio	Background Ratio	FDR	Genes
R-HSA-983168	Antigen processing: Ubiquitination & Proteasome degradation	38/616	309/10654	0.005	FBXW7; UBE2V1; KCTD6; NEDD4L; UBE2Q2; KLHL2; FBXL20; UNKL; FBXL5; ASB3; FBXO32; SKP2; BTRC; FBXO31; HECTD2; NEDD4; TRIM36; CBLB; PSMD7; UBE2D2; UBE2G1; UBE2V2; SOCS1; SOCS3; NPEPPS; FBXL3; FBXO10; MKRN1; KLHL20; UBE3C; UBE2J1; ASB4; UBE2O; ZNRF1; KBTBD8; LONRF1; ASB6; TMEM189-UBE2V1

R-HSA-983169	Class I MHC mediated antigen processing & presentation	43/616	371/10654	0.005	FBXW7; UBE2V1; SAR1B; KCTD6; NEDD4L; UBE2Q2; KLHL2; FBXL20; UNKL; FBXL5; ASB3; FBXO32; SKP2; BTRC; FBXO31; HECTD2; NEDD4; TRIM36; CBLB; PSMD7; TAPBP; UBE2D2; UBE2G1; UBE2V2; SOCS1; SOCS3; NPEPPS; SEC23A; FBXL3; FBXO10; MKRN1; KLHL20; UBE3C; UBE2J1; ASB4; ERAP1; SEC24A; UBE2O; ZNRF1; KBTBD8; LONRF1; ASB6; TMEM189-UBE2V1
R-HSA-204998	Cell death signalling via NRAGE, NRIF and NADE	15/616	76/10654	0.009	PSEN1; MCF2L; VAV2; CASP2; ARHGEF26; GNA13; BEX3; FGD4; ARHGEF6; NGFR; TIAM1; SOS1; RASGRF2; SOS2; ARHGEF11
R-HSA-194840	Rho GTPase cycle	21/616	138/10654	0.009	MCF2L; RACGAP1; VAV2; ARHGAP26; ARHGAP32; SRGAP2; ARHGEF26; ARHGAP12; GNA13; TRIP10; FGD4; ARHGEF6; OCRL; TIAM1; RHOB; ARHGAP1; SOS1; RASGRF2; SOS2; ARHGEF11; ARHGAP21
R-HSA-4086398	Ca2+ pathway	13/616	62/10654	0.009	PPP3CA; PPP3R1; TNRC6B; FZD6; AGO1; CALM3; GNG5; GNB5; FZD4; TNRC6A; FZD3; AGO3; PLCB1
R-HSA-193648	NRAGE signals death through JNK	12/616	59/10654	0.021	MCF2L; VAV2; ARHGEF26; GNA13; FGD4; ARHGEF6; NGFR; TIAM1; SOS1; RASGRF2; SOS2; ARHGEF11
R-HSA-416482	G alpha (12/13) signalling events	14/616	80/10654	0.027	MCF2L; VAV2; ARHGEF26; GNA13; FGD4; ARHGEF6; TIAM1; RHOB; GNG5; SOS1; GNB5; RASGRF2; SOS2; ARHGEF11
R-HSA-8934593	Regulation of RUNX1 Expression and Activity	6/616	17/10654	0.033	RUNX1; TNRC6B; AGO1; TNRC6A; AGO3; CCND1
R-HSA-8856828	Clathrin-mediated endocytosis	20/616	146/10654	0.033	LDLR; CHRM2; AP2B1; SNX18; REPS1; AGFG1; FCHO2; M6PR; DAB2; RAB5B; TRIP10; RAB5A; OCRL; HBEGF; EGFR; ITSN2;

					FZD4; NECAP1; LDLRAP1; HSPA8
R-HSA-193704	p75 NTR receptor-mediated signalling	15/616	97/10654	0.041	PSEN1; MCF2L; VAV2; CASP2; ARHGEF26; GNA13; BEX3; FGD4; ARHGEF6; NGFR; TIAM1; SOS1; RASGRF2; SOS2; ARHGEF11
R-HSA-6794362	Protein-protein interactions at synapses	14/616	87/10654	0.041	GRIA3; GRIA4; GRIN2D; GRM5; NRXN1; APBA1; DLGAP1; EPB41L1; SLITRK1; SLITRK3; SHANK1; IL1RAPL2; LIN7C; LRFN2
R-HSA-9022692	Regulation of MECP2 expression and activity	8/616	33/10654	0.041	TNRC6B; CAMK2D; AGO1; CALM3; FOXG1; TNRC6A; AGO3; CREB1
R-HSA-112316	Neuronal System	40/616	413/10654	0.074	KCNA1; GABRB1; GLRB; GRIA3; GRIA4; GRIN2D; GRM5; KCNK2; AP2B1; NRXN1; NPTN; APBA1; GNAI2; LRTOMT; CAMK2D; DLGAP1; EPB41L1; SLITRK1; SLITRK3; SNAP25; KCNA4; MAPK1; SLC6A1; KCNB1; CALM3; GNG5; CACNG2; PRKAA2; GNB5; RASGRF2; SHANK1; IL1RAPL2; LIN7C; SLC17A7; LRFN2; KCNK10; CREB1; HSPA8; CAMKK2; PLCB1

Gene Ratio – ratio of genes associated with the pathway and total number of genes targeted by the subset of miRNAs.
Background Ratio – ratio of total number of genes associated with the pathway and total number of annotated genes. FDR – false discovery rate.
Genes – miRNA-targeted genes associated with the pathway.

Table S9. miRDB-derived predicted targets (score ≥ 90) of each miRNA used for the pathway-level enrichment analysis for PsA and PsV comparison.

miRNA	Gene	Entrez ID	RefSeq ID	miRDB Target Score
hsa-miR-33a-5p	ABCA1	19	NM_005502	100.0
hsa-miR-33a-5p	CROT	54677	NM_001143935	99.0
hsa-miR-33a-5p	CDK6	1021	NM_001259	99.0
hsa-miR-33a-5p	HMGA2	8091	NM_003483	98.8
hsa-miR-33a-5p	YWHAH	7533	NM_003405	98.6
hsa-miR-33a-5p	CADM2	253559	NM_001167674	98.3

hsa-miR-33a-5p	RORA	6095	NM_002943	97.5
hsa-miR-33a-5p	HADHB	3032	NM_000183	96.9
hsa-miR-33a-5p	DYNC1LI2	1783	NM_001286157	96.6
hsa-miR-33a-5p	HBS1L	10767	NM_001145158	96.2
hsa-miR-33a-5p	CPEB2	132864	NM_001177381	95.9
hsa-miR-33a-5p	GLCCI1	113263	NM_138426	95.3
hsa-miR-33a-5p	ABHD2	11057	NM_007011	95.3
hsa-miR-33a-5p	GRM8	2918	NM_001127323	94.9
hsa-miR-33a-5p	MTRNR2L3	100462983	NM_001190472	94.8
hsa-miR-33a-5p	CAMK4	814	NM_001323374	94.7
hsa-miR-33a-5p	PNMA1	9240	NM_006029	94.5
hsa-miR-33a-5p	PIM1	5292	NM_001243186	94.4
hsa-miR-33a-5p	GRIK2	2898	NM_175768	94.3
hsa-miR-33a-5p	ZNF140	7699	NM_001300776	93.9
hsa-miR-33a-5p	ZNF281	23528	NM_001281293	93.8
hsa-miR-33a-5p	RAP2A	5911	NM_021033	93.3
hsa-miR-33a-5p	NAA15	80155	NM_057175	93.3
hsa-miR-33a-5p	PHACTR2	9749	NM_001100164	93.2
hsa-miR-33a-5p	CLSPN	63967	NM_001190481	93.2
hsa-miR-33a-5p	ARID5B	84159	NM_001244638	93.1
hsa-miR-33a-5p	TTC28	23331	NM_001145418	93.1
hsa-miR-33a-5p	KCNMA1	3778	NM_001014797	93.1
hsa-miR-33a-5p	ARMC8	25852	NM_014154	92.7
hsa-miR-33a-5p	SCN8A	6334	NM_001177984	92.4
hsa-miR-33a-5p	SLC12A5	57468	NM_001134771	92.2
hsa-miR-33a-5p	SAMD8	142891	NM_144660	92.0
hsa-miR-33a-5p	CDK16	5127	NM_033018	91.9
hsa-miR-33a-5p	ANKRD44	91526	NM_001195144	91.4
hsa-miR-33a-5p	NAT8	9027	NM_003960	91.2
hsa-miR-33a-5p	VCAN	1462	NM_001126336	90.8
hsa-miR-33a-5p	HIPK2	28996	NM_001113239	90.7
hsa-miR-33a-5p	CPT1A	1374	NM_001876	90.6
hsa-miR-33a-5p	PDGFRA	5156	NM_006206	90.6
hsa-miR-33a-5p	ERBIN	55914	NM_001006600	90.6
hsa-miR-33a-5p	DENND1B	163486	NM_001195215	90.6
hsa-miR-33a-5p	DSC3	1825	NM_001941	90.5
hsa-miR-33a-5p	B3GALT2	8707	NM_003783	90.3
hsa-miR-33a-5p	MEGF10	84466	NM_001256545	90.3
hsa-miR-26a-5p	STRADB	55437	NM_001206864	99.8
hsa-miR-26a-5p	FAM98A	25940	NM_001304538	99.8
hsa-miR-26a-5p	CDK8	1024	NM_001260	99.7
hsa-miR-26a-5p	TET2	54790	NM_001127208	99.7
hsa-miR-26a-5p	CASZ1	54897	NM_001079843	99.6
hsa-miR-26a-5p	RHOQ	23433	NM_012249	99.4
hsa-miR-26a-5p	KLHL42	57542	NM_020782	99.3
hsa-miR-26a-5p	POLR3G	10622	NM_006467	99.3
hsa-miR-26a-5p	USP9X	8239	NM_001039590	99.2
hsa-miR-26a-5p	RPS6KA6	27330	NM_014496	99.2
hsa-miR-26a-5p	OSBPL11	114885	NM_022776	99.1
hsa-miR-26a-5p	NAP1L5	266812	NM_153757	99.0

hsa-miR-26a-5p	SLC2A13	114134	NM_052885	99.0
hsa-miR-26a-5p	TET1	80312	NM_030625	98.8
hsa-miR-26a-5p	ETNK1	55500	NM_018638	98.6
hsa-miR-26a-5p	CHORDC1	26973	NM_001144073	98.5
hsa-miR-26a-5p	ULK2	9706	NM_014683	98.5
hsa-miR-26a-5p	KCNJ2	3759	NM_000891	98.5
hsa-miR-26a-5p	FAM136A	84908	NM_032822	98.3
hsa-miR-26a-5p	STRBP	55342	NM_001171137	98.3
hsa-miR-26a-5p	TET3	200424	NM_001287491	98.3
hsa-miR-26a-5p	CLASP2	23122	NM_001207044	98.2
hsa-miR-26a-5p	PITPNC1	26207	NM_012417	98.2
hsa-miR-26a-5p	LSM12	124801	NM_152344	98.2
hsa-miR-26a-5p	LARP1	23367	NM_015315	98.2
hsa-miR-26a-5p	SLC25A16	8034	NM_001324312	98.1
hsa-miR-26a-5p	PEX13	5194	NM_002618	97.9
hsa-miR-26a-5p	BLOC1S2	282991	NM_001001342	97.8
hsa-miR-26a-5p	CEP350	9857	NM_014810	97.8
hsa-miR-26a-5p	PLOD2	5352	NM_000935	97.7
hsa-miR-26a-5p	ZSWIM6	57688	NM_020928	97.7
hsa-miR-26a-5p	SULF1	23213	NM_001128204	97.5
hsa-miR-26a-5p	FBXO28	23219	NM_001136115	97.4
hsa-miR-26a-5p	FRMD4B	23150	NM_015123	97.3
hsa-miR-26a-5p	PTEN	5728	NM_000314	97.3
hsa-miR-26a-5p	BAG4	9530	NM_001204878	97.2
hsa-miR-26a-5p	SYT10	341359	NM_198992	97.2
hsa-miR-26a-5p	PARPBP	55010	NM_001319996	97.0
hsa-miR-26a-5p	NHS	4810	NM_001136024	97.0
hsa-miR-26a-5p	USP3	9960	NM_001256702	97.0
hsa-miR-26a-5p	TNRC6B	23112	NM_001162501	96.9
hsa-miR-26a-5p	FBXL19	54620	NM_001099784	96.9
hsa-miR-26a-5p	COL10A1	1300	NM_000493	96.8
hsa-miR-26a-5p	TMC7	79905	NM_001160364	96.8
hsa-miR-26a-5p	EPC2	26122	NM_015630	96.8
hsa-miR-26a-5p	PHF3	23469	NM_001290259	96.6
hsa-miR-26a-5p	MTM1	4534	NM_000252	96.6
hsa-miR-26a-5p	GSK3B	2932	NM_001146156	96.5
hsa-miR-26a-5p	MSMO1	6307	NM_001017369	96.4
hsa-miR-26a-5p	UBR3	130507	NM_172070	96.4
hsa-miR-26a-5p	CAMSAP1	157922	NM_015447	96.3
hsa-miR-26a-5p	GNA13	10672	NM_006572	96.3
hsa-miR-26a-5p	LOXL2	4017	NM_002318	96.3
hsa-miR-26a-5p	ADAM17	6868	NM_003183	96.3
hsa-miR-26a-5p	CILP	8483	NM_003613	96.3
hsa-miR-26a-5p	PFKFB2	5208	NM_006212	96.3
hsa-miR-26a-5p	ALDH5A1	7915	NM_001080	96.1
hsa-miR-26a-5p	PRKCD	5580	NM_001316327	96.1
hsa-miR-26a-5p	NUS1	116150	NM_138459	95.8
hsa-miR-26a-5p	ULK1	8408	NM_003565	95.8
hsa-miR-26a-5p	CHSY1	22856	NM_014918	95.8
hsa-miR-26a-5p	ZDHHC6	64429	NM_001303134	95.7

hsa-miR-26a-5p	CREBZF	58487	NM_001039618	95.6
hsa-miR-26a-5p	MAP7	9053	NM_001198608	95.6
hsa-miR-26a-5p	PTGS2	5743	NM_000963	95.5
hsa-miR-26a-5p	TNRC6C	57690	NM_001142640	95.3
hsa-miR-26a-5p	MAPK6	5597	NM_002748	95.3
hsa-miR-26a-5p	BHLHE40	8553	NM_003670	95.2
hsa-miR-26a-5p	ATP11C	286410	NM_001010986	95.2
hsa-miR-26a-5p	VANGL2	57216	NM_020335	95.2
hsa-miR-26a-5p	NLK	51701	NM_016231	95.2
hsa-miR-26a-5p	DMRT3	58524	NM_021240	95.1
hsa-miR-26a-5p	ZIC5	85416	NM_033132	95.1
hsa-miR-26a-5p	PLCB1	23236	NM_015192	95.0
hsa-miR-26a-5p	NUP50	10762	NM_007172	95.0
hsa-miR-26a-5p	IQCJ	654502	NM_001042705	94.8
hsa-miR-26a-5p	SLC35E4	339665	NM_001318371	94.8
hsa-miR-26a-5p	UBE4B	10277	NM_001105562	94.7
hsa-miR-26a-5p	MPP6	51678	NM_001303037	94.6
hsa-miR-26a-5p	ZNF410	57862	NM_001242924	94.6
hsa-miR-26a-5p	JAG1	182	NM_000214	94.6
hsa-miR-26a-5p	RSPRY1	89970	NM_001305163	94.6
hsa-miR-26a-5p	KPNA2	3838	NM_001320611	94.6
hsa-miR-26a-5p	RCN2	5955	NM_001271837	94.4
hsa-miR-26a-5p	FBXO11	80204	NM_001190274	94.4
hsa-miR-26a-5p	INHBB	3625	NM_002193	94.4
hsa-miR-26a-5p	NA	NA	NM_001322103	94.1
hsa-miR-26a-5p	EPB41L3	23136	NM_001281533	94.1
hsa-miR-26a-5p	SLC16A6	9120	NM_001174166	94.0
hsa-miR-26a-5p	SFPQ	6421	NM_005066	94.0
hsa-miR-26a-5p	SLC45A4	57210	NM_001080431	93.9
hsa-miR-26a-5p	ART3	419	NM_001130016	93.9
hsa-miR-26a-5p	ERC2	26059	NM_015576	93.9
hsa-miR-26a-5p	GMDS	2762	NM_001253846	93.9
hsa-miR-26a-5p	CD200	4345	NM_001004196	93.9
hsa-miR-26a-5p	MTFMT	123263	NM_139242	93.8
hsa-miR-26a-5p	EIF2S1	1965	NM_004094	93.8
hsa-miR-26a-5p	RAB5IF	55969	NM_001199534	93.8
hsa-miR-26a-5p	FAM160A1	729830	NM_001109977	93.8
hsa-miR-26a-5p	SNN	8303	NM_003498	93.7
hsa-miR-26a-5p	SLC38A2	54407	NM_001307936	93.7
hsa-miR-26a-5p	PLEKHG1	57480	NM_001029884	93.6
hsa-miR-26a-5p	SRCAP	10847	NM_006662	93.6
hsa-miR-26a-5p	BCR	613	NM_004327	93.6
hsa-miR-26a-5p	LNX2	222484	NM_153371	93.6
hsa-miR-26a-5p	ABHD5	51099	NM_016006	93.6
hsa-miR-26a-5p	EP300	2033	NM_001429	93.5
hsa-miR-26a-5p	PFKFB3	5209	NM_001314063	93.5
hsa-miR-26a-5p	PAK2	5062	NM_002577	93.5
hsa-miR-26a-5p	YPEL1	29799	NM_013313	93.5
hsa-miR-26a-5p	PALM3	342979	NM_001145028	93.5
hsa-miR-26a-5p	ANKS1A	23294	NM_015245	93.5

hsa-miR-26a-5p	PHF20L1	51105	NM_001277196	93.4
hsa-miR-26a-5p	RCOR1	23186	NM_015156	93.3
hsa-miR-26a-5p	SLC5A1	6523	NM_000343	93.3
hsa-miR-26a-5p	G3BP2	9908	NM_012297	93.3
hsa-miR-26a-5p	CHFR	55743	NM_001161345	93.3
hsa-miR-26a-5p	EIF5	1983	NM_001969	93.2
hsa-miR-26a-5p	GRHL3	57822	NM_001195010	93.2
hsa-miR-26a-5p	RNF6	6049	NM_005977	93.1
hsa-miR-26a-5p	PPP1R15B	84919	NM_032833	93.1
hsa-miR-26a-5p	ARPP19	10776	NM_001306191	93.1
hsa-miR-26a-5p	TAF9B	51616	NM_015975	93.1
hsa-miR-26a-5p	MTX2	10651	NM_001006635	93.1
hsa-miR-26a-5p	DCDC2	51473	NM_001195610	93.1
hsa-miR-26a-5p	ABCC4	10257	NM_005845	93.0
hsa-miR-26a-5p	COL19A1	1310	NM_001858	93.0
hsa-miR-26a-5p	HTR1B	3351	NM_000863	92.8
hsa-miR-26a-5p	CELSR1	9620	NM_014246	92.8
hsa-miR-26a-5p	TRPC3	7222	NM_001130698	92.7
hsa-miR-26a-5p	ZBTB18	10472	NM_001278196	92.7
hsa-miR-26a-5p	RHD	6007	NM_001127691	92.7
hsa-miR-26a-5p	CTXN3	613212	NM_001048252	92.7
hsa-miR-26a-5p	SLC25A36	55186	NM_018155	92.7
hsa-miR-26a-5p	REST	5978	NM_001193508	92.6
hsa-miR-26a-5p	RBM24	221662	NM_001143941	92.6
hsa-miR-26a-5p	GMFB	2764	NM_004124	92.6
hsa-miR-26a-5p	SLC19A2	10560	NM_001319667	92.5
hsa-miR-26a-5p	DNA2	1763	NM_001080449	92.4
hsa-miR-26a-5p	TP53INP1	94241	NM_033285	92.4
hsa-miR-26a-5p	ITGB8	3696	NM_002214	92.4
hsa-miR-26a-5p	DAPK1	1612	NM_001288729	92.3
hsa-miR-26a-5p	KPNA6	23633	NM_012316	92.3
hsa-miR-26a-5p	CTTNBP2NL	55917	NM_018704	92.3
hsa-miR-26a-5p	ACBD5	91452	NM_001042473	92.3
hsa-miR-26a-5p	ZFC3H1	196441	NM_144982	92.2
hsa-miR-26a-5p	CPSF2	53981	NM_001322270	92.2
hsa-miR-26a-5p	MRAS	22808	NM_001085049	92.1
hsa-miR-26a-5p	RDH14	57665	NM_020905	92.1
hsa-miR-26a-5p	EIF4G2	1982	NM_001042559	92.1
hsa-miR-26a-5p	GPR52	9293	NM_005684	92.1
hsa-miR-26a-5p	TTC13	79573	NM_001122835	92.1
hsa-miR-26a-5p	CHAC1	79094	NM_001142776	92.0
hsa-miR-26a-5p	SPDYE5	442590	NM_001306141	92.0
hsa-miR-26a-5p	RFK	55312	NM_018339	92.0
hsa-miR-26a-5p	FGF18	8817	NM_003862	92.0
hsa-miR-26a-5p	FRAT2	23401	NM_012083	91.9
hsa-miR-26a-5p	KIAA2013	90231	NM_138346	91.9
hsa-miR-26a-5p	HGF	3082	NM_000601	91.8
hsa-miR-26a-5p	ATF2	1386	NM_001256090	91.8
hsa-miR-26a-5p	SFXN1	94081	NM_001322981	91.8
hsa-miR-26a-5p	PRKCQ	5588	NM_001242413	91.7

hsa-miR-26a-5p	LRRC2	79442	NM_024512	91.7
hsa-miR-26a-5p	RCBTB1	55213	NM_018191	91.7
hsa-miR-26a-5p	HEPHL1	341208	NM_001098672	91.7
hsa-miR-26a-5p	AMOT	154796	NM_001113490	91.7
hsa-miR-26a-5p	RTF1	23168	NM_015138	91.6
hsa-miR-26a-5p	FA2H	79152	NM_024306	91.6
hsa-miR-26a-5p	TMEM106B	54664	NM_001134232	91.6
hsa-miR-26a-5p	MAN2A1	4124	NM_002372	91.5
hsa-miR-26a-5p	PTPRD	5789	NM_001040712	91.5
hsa-miR-26a-5p	SEMA6D	80031	NM_001198999	91.5
hsa-miR-26a-5p	RPGR	6103	NM_000328	91.5
hsa-miR-26a-5p	COL1A2	1278	NM_000089	91.4
hsa-miR-26a-5p	STYX	6815	NM_001130701	91.3
hsa-miR-26a-5p	SLC33A1	9197	NM_001190992	91.3
hsa-miR-26a-5p	MICAL3	57553	NM_015241	91.3
hsa-miR-26a-5p	ATP1A2	477	NM_000702	91.3
hsa-miR-26a-5p	TBC1D4	9882	NM_001286658	91.2
hsa-miR-26a-5p	RB1	5925	NM_000321	91.2
hsa-miR-26a-5p	MXI1	4601	NM_001008541	91.2
hsa-miR-26a-5p	ZDHHC20	253832	NM_001286638	91.2
hsa-miR-26a-5p	PRR5L	79899	NM_001160169	91.2
hsa-miR-26a-5p	BOD1	91272	NM_138369	91.1
hsa-miR-26a-5p	SV2C	22987	NM_001297716	91.1
hsa-miR-26a-5p	TMEM248	55069	NM_017994	91.1
hsa-miR-26a-5p	HSPA8	3312	NM_006597	91.0
hsa-miR-26a-5p	MARK1	4139	NM_001286126	91.0
hsa-miR-26a-5p	ZNF462	58499	NM_021224	91.0
hsa-miR-26a-5p	MFSD14A	64645	NM_033055	90.9
hsa-miR-26a-5p	RHOU	58480	NM_021205	90.9
hsa-miR-26a-5p	FAM172A	83989	NM_001163417	90.8
hsa-miR-26a-5p	SLC24A4	123041	NM_153646	90.8
hsa-miR-26a-5p	AKAP7	9465	NM_004842	90.7
hsa-miR-26a-5p	TMEM86A	144110	NM_153347	90.7
hsa-miR-26a-5p	BFAR	51283	NM_016561	90.7
hsa-miR-26a-5p	ADAM23	8745	NM_003812	90.7
hsa-miR-26a-5p	SENP5	205564	NM_001308045	90.6
hsa-miR-26a-5p	CXADR	1525	NM_001207065	90.4
hsa-miR-26a-5p	PHLDB2	90102	NM_001134437	90.3
hsa-miR-26a-5p	FANCF	2188	NM_022725	90.3
hsa-miR-26a-5p	MCL1	4170	NM_001197320	90.3
hsa-miR-26a-5p	MTTP	4547	NM_000253	90.2
hsa-miR-26a-5p	FGD1	2245	NM_004463	90.2
hsa-miR-26a-5p	PARP14	54625	NM_017554	90.2
hsa-miR-26a-5p	MIER1	57708	NM_001278215	90.2
hsa-miR-26a-5p	BEND4	389206	NM_001159547	90.2
hsa-miR-26a-5p	SEPTIN10	151011	NM_001321499	90.1
hsa-miR-338-5p	PHC3	80012	NM_001308116	99.9
hsa-miR-338-5p	HACE1	57531	NM_001321080	99.5
hsa-miR-338-5p	PCDH20	64881	NM_022843	99.4
hsa-miR-338-5p	HEMGN	55363	NM_018437	99.3

hsa-miR-338-5p	CCN2	1490	NM_001901	99.3
hsa-miR-338-5p	SMG8	55181	NM_018149	99.3
hsa-miR-338-5p	SPRY2	10253	NM_001318536	99.3
hsa-miR-338-5p	STRN	6801	NM_003162	99.2
hsa-miR-338-5p	SLIT2	9353	NM_001289135	99.1
hsa-miR-338-5p	CUL2	8453	NM_001198777	98.9
hsa-miR-338-5p	PPP6R3	55291	NM_001164160	98.7
hsa-miR-338-5p	HIVEP1	3096	NM_002114	98.7
hsa-miR-338-5p	STAG2	10735	NM_001042749	98.7
hsa-miR-338-5p	TNPO1	3842	NM_002270	98.6
hsa-miR-338-5p	SOX6	55553	NM_001145811	98.6
hsa-miR-338-5p	MBD5	55777	NM_018328	98.3
hsa-miR-338-5p	MBLAC2	153364	NM_203406	98.0
hsa-miR-338-5p	RAB6B	51560	NM_016577	98.0
hsa-miR-338-5p	NUFIP2	57532	NM_020772	97.9
hsa-miR-338-5p	MSTN	2660	NM_005259	97.9
hsa-miR-338-5p	FAM214A	56204	NM_001286495	97.8
hsa-miR-338-5p	ZFX	7543	NM_001178084	97.8
hsa-miR-338-5p	GTPBP10	85865	NM_001042717	97.6
hsa-miR-338-5p	TSHZ1	10194	NM_001308210	97.5
hsa-miR-338-5p	LSM6	11157	NM_007080	97.5
hsa-miR-338-5p	RB1CC1	9821	NM_001083617	97.3
hsa-miR-338-5p	ESRRG	2104	NM_001134285	97.2
hsa-miR-338-5p	WDR33	55339	NM_001006623	97.2
hsa-miR-338-5p	ADM	133	NM_001124	97.1
hsa-miR-338-5p	WDR3	10885	NM_006784	97.1
hsa-miR-338-5p	SP3	6670	NM_001017371	97.1
hsa-miR-338-5p	RAB28	9364	NM_001017979	97.0
hsa-miR-338-5p	RNF138	51444	NM_001191324	96.9
hsa-miR-338-5p	LRATD1	151354	NM_145175	96.8
hsa-miR-338-5p	TLK1	9874	NM_001136554	96.8
hsa-miR-338-5p	ADAMTS17	170691	NM_139057	96.7
hsa-miR-338-5p	ARID4B	51742	NM_016374	96.7
hsa-miR-338-5p	COX15	1355	NM_001320974	96.6
hsa-miR-338-5p	AZIN1	51582	NM_001301668	96.5
hsa-miR-338-5p	RAB1A	5861	NM_004161	96.5
hsa-miR-338-5p	SEC63	11231	NM_007214	96.5
hsa-miR-338-5p	GTPBP2	54676	NM_001286216	96.5
hsa-miR-338-5p	LRRCC1	85444	NM_033402	96.5
hsa-miR-338-5p	ANKRD12	23253	NM_001083625	96.5
hsa-miR-338-5p	ANOS1	3730	NM_000216	96.3
hsa-miR-338-5p	ETS1	2113	NM_001143820	96.3
hsa-miR-338-5p	CNOT6L	246175	NM_001286790	96.2
hsa-miR-338-5p	NR5A2	2494	NM_001276464	96.1
hsa-miR-338-5p	PDE10A	10846	NM_001130690	96.1
hsa-miR-338-5p	SCN9A	6335	NM_002977	96.1
hsa-miR-338-5p	FMNL2	114793	NM_052905	96.0
hsa-miR-338-5p	LEPROT	54741	NM_001198681	96.0
hsa-miR-338-5p	ATP6V0A4	50617	NM_020632	95.8
hsa-miR-338-5p	RHEB	6009	NM_005614	95.8

hsa-miR-338-5p	KAT2B	8850	NM_003884	95.7
hsa-miR-338-5p	PALLD	23022	NM_001166108	95.6
hsa-miR-338-5p	ID1	3397	NM_181353	95.6
hsa-miR-338-5p	NEK7	140609	NM_133494	95.6
hsa-miR-338-5p	GTF2A1	2957	NM_001278940	95.6
hsa-miR-338-5p	WDR72	256764	NM_001277176	95.5
hsa-miR-338-5p	FBXO4	26272	NM_033484	95.5
hsa-miR-338-5p	MTRR	4552	NM_002454	95.5
hsa-miR-338-5p	FKBP14	55033	NM_017946	95.4
hsa-miR-338-5p	KDM4B	23030	NM_015015	95.4
hsa-miR-338-5p	DGKH	160851	NM_001204504	95.3
hsa-miR-338-5p	ESRP1	54845	NM_001034915	95.2
hsa-miR-338-5p	USP25	29761	NM_001283041	95.2
hsa-miR-338-5p	DDX59	83479	NM_001031725	95.1
hsa-miR-338-5p	ZNF543	125919	NM_213598	95.1
hsa-miR-338-5p	GABRB2	2561	NM_000813	95.0
hsa-miR-338-5p	COMMD3-BMI1	100532731	NM_001204062	95.0
hsa-miR-338-5p	NR1D1	9572	NM_021724	95.0
hsa-miR-338-5p	H3-5	440093	NM_001013699	94.9
hsa-miR-338-5p	CA2	760	NM_000067	94.8
hsa-miR-338-5p	TASOR	23272	NM_015224	94.8
hsa-miR-338-5p	SIRT1	23411	NM_001142498	94.8
hsa-miR-338-5p	CNTN4	152330	NM_001206955	94.5
hsa-miR-338-5p	TSHZ3	57616	NM_020856	94.5
hsa-miR-338-5p	H3-3B	3021	NM_005324	94.5
hsa-miR-338-5p	FEM1C	56929	NM_020177	94.3
hsa-miR-338-5p	PTPN9	5780	NM_002833	94.3
hsa-miR-338-5p	CTTN	2017	NM_005231	94.3
hsa-miR-338-5p	CLIP1	6249	NM_001247997	94.0
hsa-miR-338-5p	WIF1	11197	NM_007191	94.0
hsa-miR-338-5p	BCL9	607	NM_004326	94.0
hsa-miR-338-5p	LRP2	4036	NM_004525	93.9
hsa-miR-338-5p	LRRC58	116064	NM_001099678	93.8
hsa-miR-338-5p	UBE2K	3093	NM_001111112	93.8
hsa-miR-338-5p	GUCY1A1	2982	NM_000856	93.8
hsa-miR-338-5p	INO80D	54891	NM_017759	93.8
hsa-miR-338-5p	FEZ1	9638	NM_022549	93.7
hsa-miR-338-5p	ATRN	8455	NM_139321	93.7
hsa-miR-338-5p	DENND4A	10260	NM_001144823	93.6
hsa-miR-338-5p	ABCA8	10351	NM_001288985	93.6
hsa-miR-338-5p	HECTD2	143279	NM_173497	93.4
hsa-miR-338-5p	HSPA5	3309	NM_005347	93.4
hsa-miR-338-5p	DDX46	9879	NM_001300860	93.4
hsa-miR-338-5p	BCL2L11	10018	NM_001204107	93.4
hsa-miR-338-5p	ZNF770	54989	NM_014106	93.4
hsa-miR-338-5p	MBNL1	4154	NM_207297	93.3
hsa-miR-338-5p	CAST	831	NM_001042440	93.3
hsa-miR-338-5p	BAHCC1	57597	NM_001291324	93.3
hsa-miR-338-5p	CCDC186	55088	NM_001321829	93.3
hsa-miR-338-5p	MEF2C	4208	NM_001131005	93.2

hsa-miR-338-5p	MYCL	4610	NM_001033081	93.2
hsa-miR-338-5p	ENPP2	5168	NM_001040092	93.2
hsa-miR-338-5p	SENP7	57337	NM_001077203	93.1
hsa-miR-338-5p	CREB1	1385	NM_004379	93.1
hsa-miR-338-5p	CREB3L1	90993	NM_052854	93.0
hsa-miR-338-5p	SPRY4	81848	NM_001127496	93.0
hsa-miR-338-5p	ORC6	23594	NM_014321	93.0
hsa-miR-338-5p	MNX1	3110	NM_001165255	93.0
hsa-miR-338-5p	SCML2	10389	NM_006089	92.9
hsa-miR-338-5p	CHM	1121	NM_000390	92.9
hsa-miR-338-5p	TAOK1	57551	NM_020791	92.9
hsa-miR-338-5p	AP1B1	162	NM_001127	92.9
hsa-miR-338-5p	CENPF	1063	NM_016343	92.8
hsa-miR-338-5p	TUT4	23318	NM_001009881	92.8
hsa-miR-338-5p	ZNF711	7552	NM_021998	92.8
hsa-miR-338-5p	SGMS1	259230	NM_147156	92.7
hsa-miR-338-5p	PTGDR	5729	NM_000953	92.7
hsa-miR-338-5p	BTBD1	53339	NM_001011885	92.7
hsa-miR-338-5p	SH2D1A	4068	NM_001114937	92.6
hsa-miR-338-5p	KLF2	10365	NM_016270	92.6
hsa-miR-338-5p	CHD1	1105	NM_001270	92.6
hsa-miR-338-5p	RING1	6015	NM_002931	92.5
hsa-miR-338-5p	DBT	1629	NM_001918	92.4
hsa-miR-338-5p	LOC100287896	100287896	NM_001319240	92.4
hsa-miR-338-5p	TOB1	10140	NM_001243877	92.4
hsa-miR-338-5p	USP13	8975	NM_003940	92.4
hsa-miR-338-5p	RAB3C	115827	NM_001317915	92.3
hsa-miR-338-5p	ENAH	55740	NM_001008493	92.3
hsa-miR-338-5p	JAM2	58494	NM_001270407	92.1
hsa-miR-338-5p	MAP3K2	10746	NM_006609	92.0
hsa-miR-338-5p	NXPH1	30010	NM_152745	92.0
hsa-miR-338-5p	COL4A3	1285	NM_000091	92.0
hsa-miR-338-5p	PAN3	255967	NM_175854	92.0
hsa-miR-338-5p	WASF1	8936	NM_001024934	92.0
hsa-miR-338-5p	COBLL1	22837	NM_001278458	91.9
hsa-miR-338-5p	B3GALT6	126792	NM_080605	91.9
hsa-miR-338-5p	ATP2C1	27032	NM_001199179	91.8
hsa-miR-338-5p	NAB1	4664	NM_001321312	91.8
hsa-miR-338-5p	CD9	928	NM_001769	91.8
hsa-miR-338-5p	BBX	56987	NM_001142568	91.8
hsa-miR-338-5p	PURA	5813	NM_005859	91.7
hsa-miR-338-5p	MED13	9969	NM_005121	91.7
hsa-miR-338-5p	FAM135B	51059	NM_015912	91.7
hsa-miR-338-5p	MAPK9	5601	NM_002752	91.7
hsa-miR-338-5p	SLIT3	6586	NM_001271946	91.6
hsa-miR-338-5p	KLF10	7071	NM_001032282	91.6
hsa-miR-338-5p	WDR47	22911	NM_001142550	91.5
hsa-miR-338-5p	CDYL2	124359	NM_152342	91.5
hsa-miR-338-5p	LDB2	9079	NM_001130834	91.4
hsa-miR-338-5p	ANKS1B	56899	NM_001204065	91.4

hsa-miR-338-5p	PRR11	55771	NM_018304	91.2
hsa-miR-338-5p	BICD2	23299	NM_001003800	91.2
hsa-miR-338-5p	WDFY3	23001	NM_014991	91.2
hsa-miR-338-5p	LRRC7	57554	NM_020794	91.1
hsa-miR-338-5p	MAGI3	260425	NM_001142782	91.1
hsa-miR-338-5p	TMED5	50999	NM_001167830	91.1
hsa-miR-338-5p	NSUN7	79730	NM_024677	91.1
hsa-miR-338-5p	ITGB2	3689	NM_000211	91.0
hsa-miR-338-5p	CCDC6	8030	NM_005436	91.0
hsa-miR-338-5p	FGL2	10875	NM_006682	91.0
hsa-miR-338-5p	KALRN	8997	NM_001322988	90.9
hsa-miR-338-5p	CCDC126	90693	NM_138771	90.9
hsa-miR-338-5p	FNDC3B	64778	NM_001135095	90.9
hsa-miR-338-5p	NDFIP1	80762	NM_030571	90.9
hsa-miR-338-5p	ARAP2	116984	NM_015230	90.8
hsa-miR-338-5p	GTF3C3	9330	NM_012086	90.8
hsa-miR-338-5p	PTPRM	5797	NM_001105244	90.8
hsa-miR-338-5p	PTCH1	5727	NM_000264	90.8
hsa-miR-338-5p	ARHGAP23	57636	NM_001199417	90.8
hsa-miR-338-5p	MAP4K3	8491	NM_001270425	90.8
hsa-miR-338-5p	PI4K2B	55300	NM_018323	90.8
hsa-miR-338-5p	PIKFYVE	200576	NM_015040	90.8
hsa-miR-338-5p	APCS	325	NM_001639	90.8
hsa-miR-338-5p	HOXA10	3206	NM_018951	90.7
hsa-miR-338-5p	LTBR	4055	NM_001270987	90.7
hsa-miR-338-5p	SLC49A4	84925	NM_032839	90.7
hsa-miR-338-5p	GRIA2	2891	NM_000826	90.7
hsa-miR-338-5p	GPATCH2L	55668	NM_001322030	90.6
hsa-miR-338-5p	MTF1	4520	NM_005955	90.6
hsa-miR-338-5p	EOMES	8320	NM_001278182	90.6
hsa-miR-338-5p	EBF3	253738	NM_001005463	90.6
hsa-miR-338-5p	PICALM	8301	NM_001008660	90.5
hsa-miR-338-5p	KRTAP4-8	728224	NM_031960	90.5
hsa-miR-338-5p	RMI1	80010	NM_024945	90.5
hsa-miR-338-5p	KRTAP4-11	653240	NM_033059	90.5
hsa-miR-338-5p	AIMP1	9255	NM_001142415	90.5
hsa-miR-338-5p	ZC3H12C	85463	NM_033390	90.4
hsa-miR-338-5p	PREPL	9581	NM_001042385	90.4
hsa-miR-338-5p	AAK1	22848	NM_014911	90.3
hsa-miR-338-5p	SH3BGRL	6451	NM_003022	90.3
hsa-miR-338-5p	HOXA5	3202	NM_019102	90.2
hsa-miR-338-5p	ZFAND5	7763	NM_001102420	90.2
hsa-miR-338-5p	CSRNP3	80034	NM_001172173	90.2
hsa-miR-338-5p	PKN2	5586	NM_001320707	90.1
hsa-miR-671-3p	SHH	6469	NM_000193	92.1
hsa-miR-342-3p	GXYLT1	283464	NM_001099650	96.4
hsa-miR-342-3p	KCNA4	3739	NM_002233	96.3
hsa-miR-342-3p	UBE2D2	7322	NM_003339	96.1
hsa-miR-342-3p	ATXN7	6314	NM_000333	95.5
hsa-miR-342-3p	EEA1	8411	NM_003566	95.3

hsa-miR-342-3p	DTNBP1	84062	NM_001271667	95.2
hsa-miR-342-3p	EPC1	80314	NM_001272004	95.1
hsa-miR-342-3p	RRM2	6241	NM_001034	94.6
hsa-miR-342-3p	IBTK	25998	NM_001300906	94.4
hsa-miR-342-3p	TXK	7294	NM_003328	94.2
hsa-miR-342-3p	RICTOR	253260	NM_001285439	94.2
hsa-miR-342-3p	ANKRD49	54851	NM_017704	94.1
hsa-miR-342-3p	RFX3	5991	NM_134428	93.8
hsa-miR-342-3p	KCTD6	200845	NM_001128214	93.7
hsa-miR-342-3p	RGS4	5999	NM_001102445	93.6
hsa-miR-342-3p	CA12	771	NM_001218	93.5
hsa-miR-342-3p	MAP3K7CL	56911	NM_001286623	93.5
hsa-miR-342-3p	PLEKHB2	55041	NM_001100623	93.5
hsa-miR-342-3p	KDM6B	23135	NM_001080424	93.4
hsa-miR-342-3p	ZNF225	7768	NM_001321685	93.2
hsa-miR-342-3p	HTR2C	3358	NM_001256761	93.0
hsa-miR-342-3p	TGIF1	7050	NM_001278682	92.9
hsa-miR-342-3p	TTBK2	146057	NM_173500	92.2
hsa-miR-342-3p	ZNF740	283337	NM_001004304	92.1
hsa-miR-342-3p	DHRSX	207063	NM_145177	92.1
hsa-miR-342-3p	CASP2	835	NM_001224	92.0
hsa-miR-342-3p	S100A7A	338324	NM_176823	91.9
hsa-miR-342-3p	ZNF568	374900	NM_001204838	91.9
hsa-miR-342-3p	JADE1	79960	NM_001287437	91.7
hsa-miR-342-3p	CACNA1C	775	NM_000719	91.3
hsa-miR-342-3p	ID4	3400	NM_001546	90.9
hsa-miR-342-3p	MRFAP1	93621	NM_001272053	90.8
hsa-miR-342-3p	MEX3A	92312	NM_001093725	90.5
hsa-miR-342-3p	ZFP28	140612	NM_020828	90.3
hsa-miR-342-3p	PDE12	201626	NM_001322176	90.2
hsa-miR-342-3p	ATG10	83734	NM_001131028	90.1

Table S10. miRDB-derived predicted targets (score ≥ 90) of each miRNA used for the pathway-level enrichment analysis for PsA and C comparison.

miRNA	Gene	Entrez ID	RefSeq ID	miRDB Target Score
hsa-miR-10b-5p	UNC13C	57472	NM_001303241	98.3
hsa-miR-10b-5p	ZNF680	7022	NM_003222	97.6
hsa-miR-10b-5p	ZNF773	51379	NM_015986	96.4
hsa-miR-10b-5p	CUEDC1	55084	NM_018013	96.1
hsa-miR-10b-5p	CCDC85C	3742	NM_002235	96.1
hsa-miR-10b-5p	C2orf80	54898	NM_017770	95.2
hsa-miR-10b-5p	C11orf91	64641	NM_022659	94.4
hsa-miR-10b-5p	NA	167691	NM_001122769	93.0

hsa-miR-10b-5p	ZNF763	2649	NM_001489	92.6
hsa-miR-10b-5p	CCDC88C	55137	NM_001321825	91.6
hsa-miR-10b-5p	ZNF181	6885	NM_145332	91.5
hsa-miR-10b-5p	TMEM265	161357	NM_001113498	91.4
hsa-miR-10b-5p	SHISA7	60682	NM_001281439	91.4
hsa-miR-10b-5p	RASSF3	1398	NM_005206	90.6
hsa-miR-10b-5p	SMIM15	57722	NM_020962	90.4
hsa-miR-197-3p	CCDC50	7547	NM_003413	98.1
hsa-miR-197-3p	DENND6A	79183	NM_001039199	97.3
hsa-miR-197-3p	SEPTIN10	6886	NM_001287347	95.9
hsa-miR-197-3p	PPARGC1B	3556	NM_001167930	95.7
hsa-miR-197-3p	TMEM154	79801	NM_001324318	95.6
hsa-miR-197-3p	FAM76A	56256	NM_019605	95.4
hsa-miR-197-3p	OLIG3	54802	NM_001312691	95.0
hsa-miR-197-3p	FAM117B	6760	NM_001007559	94.8
hsa-miR-197-3p	NPNT	100862671	NM_001256829	94.0
hsa-miR-197-3p	FAT3	2107	NM_001256302	93.9
hsa-miR-197-3p	LCA5	54497	NM_019024	93.5
hsa-miR-197-3p	WFDC13	54455	NM_018994	93.5
hsa-miR-197-3p	CYYR1	523	NM_001690	93.4
hsa-miR-197-3p	ZFYVE27	1500	NM_001085458	92.7
hsa-miR-197-3p	FAM92A	4205	NM_001130926	92.6
hsa-miR-197-3p	JAKMIP1	9118	NM_032727	92.6
hsa-miR-197-3p	NAA30	3441	NM_021068	91.9
hsa-miR-197-3p	TC2N	3451	NM_021268	91.8
hsa-miR-197-3p	AMER1	4899	NM_001040110	91.8
hsa-miR-197-3p	ZNF440	3486	NM_000598	91.7
hsa-miR-197-3p	CERS6	339318	NM_001029997	91.7
hsa-miR-197-3p	MARCHF8	151011	NM_001321496	91.6
hsa-miR-197-3p	THSD7A	317762	NM_001144995	91.4
hsa-miR-197-3p	DGKH	23348	NM_001130048	91.3
hsa-miR-197-3p	UBR1	55787	NM_018360	91.2
hsa-miR-197-3p	TPCN2	80778	NM_001286769	91.1
hsa-miR-197-3p	EML5	27443	NM_001290046	90.9
hsa-miR-197-3p	OLFM3	1207	NM_001293	90.9
hsa-miR-197-3p	PAQR3	7706	NM_005082	90.8
hsa-miR-197-3p	MDGA2	23760	NM_001284278	90.8
hsa-miR-197-3p	ZNF563	5981	NM_001204747	90.5
hsa-miR-197-3p	ARHGAP42	5599	NM_001278548	90.3
hsa-miR-425-5p	SFT2D1	729956	NM_001145176	99.6
hsa-miR-425-5p	TNRC18	22887	NM_001198850	98.6
hsa-miR-425-5p	HMCN1	4893	NM_002524	95.0
hsa-miR-425-5p	INTS14	988	NM_001253	94.9
hsa-miR-425-5p	NAV1	56271	NM_001080425	94.6
hsa-miR-425-5p	RPS6KL1	4217	NM_005923	94.6
hsa-miR-425-5p	NAV3	54880	NM_001123383	94.5
hsa-miR-425-5p	ZFAND4	116159	NM_001320768	94.0
hsa-miR-425-5p	UCN2	80823	NM_001142524	93.8
hsa-miR-425-5p	ZNF439	89894	NM_001193453	91.9
hsa-miR-425-5p	TMEM116	57630	NM_020870	90.6

hsa-miR-425-5p	ADO	23347	NM_015295	90.5
hsa-miR-425-5p	FAM167A	1073	NM_001243645	90.4
hsa-miR-425-5p	PHF6	6016	NM_001256820	90.2
hsa-miR-425-5p	TMEM164	5212	NM_001177972	90.1
hsa-miR-199a-5p	TMEM255A	780	NM_001202521	99.5
hsa-miR-199a-5p	XYLT1	79085	NM_024103	99.5
hsa-miR-199a-5p	ELOVL2	745	NM_001127392	99.0
hsa-miR-199a-5p	EBF2	79829	NM_001300800	98.8
hsa-miR-199a-5p	ASRGL1	284390	NM_001012753	98.8
hsa-miR-199a-5p	GRHL3	55327	NM_018362	97.9
hsa-miR-199a-5p	TNN	57419	NM_020689	97.6
hsa-miR-199a-5p	PACS1	4296	NM_002419	97.1
hsa-miR-199a-5p	MACO1	2651	NM_001491	96.8
hsa-miR-199a-5p	BCCIP	8165	NM_001242902	96.5
hsa-miR-199a-5p	BEX4	7743	NM_001278240	96.4
hsa-miR-199a-5p	TGIF2	55536	NM_001127370	96.3
hsa-miR-199a-5p	CNOT6	9365	NM_004795	95.6
hsa-miR-199a-5p	TXNDC16	9770	NM_170774	95.4
hsa-miR-199a-5p	CDCA7L	4059	NM_001013257	95.3
hsa-miR-199a-5p	SLC25A23	90594	NM_152262	95.1
hsa-miR-199a-5p	SHCBP1	220972	NM_001282866	95.1
hsa-miR-199a-5p	ZNF34	374928	NM_001304336	95.0
hsa-miR-199a-5p	IKZF5	79088	NM_001318056	94.7
hsa-miR-199a-5p	LGR4	3675	NM_002204	94.5
hsa-miR-199a-5p	HCN3	27300	NM_001320777	94.0
hsa-miR-199a-5p	LIN7C	2787	NM_005274	94.0
hsa-miR-199a-5p	SOBP	1182	NM_001243374	93.8
hsa-miR-199a-5p	TTPAL	118813	NM_001002261	93.4
hsa-miR-199a-5p	ARMT1	126070	NM_152357	93.3
hsa-miR-199a-5p	DUS1L	57584	NM_020824	93.3
hsa-miR-199a-5p	FIGN	1889	NM_001113347	92.9
hsa-miR-199a-5p	CCNL1	9052	NM_003979	92.9
hsa-miR-199a-5p	KIAA1217	7105	NM_001278740	92.8
hsa-miR-199a-5p	GPR158	9658	NM_014643	92.7
hsa-miR-199a-5p	IGDCC4	54502	NM_001098634	92.7
hsa-miR-199a-5p	SERTAD4	7283	NM_001070	92.6
hsa-miR-199a-5p	BCOR	93	NM_001106	92.2
hsa-miR-199a-5p	SCYL2	4074	NM_001207024	92.2
hsa-miR-199a-5p	CRACD	9559	NM_001035260	92.1
hsa-miR-199a-5p	CFAP97	23063	NM_001318328	92.1
hsa-miR-199a-5p	ZNF426	93550	NM_001282905	91.9
hsa-miR-199a-5p	ARHGAP21	10630	NM_001006624	91.9
hsa-miR-199a-5p	LRCH2	27065	NM_001040101	91.7
hsa-miR-199a-5p	SH3RF1	26053	NM_001127231	91.5
hsa-miR-199a-5p	GOLPH3L	2241	NM_001308028	91.4
hsa-miR-199a-5p	TBL1XR1	147837	NM_145276	91.2
hsa-miR-199a-5p	ELMOD1	3840	NM_002268	91.2
hsa-miR-199a-5p	BHLHB9	440193	NM_001080414	91.1
hsa-miR-199a-5p	KLHL4	6655	NM_006939	91.0
hsa-miR-199a-5p	CYREN	83872	NM_031935	90.9

hsa-miR-199a-5p	FGD6	4783	NM_001289999	90.6
hsa-miR-199a-5p	CREB3L2	80150	NM_001083926	90.6
hsa-miR-199a-5p	SMAP1	57018	NM_020307	90.6
hsa-miR-199a-5p	AKIP1	8323	NM_001164615	90.5
hsa-miR-199a-5p	PHF12	27254	NM_014460	90.5
hsa-miR-199a-5p	FOXJ2	5454	NM_005604	90.4
hsa-miR-199a-5p	TXLNG	5362	NM_025179	90.4
hsa-miR-199a-5p	SAR1A	8945	NM_001256856	90.4
hsa-miR-199a-5p	SLC24A3	9352	NM_004786	90.4
hsa-miR-199a-5p	NAA40	283349	NM_178169	90.2
hsa-miR-203a-3p	AKAP1	2977	NM_000855	99.5
hsa-miR-203a-3p	RBM47	201799	NM_152680	99.4
hsa-miR-203a-3p	OPN3	51719	NM_001130849	99.4
hsa-miR-203a-3p	ZNF516	8573	NM_001126054	99.3
hsa-miR-203a-3p	SRA1	9037	NM_003966	99.2
hsa-miR-203a-3p	SDK2	221981	NM_015204	99.0
hsa-miR-203a-3p	VAPA	6102	NM_006915	98.3
hsa-miR-203a-3p	CELF3	10933	NM_001265603	98.1
hsa-miR-203a-3p	FGFR1OP	10236	NM_001102397	98.0
hsa-miR-203a-3p	SEMA5A	5144	NM_001165899	97.9
hsa-miR-203a-3p	RPGRIP1L	29880	NM_001142364	97.5
hsa-miR-203a-3p	VPS26A	7447	NM_003385	97.3
hsa-miR-203a-3p	ARID4B	160851	NM_001204505	97.1
hsa-miR-203a-3p	TSPAN6	361	NM_001317384	97.0
hsa-miR-203a-3p	FBXO42	197131	NM_174916	97.0
hsa-miR-203a-3p	INA	5634	NM_001039091	96.9
hsa-miR-203a-3p	MAP3K13	5874	NM_004163	96.7
hsa-miR-203a-3p	LEF1	118427	NM_001288821	96.6
hsa-miR-203a-3p	PITPNC1	57482	NM_020722	96.5
hsa-miR-203a-3p	TRIM25	1847	NM_004419	96.4
hsa-miR-203a-3p	TXNL1	6591	NM_003068	96.4
hsa-miR-203a-3p	ZIC3	1833	NM_004950	96.3
hsa-miR-203a-3p	SLC4A4	4214	NM_005921	96.2
hsa-miR-203a-3p	PITPNB	54583	NM_022051	96.1
hsa-miR-203a-3p	CLOCK	7707	NM_021964	96.0
hsa-miR-203a-3p	MEX3C	123036	NM_001128595	96.0
hsa-miR-203a-3p	MGAT4A	11051	NM_007006	95.9
hsa-miR-203a-3p	ZNF544	57822	NM_021180	95.6
hsa-miR-203a-3p	SATB2	26045	NM_015564	95.5
hsa-miR-203a-3p	TOB2	9643	NM_001142418	95.4
hsa-miR-203a-3p	TPPP	10011	NM_001035235	95.4
hsa-miR-203a-3p	EGLN1	340252	NM_001130022	95.3
hsa-miR-203a-3p	CLSTN3	8671	NM_001098484	95.2
hsa-miR-203a-3p	HNRNPR	9218	NM_194434	95.2
hsa-miR-203a-3p	ALG5	64376	NM_001271840	95.1
hsa-miR-203a-3p	KDM5D	3617	NM_001282368	95.0
hsa-miR-203a-3p	FOXJ3	11333	NM_014891	94.9
hsa-miR-203a-3p	PHLDA1	11116	NM_194429	94.8
hsa-miR-203a-3p	DOCK9	50804	NM_001301210	94.7
hsa-miR-203a-3p	NSG1	57544	NM_001160047	94.7

hsa-miR-203a-3p	MORF4L1	9645	NM_001282666	94.6
hsa-miR-203a-3p	SEC62	167	NM_001131	94.6
hsa-miR-203a-3p	ADAMTS5	10168	NM_001024855	94.5
hsa-miR-203a-3p	NUDT21	9746	NM_014718	94.3
hsa-miR-203a-3p	PDPN	9407	NM_004262	94.3
hsa-miR-203a-3p	VIRMA	55681	NM_001317784	94.0
hsa-miR-203a-3p	PKP4	3886	NM_002280	93.7
hsa-miR-203a-3p	MORF4L2	7994	NM_006766	93.7
hsa-miR-203a-3p	MINDY2	389073	NM_001099334	93.7
hsa-miR-203a-3p	ATMIN	25962	NM_015496	93.7
hsa-miR-203a-3p	PRKAG2	143872	NM_152432	93.5
hsa-miR-203a-3p	TMEM184B	55219	NM_001282564	93.4
hsa-miR-203a-3p	NUMBL	6342	NM_001007099	93.4
hsa-miR-203a-3p	MYEF2	84295	NM_001015877	93.2
hsa-miR-203a-3p	PDE7B	57631	NM_001243963	93.2
hsa-miR-203a-3p	AUTS2	56647	NM_078469	93.2
hsa-miR-203a-3p	TUBG1	1308	NM_000494	93.2
hsa-miR-203a-3p	WAPL	23435	NM_007375	93.2
hsa-miR-203a-3p	PAPSS2	5592	NM_001098512	93.1
hsa-miR-203a-3p	CSDC2	57649	NM_001033561	93.1
hsa-miR-203a-3p	BTRC	4281	NM_000381	93.1
hsa-miR-203a-3p	ZDHHC17	51422	NM_001040633	93.0
hsa-miR-203a-3p	PURG	79624	NM_001286562	93.0
hsa-miR-203a-3p	KAT6A	2180	NM_001286708	92.9
hsa-miR-203a-3p	DAAM1	22822	NM_007350	92.6
hsa-miR-203a-3p	ZNF189	1960	NM_001199880	92.5
hsa-miR-203a-3p	NOCT	54629	NM_001040450	92.4
hsa-miR-203a-3p	FGF23	2889	NM_198679	92.4
hsa-miR-203a-3p	UBE2D3	1447	NM_001302770	92.3
hsa-miR-203a-3p	MICAL2	8038	NM_001288973	92.2
hsa-miR-203a-3p	UBP1	1656	NM_001257191	92.1
hsa-miR-203a-3p	NRN1	122830	NM_001011713	92.1
hsa-miR-203a-3p	HEATR5B	201627	NM_152678	92.1
hsa-miR-203a-3p	TRIT1	100131378	NM_001166692	91.9
hsa-miR-203a-3p	VSNL1	1749	NM_005221	91.8
hsa-miR-203a-3p	ZNF148	1871	NM_001243076	91.8
hsa-miR-203a-3p	GALNT7	164237	NM_172005	91.7
hsa-miR-203a-3p	ZMYM4	6059	NM_001040876	91.7
hsa-miR-203a-3p	FUT9	9465	NM_016377	91.7
hsa-miR-203a-3p	AKAP7	6925	NM_001083962	91.7
hsa-miR-203a-3p	SLC4A7	7323	NM_181892	91.7
hsa-miR-203a-3p	RASSF2	8825	NM_001324423	91.7
hsa-miR-203a-3p	GSKIP	152137	NM_174908	91.6
hsa-miR-203a-3p	GPRC5A	5337	NM_001130081	91.6
hsa-miR-203a-3p	CRLF3	137392	NM_001283034	91.6
hsa-miR-203a-3p	TARDBP	51527	NM_001271904	91.5
hsa-miR-203a-3p	TMEM69	120114	NM_001008781	91.5
hsa-miR-203a-3p	LIN7A	4246	NM_002407	91.5
hsa-miR-203a-3p	KL	6660	NM_001261414	91.4
hsa-miR-203a-3p	AHCYL2	51249	NM_016486	91.4

hsa-miR-203a-3p	FOXP1	57587	NM_020827	91.4
hsa-miR-203a-3p	STRN3	81556	NM_001136043	91.4
hsa-miR-203a-3p	LRRTM2	56062	NM_019117	91.3
hsa-miR-203a-3p	SHOC2	2554	NM_000806	91.2
hsa-miR-203a-3p	FZD6	3796	NM_001098511	91.2
hsa-miR-203a-3p	SMCHD1	29942	NM_001323311	91.1
hsa-miR-203a-3p	TASOR	25819	NM_012118	91.0
hsa-miR-203a-3p	CECR2	63923	NM_022093	91.0
hsa-miR-203a-3p	PDAP1	11096	NM_007038	91.0
hsa-miR-203a-3p	CASK	3891	NM_001300810	90.9
hsa-miR-203a-3p	CAB39	152559	NM_001040202	90.9
hsa-miR-203a-3p	TMPRSS11D	6678	NM_001309443	90.7
hsa-miR-203a-3p	DLL1	64118	NM_022156	90.5
hsa-miR-203a-3p	ZNF197	9175	NM_001242317	90.5
hsa-miR-203a-3p	PPFIA1	3848	NM_006121	90.5
hsa-miR-203a-3p	ADAM12	2632	NM_000158	90.3
hsa-miR-203a-3p	TSN	1008	NM_001317222	90.3
hsa-miR-203a-3p	CAMTA1	23596	NM_014322	90.2
hsa-miR-203a-3p	DNM1L	9060	NM_001015880	90.2
hsa-miR-203a-3p	SLC25A27	7095	NM_003262	90.1
hsa-miR-34a-5p	PRPS2	57657	NM_020897	100.0
hsa-miR-34a-5p	VAMP2	199870	NM_001143912	99.8
hsa-miR-34a-5p	EGR3	5910	NM_001100426	99.7
hsa-miR-34a-5p	NFIL3	54549	NM_001144952	99.6
hsa-miR-34a-5p	RFC1	83648	NM_053279	99.6
hsa-miR-34a-5p	SCGB2A1	28514	NM_005618	99.6
hsa-miR-34a-5p	GBE1	6992	NM_021959	99.4
hsa-miR-34a-5p	MYRF	1875	NM_001083588	99.3
hsa-miR-34a-5p	CRK	4194	NM_001204171	99.3
hsa-miR-34a-5p	GABRA1	6857	NM_001135805	99.0
hsa-miR-34a-5p	FER	6327	NM_004588	99.0
hsa-miR-34a-5p	RRAS	89795	NM_001024383	98.8
hsa-miR-34a-5p	KIF2A	11320	NM_012214	98.6
hsa-miR-34a-5p	LMAN1	23314	NM_001172509	98.5
hsa-miR-34a-5p	ECE1	5818	NM_002855	98.4
hsa-miR-34a-5p	DLX5	4613	NM_001293228	98.3
hsa-miR-34a-5p	CSN2	4233	NM_000245	98.3
hsa-miR-34a-5p	PDE4D	55366	NM_018490	98.2
hsa-miR-34a-5p	ANK3	777	NM_000721	98.1
hsa-miR-34a-5p	DDR1	2319	NM_004475	98.1
hsa-miR-34a-5p	SCN2B	89796	NM_001167738	98.0
hsa-miR-34a-5p	PTGIS	60436	NM_001199513	97.7
hsa-miR-34a-5p	KCNA6	11189	NM_001172648	97.7
hsa-miR-34a-5p	PLD1	55690	NM_018026	97.7
hsa-miR-34a-5p	PURB	64131	NM_022166	97.6
hsa-miR-34a-5p	CACNA1E	2281	NM_001322963	97.6
hsa-miR-34a-5p	ACSL1	6237	NM_006270	97.4
hsa-miR-34a-5p	HNF4A	8502	NM_001005476	97.2
hsa-miR-34a-5p	FUT8	6844	NM_014232	97.2
hsa-miR-34a-5p	DDX6	4355	NM_001278370	97.2

hsa-miR-34a-5p	M6PR	23382	NM_001130720	96.7
hsa-miR-34a-5p	TFAP2C	NA	NM_033517	96.7
hsa-miR-34a-5p	ABR	29	NM_001322842	96.7
hsa-miR-34a-5p	IL6R	10690	NM_006581	96.5
hsa-miR-34a-5p	TAL1	404093	NM_001271875	96.3
hsa-miR-34a-5p	MAP3K1	26207	NM_181671	96.2
hsa-miR-34a-5p	CTNND2	4300	NM_001286691	96.2
hsa-miR-34a-5p	KRT1	23261	NM_015215	95.5
hsa-miR-34a-5p	CACNB3	2530	NM_004480	95.3
hsa-miR-34a-5p	IMPG1	10766	NM_016272	95.2
hsa-miR-34a-5p	MLLT3	51320	NM_016626	94.9
hsa-miR-34a-5p	MAP3K7	255743	NM_001033047	94.8
hsa-miR-34a-5p	RAP1GDS1	79718	NM_001321193	94.6
hsa-miR-34a-5p	FLOT2	6734	NM_001177842	94.6
hsa-miR-34a-5p	MAP3K5	27086	NM_001244813	94.5
hsa-miR-34a-5p	GCNT2	7342	NM_001128160	94.5
hsa-miR-34a-5p	RAB27B	78996	NM_001243749	94.5
hsa-miR-34a-5p	FOXN2	9253	NM_001289979	94.3
hsa-miR-34a-5p	MID1	51176	NM_001130713	94.2
hsa-miR-34a-5p	SPARC	152789	NM_001306134	94.2
hsa-miR-34a-5p	CDC5L	2556	NM_000808	94.1
hsa-miR-34a-5p	NRAS	55026	NM_001104544	94.1
hsa-miR-34a-5p	POU3F2	56243	NM_019590	94.0
hsa-miR-34a-5p	ABCE1	84629	NM_001080495	94.0
hsa-miR-34a-5p	SERPINF2	55785	NM_018351	94.0
hsa-miR-34a-5p	VIT	55531	NM_018712	93.9
hsa-miR-34a-5p	ITGA3	11076	NM_007030	93.8
hsa-miR-34a-5p	CLCN3	3344	NM_002158	93.8
hsa-miR-34a-5p	SNAI1	133522	NM_001172698	93.7
hsa-miR-34a-5p	IFNA4	9481	NM_001204051	93.7
hsa-miR-34a-5p	FKBP1B	6615	NM_005985	93.7
hsa-miR-34a-5p	ASIC2	40	NM_001094	93.5
hsa-miR-34a-5p	CDK6	3172	NM_000457	93.5
hsa-miR-34a-5p	CDH10	2845	NM_005295	93.5
hsa-miR-34a-5p	MYCN	51809	NM_017423	93.4
hsa-miR-34a-5p	CRISP1	651	NM_001201	93.2
hsa-miR-34a-5p	KPNA4	23002	NM_001270520	93.1
hsa-miR-34a-5p	HSPA1B	9202	NM_005095	93.0
hsa-miR-34a-5p	KRT35	23272	NM_001112736	93.0
hsa-miR-34a-5p	RFX3	83694	NM_031464	93.0
hsa-miR-34a-5p	IL1RAP	10059	NM_001278463	92.8
hsa-miR-34a-5p	PLXNA2	55810	NM_018416	92.7
hsa-miR-34a-5p	SRPRA	161436	NM_183387	92.7
hsa-miR-34a-5p	GUCY1A2	8500	NM_003626	92.7
hsa-miR-34a-5p	TAF4B	253782	NM_001256126	92.5
hsa-miR-34a-5p	SS18	167826	NM_175747	92.5
hsa-miR-34a-5p	GABRA3	6875	NM_001293725	92.5
hsa-miR-34a-5p	RP2	84890	NM_032804	92.5
hsa-miR-34a-5p	EPYC	5345	NM_001165921	92.4
hsa-miR-34a-5p	KITLG	29966	NM_001083893	92.4

hsa-miR-34a-5p	IFNA17	9497	NM_001258379	92.3
hsa-miR-34a-5p	SCP2	90226	NM_033199	92.3
hsa-miR-34a-5p	NECTIN1	64764	NM_194071	92.3
hsa-miR-34a-5p	PPP2R3A	56672	NM_001206646	92.3
hsa-miR-34a-5p	SOX5	150864	NM_173511	92.2
hsa-miR-34a-5p	TCF4	440279	NM_001080534	92.2
hsa-miR-34a-5p	MPP2	51742	NM_001206794	92.1
hsa-miR-34a-5p	MEF2A	25829	NM_001195071	91.9
hsa-miR-34a-5p	AQP4	784	NM_000725	91.9
hsa-miR-34a-5p	NRF1	55204	NM_018178	91.7
hsa-miR-34a-5p	COL17A1	3998	NM_005570	91.6
hsa-miR-34a-5p	RAPGEF1	8284	NM_001146705	91.6
hsa-miR-34a-5p	MAP3K11	51299	NM_001278710	91.6
hsa-miR-34a-5p	CLNS1A	3570	NM_000565	91.6
hsa-miR-34a-5p	ATP6V1A	1021	NM_001145306	91.6
hsa-miR-34a-5p	KRT85	23300	NM_001300728	91.6
hsa-miR-34a-5p	MDM4	23390	NM_015336	91.5
hsa-miR-34a-5p	GPR22	8074	NM_020638	91.4
hsa-miR-34a-5p	ACVR2B	288	NM_001149	91.4
hsa-miR-34a-5p	DUSP5	5523	NM_001190447	91.2
hsa-miR-34a-5p	SOS2	139285	NM_152424	91.2
hsa-miR-34a-5p	PPP1R11	643155	NM_001048249	91.2
hsa-miR-34a-5p	E2F5	5814	NM_033224	91.1
hsa-miR-34a-5p	BMP3	1501	NM_001288715	91.0
hsa-miR-34a-5p	CFL2	3304	NM_005346	90.7
hsa-miR-34a-5p	BCAM	23322	NM_001127897	90.7
hsa-miR-34a-5p	SYT1	219931	NM_139075	90.7
hsa-miR-34a-5p	MET	27115	NM_018945	90.7
hsa-miR-34a-5p	PRKG1	56681	NM_001142648	90.5
hsa-miR-34a-5p	NR6A1	7247	NM_001261401	90.5
hsa-miR-34a-5p	MAPK8	57512	NM_020752	90.5
hsa-miR-34a-5p	GNG5	8036	NM_007373	90.4
hsa-miR-34a-5p	CTNND1	4254	NM_000899	90.3
hsa-miR-34a-5p	SNAI2	113402	NM_145169	90.2
hsa-miR-34a-5p	E2F3	5740	NM_000961	90.2
hsa-miR-34a-5p	RIT1	84187	NM_032227	90.1
hsa-miR-34a-5p	ETF1	5991	NM_001282116	90.1
hsa-miR-34a-5p	IGFBP3	9575	NM_001267843	90.0

Table S11. miRDB-derived predicted targets (score ≥ 90) of each miRNA used for the pathway-level enrichment analysis for PsV and C comparison.

miRNA	Gene	Entrez ID	RefSeq ID	miRDB Target Score
hsa-miR-423-5p	FOXP4	116113	NM_001012426	98.7
hsa-miR-423-5p	FAM222B	55731	NM_001077498	98.6
hsa-miR-423-5p	PLCB1	23236	NM_182734	97.0

hsa-miR-423-5p	ADGRL1	22859	NM_001008701	96.5
hsa-miR-423-5p	EVC	2121	NM_001306090	96.0
hsa-miR-423-5p	PLA2G6	8398	NM_001004426	94.9
hsa-miR-423-5p	SCN4A	6329	NM_000334	94.4
hsa-miR-423-5p	FRMD3	257019	NM_001244960	94.2
hsa-miR-423-5p	TSPAN11	441631	NM_001080509	94.2
hsa-miR-423-5p	C1QTNF6	114904	NM_031910	93.8
hsa-miR-423-5p	TMEM150A	129303	NM_001031738	93.8
hsa-miR-423-5p	TRABD2B	388630	NM_001194986	93.5
hsa-miR-423-5p	CALM3	808	NM_005184	92.9
hsa-miR-423-5p	ASB6	140459	NM_177999	92.7
hsa-miR-423-5p	SLC20A2	6575	NM_001257180	92.6
hsa-miR-423-5p	HIC2	23119	NM_015094	92.5
hsa-miR-423-5p	ST6GALNAC6	30815	NM_001286999	92.3
hsa-miR-423-5p	NAT8L	339983	NM_178557	92.1
hsa-miR-423-5p	RIMS4	140730	NM_001205317	91.8
hsa-miR-423-5p	PLEKHO1	51177	NM_001304722	91.6
hsa-miR-423-5p	ODF3L1	161753	NM_175881	91.5
hsa-miR-423-5p	PGRMC2	10424	NM_006320	91.4
hsa-miR-423-5p	UBE2O	63893	NM_022066	91.2
hsa-miR-423-5p	NRSN2	80023	NM_001323679	91.2
hsa-miR-423-5p	NNAT	4826	NM_005386	90.9
hsa-miR-423-5p	STK40	83931	NM_001282546	90.7
hsa-miR-423-5p	MUL1	79594	NM_024544	90.6
hsa-miR-423-5p	SHANK1	50944	NM_016148	90.6
hsa-miR-423-5p	CELSR2	1952	NM_001408	90.3
hsa-miR-423-5p	DNALI1	7802	NM_003462	90.1
hsa-miR-423-5p	RAD9B	144715	NM_001286531	90.0
hsa-miR-335-5p	CASP7	840	NM_001227	97.1
hsa-miR-335-5p	SEPHS1	22929	NM_001195602	96.5
hsa-miR-335-5p	NRXN1	9378	NM_001135659	96.3
hsa-miR-335-5p	ZRANB1	54764	NM_017580	96.1
hsa-miR-335-5p	SRSF4	6429	NM_005626	95.6
hsa-miR-335-5p	FLT1	2321	NM_001159920	94.8
hsa-miR-335-5p	STAG1	10274	NM_005862	94.1
hsa-miR-335-5p	POU2F3	25833	NM_001244682	94.0
hsa-miR-335-5p	KRT81	3887	NM_002281	93.8
hsa-miR-335-5p	TTK	7272	NM_001166691	93.3
hsa-miR-335-5p	CHFR	55743	NM_001161344	93.0
hsa-miR-335-5p	GATA2	2624	NM_001145661	92.9
hsa-miR-335-5p	HOXD8	3234	NM_001199746	92.8
hsa-miR-335-5p	NXPH2	11249	NM_007226	92.5
hsa-miR-335-5p	VAPA	9218	NM_003574	92.3
hsa-miR-335-5p	USP24	23358	NM_015306	91.6
hsa-miR-335-5p	ARGLU1	55082	NM_018011	91.6
hsa-miR-335-5p	TNFSF11	8600	NM_003701	91.4
hsa-miR-335-5p	HAND1	9421	NM_004821	91.4
hsa-miR-335-5p	PTGES3L	100885848	NM_001142653	91.1
hsa-miR-335-5p	PSD3	23362	NM_015310	90.9
hsa-miR-335-5p	TMEM59	9528	NM_001305043	90.7

hsa-miR-335-5p	UBE2G1	7326	NM_003342	90.5
hsa-miR-335-5p	ATE1	11101	NM_001001976	90.2
hsa-miR-335-5p	TPST1	8460	NM_003596	90.2
hsa-miR-335-5p	RPRM	56475	NM_019845	90.2
hsa-miR-342-3p	GXYLT1	283464	NM_001099650	96.4
hsa-miR-342-3p	KCNA4	3739	NM_002233	96.3
hsa-miR-342-3p	UBE2D2	7322	NM_003339	96.1
hsa-miR-342-3p	ATXN7	6314	NM_000333	95.5
hsa-miR-342-3p	EEA1	8411	NM_003566	95.3
hsa-miR-342-3p	DTNBP1	84062	NM_001271667	95.2
hsa-miR-342-3p	EPC1	80314	NM_001272004	95.1
hsa-miR-342-3p	RRM2	6241	NM_001034	94.6
hsa-miR-342-3p	IBTK	25998	NM_001300906	94.4
hsa-miR-342-3p	TXK	7294	NM_003328	94.2
hsa-miR-342-3p	RICTOR	253260	NM_001285439	94.2
hsa-miR-342-3p	ANKRD49	54851	NM_017704	94.1
hsa-miR-342-3p	RFX3	5991	NM_134428	93.8
hsa-miR-342-3p	KCTD6	200845	NM_001128214	93.7
hsa-miR-342-3p	RGS4	5999	NM_001102445	93.6
hsa-miR-342-3p	CA12	771	NM_001218	93.5
hsa-miR-342-3p	MAP3K7CL	56911	NM_001286623	93.5
hsa-miR-342-3p	PLEKHB2	55041	NM_001100623	93.5
hsa-miR-342-3p	KDM6B	23135	NM_001080424	93.4
hsa-miR-342-3p	ZNF225	7768	NM_001321685	93.2
hsa-miR-342-3p	HTR2C	3358	NM_001256761	93.0
hsa-miR-342-3p	TGIF1	7050	NM_001278682	92.9
hsa-miR-342-3p	TTBK2	146057	NM_173500	92.2
hsa-miR-342-3p	ZNF740	283337	NM_001004304	92.1
hsa-miR-342-3p	DHRSX	207063	NM_145177	92.1
hsa-miR-342-3p	CASP2	835	NM_001224	92.0
hsa-miR-342-3p	S100A7A	338324	NM_176823	91.9
hsa-miR-342-3p	ZNF568	374900	NM_001204838	91.9
hsa-miR-342-3p	JADE1	79960	NM_001287437	91.7
hsa-miR-342-3p	CACNA1C	775	NM_000719	91.3
hsa-miR-342-3p	ID4	3400	NM_001546	90.9
hsa-miR-342-3p	MRFAP1	93621	NM_001272053	90.8
hsa-miR-342-3p	MEX3A	92312	NM_001093725	90.5
hsa-miR-342-3p	ZFP28	140612	NM_020828	90.3
hsa-miR-342-3p	PDE12	201626	NM_001322176	90.2
hsa-miR-342-3p	ATG10	83734	NM_001131028	90.1
hsa-miR-99b-5p	TRIB2	28951	NM_021643	95.8
hsa-miR-99b-5p	SMARCA5	8467	NM_003601	94.0
hsa-miR-99b-5p	KBTBD8	84541	NM_032505	93.7
hsa-miR-99b-5p	MTOR	2475	NM_004958	92.6
hsa-miR-99b-5p	HS3ST2	9956	NM_006043	91.1
hsa-miR-99b-5p	RAVER2	55225	NM_018211	90.7
hsa-miR-99b-5p	BAZ2A	11176	NM_001300905	90.4
hsa-miR-17-5p	DCBLD2	131566	NM_080927	92.3
hsa-miR-17-5p	CMKLR1	1240	NM_001142343	90.3
hsa-miR-17-5p	MAP3K9	4293	NM_001284230	90.2

hsa-miR-17-5p	SLMAP	7871	NM_001311179	90.1
hsa-miR-17-5p	MAPK1	5594	NM_002745	90.1
hsa-miR-18a-5p	GLRB	2743	NM_000824	99.3
hsa-miR-18a-5p	NEDD9	4739	NM_001142393	97.8
hsa-miR-18a-5p	CDK19	23097	NM_001300960	97.8
hsa-miR-18a-5p	PATJ	10207	NM_176877	97.2
hsa-miR-18a-5p	DICER1	23405	NM_001195573	97.1
hsa-miR-18a-5p	CREBL2	1389	NM_001310	96.2
hsa-miR-18a-5p	MAP7D1	55700	NM_001286365	96.1
hsa-miR-18a-5p	FAM3C	10447	NM_001040020	96.0
hsa-miR-18a-5p	IRF2	3660	NM_002199	95.8
hsa-miR-18a-5p	HIF1A	3091	NM_181054	95.7
hsa-miR-18a-5p	KCNA1	3736	NM_000217	95.4
hsa-miR-18a-5p	ERI1	90459	NM_153332	95.2
hsa-miR-18a-5p	FRYL	285527	NM_015030	95.2
hsa-miR-18a-5p	HMGCS1	3157	NM_001098272	95.1
hsa-miR-18a-5p	TNFAIP3	7128	NM_001270507	95.0
hsa-miR-18a-5p	PHF19	26147	NM_001286842	94.9
hsa-miR-18a-5p	ESR1	2099	NM_000125	94.3
hsa-miR-18a-5p	TRAPPC8	22878	NM_014939	93.9
hsa-miR-18a-5p	EPB41L1	2036	NM_001258329	93.6
hsa-miR-18a-5p	PNISR	25957	NM_001322405	93.4
hsa-miR-18a-5p	CCR2	729230	NM_001123396	93.2
hsa-miR-18a-5p	TRIOBP	11078	NM_001039141	93.1
hsa-miR-18a-5p	GCLC	2729	NM_001197115	93.0
hsa-miR-18a-5p	ADD3	120	NM_001121	92.7
hsa-miR-18a-5p	RAB5A	5868	NM_001292048	92.7
hsa-miR-18a-5p	HMBOX1	79618	NM_001324395	92.7
hsa-miR-18a-5p	DIP2C	22982	NM_014974	92.2
hsa-miR-18a-5p	RNF145	153830	NM_001199380	92.2
hsa-miR-18a-5p	GIGYF1	64599	NM_022574	91.8
hsa-miR-18a-5p	FBXL3	26224	NM_012158	91.1
hsa-miR-18a-5p	MAPK4	5596	NM_001292040	90.5
hsa-miR-18a-5p	ESCO2	157570	NM_001017420	90.4
hsa-miR-18a-5p	PNLIPRP3	119548	NM_001011709	90.3
hsa-miR-18a-5p	DPPA3	359787	NM_199286	90.0
hsa-miR-27a-3p	TRPV3	162514	NM_001258205	99.7
hsa-miR-27a-3p	PLK2	10769	NM_001252226	99.7
hsa-miR-27a-3p	AFF4	27125	NM_014423	99.7
hsa-miR-27a-3p	GCC2	9648	NM_181453	99.6
hsa-miR-27a-3p	GAB1	2549	NM_002039	99.6
hsa-miR-27a-3p	ARFGEF1	10565	NM_006421	99.6
hsa-miR-27a-3p	DCUN1D4	23142	NM_001040402	99.6
hsa-miR-27a-3p	AKIRIN1	79647	NM_001136275	99.4
hsa-miR-27a-3p	NRK	203447	NM_198465	99.3
hsa-miR-27a-3p	PDS5B	23047	NM_015032	99.2
hsa-miR-27a-3p	RGPD6	729540	NM_001123363	99.1
hsa-miR-27a-3p	MBTD1	54799	NM_017643	99.0
hsa-miR-27a-3p	RGPD5	84220	NM_005054	99.0
hsa-miR-27a-3p	CDS1	1040	NM_001263	99.0

hsa-miR-27a-3p	TAB3	257397	NM_152787	98.9
hsa-miR-27a-3p	BEX3	27018	NM_001282674	98.8
hsa-miR-27a-3p	RPS6KA5	9252	NM_001322227	98.7
hsa-miR-27a-3p	PHLPP2	23035	NM_001289003	98.6
hsa-miR-27a-3p	SOS1	6654	NM_005633	98.5
hsa-miR-27a-3p	SEMA6A	57556	NM_001300780	98.5
hsa-miR-27a-3p	USP42	84132	NM_032172	98.4
hsa-miR-27a-3p	PLEKHJ1	55111	NM_018049	98.4
hsa-miR-27a-3p	ZBTB34	403341	NM_001099270	98.4
hsa-miR-27a-3p	EYA4	2070	NM_001301012	98.3
hsa-miR-27a-3p	EYA1	2138	NM_000503	98.2
hsa-miR-27a-3p	KCNK2	3776	NM_001017424	98.2
hsa-miR-27a-3p	GPAM	57678	NM_001244949	98.2
hsa-miR-27a-3p	ST6GALNAC3	256435	NM_152996	98.1
hsa-miR-27a-3p	RGPD4	285190	NM_182588	98.0
hsa-miR-27a-3p	FBXW7	55294	NM_001013415	97.9
hsa-miR-27a-3p	ABHD17C	58489	NM_021214	97.8
hsa-miR-27a-3p	RGPD8	727851	NM_001164463	97.6
hsa-miR-27a-3p	KIAA1109	84162	NM_015312	97.5
hsa-miR-27a-3p	RNF139	11236	NM_007218	97.5
hsa-miR-27a-3p	KDM7A	80853	NM_030647	97.4
hsa-miR-27a-3p	SBF2	81846	NM_030962	97.4
hsa-miR-27a-3p	C2CD2	25966	NM_015500	97.4
hsa-miR-27a-3p	GRIN2D	2906	NM_000836	97.4
hsa-miR-27a-3p	FOXA3	3171	NM_004497	97.3
hsa-miR-27a-3p	ONECUT2	9480	NM_004852	97.3
hsa-miR-27a-3p	SLC7A11	23657	NM_014331	97.2
hsa-miR-27a-3p	EHF	26298	NM_001206615	97.1
hsa-miR-27a-3p	DNAJC27	51277	NM_001198559	97.0
hsa-miR-27a-3p	TMBIM6	7009	NM_001098576	96.9
hsa-miR-27a-3p	PRR3	80742	NM_001077497	96.8
hsa-miR-27a-3p	BTG2	7832	NM_006763	96.7
hsa-miR-27a-3p	GATC	283459	NM_176818	96.7
hsa-miR-27a-3p	USP46	64854	NM_001134223	96.7
hsa-miR-27a-3p	ADAMTSL3	57188	NM_207517	96.6
hsa-miR-27a-3p	WNK3	65267	NM_001002838	96.6
hsa-miR-27a-3p	ACVR1C	130399	NM_001111031	96.5
hsa-miR-27a-3p	FBXO10	26267	NM_012166	96.5
hsa-miR-27a-3p	GOLM1	51280	NM_016548	96.5
hsa-miR-27a-3p	CKAP4	10970	NM_006825	96.4
hsa-miR-27a-3p	PLEKHH1	57475	NM_020715	96.4
hsa-miR-27a-3p	UGCG	7357	NM_003358	96.4
hsa-miR-27a-3p	ABHD6	57406	NM_001320126	96.4
hsa-miR-27a-3p	SEMA7A	8482	NM_001146029	96.4
hsa-miR-27a-3p	TFAP2B	7021	NM_003221	96.4
hsa-miR-27a-3p	EDRF1	26098	NM_001202438	96.3
hsa-miR-27a-3p	ZFHX3	463	NM_001164766	96.3
hsa-miR-27a-3p	SMAD9	4093	NM_001127217	96.2
hsa-miR-27a-3p	KLF3	51274	NM_016531	96.2
hsa-miR-27a-3p	UNKL	64718	NM_001193388	96.2

hsa-miR-27a-3p	GNS	2799	NM_002076	96.1
hsa-miR-27a-3p	EPS8	2059	NM_004447	96.1
hsa-miR-27a-3p	ADORA2B	136	NM_000676	96.1
hsa-miR-27a-3p	PLCL2	23228	NM_001144382	96.1
hsa-miR-27a-3p	SZRD1	26099	NM_001114600	96.0
hsa-miR-27a-3p	PEG10	23089	NM_001040152	96.0
hsa-miR-27a-3p	HIVEP3	59269	NM_001127714	95.8
hsa-miR-27a-3p	VAV2	7410	NM_001134398	95.8
hsa-miR-27a-3p	IPMK	253430	NM_152230	95.7
hsa-miR-27a-3p	CDR2	1039	NM_001802	95.7
hsa-miR-27a-3p	ZHX1	11244	NM_001017926	95.6
hsa-miR-27a-3p	SLITRK1	114798	NM_001281503	95.6
hsa-miR-27a-3p	PDIA5	10954	NM_006810	95.5
hsa-miR-27a-3p	RREB1	6239	NM_001003698	95.5
hsa-miR-27a-3p	COG7	91949	NM_153603	95.5
hsa-miR-27a-3p	DNAJC13	23317	NM_015268	95.5
hsa-miR-27a-3p	SNRNP27	11017	NM_006857	95.4
hsa-miR-27a-3p	CSRP2	1466	NM_001300965	95.4
hsa-miR-27a-3p	RALGAPA2	57186	NM_020343	95.4
hsa-miR-27a-3p	ZNF800	168850	NM_176814	95.3
hsa-miR-27a-3p	RO60	6738	NM_004600	95.3
hsa-miR-27a-3p	SSH1	54434	NM_018984	95.3
hsa-miR-27a-3p	B4GALT3	8703	NM_001199873	95.2
hsa-miR-27a-3p	GRM5	2915	NM_000842	95.0
hsa-miR-27a-3p	ASPH	444	NM_001164750	95.0
hsa-miR-27a-3p	CCNG1	900	NM_004060	95.0
hsa-miR-27a-3p	C20orf194	25943	NM_001009984	95.0
hsa-miR-27a-3p	GSPT1	2935	NM_001130006	94.9
hsa-miR-27a-3p	TSC22D2	9819	NM_001303264	94.8
hsa-miR-27a-3p	ITSN2	50618	NM_006277	94.8
hsa-miR-27a-3p	CREBRF	153222	NM_153607	94.8
hsa-miR-27a-3p	SLC25A25	114789	NM_001006641	94.7
hsa-miR-27a-3p	MARK1	4139	NM_001286124	94.6
hsa-miR-27a-3p	STYK1	55359	NM_018423	94.6
hsa-miR-27a-3p	BRPF3	27154	NM_015695	94.6
hsa-miR-27a-3p	ST3GAL6	10402	NM_001271142	94.6
hsa-miR-27a-3p	NR2F6	2063	NM_005234	94.6
hsa-miR-27a-3p	MAP1B	4131	NM_001324255	94.6
hsa-miR-27a-3p	VSIG10	54621	NM_019086	94.5
hsa-miR-27a-3p	RMND5A	64795	NM_022780	94.4
hsa-miR-27a-3p	CTH	1491	NM_001190463	94.4
hsa-miR-27a-3p	INSM2	84684	NM_032594	94.4
hsa-miR-27a-3p	SHE	126669	NM_001010846	94.4
hsa-miR-27a-3p	ZCCHC24	219654	NM_153367	94.3
hsa-miR-27a-3p	DOT1L	84444	NM_032482	94.3
hsa-miR-27a-3p	ZSCAN26	7741	NM_001023560	94.3
hsa-miR-27a-3p	PTGER3	5733	NM_198715	94.3
hsa-miR-27a-3p	UBE2V1	7335	NM_001032288	94.2
hsa-miR-27a-3p	B3GNT7	93010	NM_145236	94.2
hsa-miR-27a-3p	NPAS3	64067	NM_001164749	94.1

hsa-miR-27a-3p	NIPAL4	348938	NM_001099287	94.0
hsa-miR-27a-3p	KIAA1211L	343990	NM_207362	94.0
hsa-miR-27a-3p	TMCC1	23023	NM_001017395	94.0
hsa-miR-27a-3p	APPBP2	10513	NM_001282476	94.0
hsa-miR-27a-3p	AGFG1	3267	NM_001135187	93.8
hsa-miR-27a-3p	GRIA3	2892	NM_000828	93.8
hsa-miR-27a-3p	PCDH9	5101	NM_001318374	93.8
hsa-miR-27a-3p	BICC1	80114	NM_001080512	93.7
hsa-miR-27a-3p	ENDOU	8909	NM_001172439	93.7
hsa-miR-27a-3p	SLC39A11	201266	NM_001159770	93.6
hsa-miR-27a-3p	ARF3	377	NM_001659	93.5
hsa-miR-27a-3p	PPARG	5468	NM_005037	93.5
hsa-miR-27a-3p	ARHGEF26	26084	NM_001251962	93.5
hsa-miR-27a-3p	STAB2	55576	NM_017564	93.4
hsa-miR-27a-3p	TEAD1	7003	NM_021961	93.4
hsa-miR-27a-3p	REPS1	85021	NM_001128617	93.4
hsa-miR-27a-3p	ELFN2	114794	NM_052906	93.4
hsa-miR-27a-3p	SLC6A1	6529	NM_003042	93.4
hsa-miR-27a-3p	ATAD2B	54454	NM_001242338	93.4
hsa-miR-27a-3p	PARD6B	84612	NM_032521	93.4
hsa-miR-27a-3p	C1orf52	148423	NM_198077	93.3
hsa-miR-27a-3p	EPB41L4A	64097	NM_022140	93.3
hsa-miR-27a-3p	LIFR	3977	NM_001127671	93.3
hsa-miR-27a-3p	SFRP1	6422	NM_003012	93.3
hsa-miR-27a-3p	RCAN2	10231	NM_001251973	93.3
hsa-miR-27a-3p	FAM184A	79632	NM_001100411	93.3
hsa-miR-27a-3p	TPR	7175	NM_003292	93.2
hsa-miR-27a-3p	FBXO34	55030	NM_017943	93.2
hsa-miR-27a-3p	DIPK1A	388650	NM_001006605	93.1
hsa-miR-27a-3p	GRIA4	2893	NM_000829	93.1
hsa-miR-27a-3p	ARHGAP32	9743	NM_001142685	93.0
hsa-miR-27a-3p	NABP1	64859	NM_001254736	93.0
hsa-miR-27a-3p	MTURN	222166	NM_152793	93.0
hsa-miR-27a-3p	MATN3	4148	NM_002381	92.9
hsa-miR-27a-3p	ACTA2	59	NM_001141945	92.9
hsa-miR-27a-3p	HMGCR	3156	NM_000859	92.7
hsa-miR-27a-3p	ADAMTS10	81794	NM_001282352	92.7
hsa-miR-27a-3p	PAQR9	344838	NM_198504	92.6
hsa-miR-27a-3p	HBEGF	1839	NM_001945	92.6
hsa-miR-27a-3p	GAREM1	64762	NM_001242409	92.6
hsa-miR-27a-3p	TRIM23	373	NM_033227	92.6
hsa-miR-27a-3p	RPS6KB1	6198	NM_001272042	92.5
hsa-miR-27a-3p	PPP4R2	151987	NM_001318025	92.5
hsa-miR-27a-3p	LPIN1	23175	NM_001261427	92.5
hsa-miR-27a-3p	LPAR6	10161	NM_001162497	92.5
hsa-miR-27a-3p	HAPLN1	1404	NM_001884	92.4
hsa-miR-27a-3p	ING5	84289	NM_032329	92.3
hsa-miR-27a-3p	TLK2	11011	NM_001284333	92.2
hsa-miR-27a-3p	MYT1	4661	NM_004535	92.2
hsa-miR-27a-3p	CA10	56934	NM_001082533	92.2

hsa-miR-27a-3p	SRGAP2	23380	NM_001170637	92.2
hsa-miR-27a-3p	NEMP2	100131211	NM_001142645	92.1
hsa-miR-27a-3p	LPCAT1	79888	NM_024830	92.1
hsa-miR-27a-3p	PLPP3	8613	NM_003713	92.1
hsa-miR-27a-3p	CEP135	9662	NM_025009	92.1
hsa-miR-27a-3p	MAP2K4	6416	NM_001281435	92.0
hsa-miR-27a-3p	NPEPPS	9520	NM_006310	91.9
hsa-miR-27a-3p	CACNG2	10369	NM_006078	91.9
hsa-miR-27a-3p	SNAP25	6616	NM_001322902	91.9
hsa-miR-27a-3p	PANK1	53354	NM_138316	91.9
hsa-miR-27a-3p	SOX11	6664	NM_003108	91.9
hsa-miR-27a-3p	DTNA	1837	NM_032980	91.9
hsa-miR-27a-3p	AK4	205	NM_001005353	91.8
hsa-miR-27a-3p	RNF141	50862	NM_016422	91.6
hsa-miR-27a-3p	CLK2	1196	NM_001294338	91.6
hsa-miR-27a-3p	CLCN3	1182	NM_001243372	91.6
hsa-miR-27a-3p	SCAF11	9169	NM_004719	91.6
hsa-miR-27a-3p	PCNX1	22990	NM_001308160	91.6
hsa-miR-27a-3p	MARC1	64757	NM_022746	91.6
hsa-miR-27a-3p	YWHAQ	10971	NM_006826	91.5
hsa-miR-27a-3p	LONRF1	91694	NM_152271	91.5
hsa-miR-27a-3p	NXT2	55916	NM_001242617	91.4
hsa-miR-27a-3p	MTMR4	9110	NM_004687	91.4
hsa-miR-27a-3p	NDUFS4	4724	NM_001318051	91.4
hsa-miR-27a-3p	OAF	220323	NM_178507	91.3
hsa-miR-27a-3p	SNX10	29887	NM_001199835	91.3
hsa-miR-27a-3p	PLCH1	23007	NM_001130961	91.3
hsa-miR-27a-3p	PKIA	5569	NM_006823	91.3
hsa-miR-27a-3p	SUCO	51430	NM_001282750	91.2
hsa-miR-27a-3p	COLEC10	10584	NM_001324095	91.2
hsa-miR-27a-3p	ARL4C	10123	NM_005737	91.2
hsa-miR-27a-3p	FRS3	10817	NM_006653	91.2
hsa-miR-27a-3p	HIC1	3090	NM_001098202	91.2
hsa-miR-27a-3p	NPTN	27020	NM_001161363	91.2
hsa-miR-27a-3p	SMOC2	64094	NM_001166412	91.1
hsa-miR-27a-3p	CIPC	85457	NM_033426	91.1
hsa-miR-27a-3p	PIK3CA	5290	NM_006218	91.1
hsa-miR-27a-3p	SORL1	6653	NM_003105	91.1
hsa-miR-27a-3p	TMEM189-UBE2V1	387522	NM_199203	91.0
hsa-miR-27a-3p	SOGA1	140710	NM_080627	91.0
hsa-miR-27a-3p	PSEN1	5663	NM_000021	91.0
hsa-miR-27a-3p	GPD2	2820	NM_000408	90.9
hsa-miR-27a-3p	MED12L	116931	NM_053002	90.9
hsa-miR-27a-3p	KLHL29	114818	NM_052920	90.9
hsa-miR-27a-3p	NGFR	4804	NM_002507	90.9
hsa-miR-27a-3p	MOCS3	27304	NM_014484	90.9
hsa-miR-27a-3p	GALNT3	2591	NM_004482	90.9
hsa-miR-27a-3p	LCOR	84458	NM_001170765	90.9
hsa-miR-27a-3p	DYNC2LI1	51626	NM_001193464	90.9
hsa-miR-27a-3p	EGFR	1956	NM_005228	90.9

hsa-miR-27a-3p	RELN	5649	NM_005045	90.8
hsa-miR-27a-3p	TXLNG	55787	NM_001168683	90.7
hsa-miR-27a-3p	IKZF2	22807	NM_001079526	90.7
hsa-miR-27a-3p	CREB1	1385	NM_134442	90.7
hsa-miR-27a-3p	SLC22A23	63027	NM_001286455	90.6
hsa-miR-27a-3p	PAX9	5083	NM_006194	90.6
hsa-miR-27a-3p	FAM133B	257415	NM_001040057	90.6
hsa-miR-27a-3p	ANK1	286	NM_000037	90.5
hsa-miR-27a-3p	COLGALT2	23127	NM_001303421	90.5
hsa-miR-27a-3p	TCIM	56892	NM_020130	90.4
hsa-miR-27a-3p	PLPPR1	54886	NM_017753	90.3
hsa-miR-27a-3p	SLC9B1	150159	NM_001100874	90.3
hsa-miR-27a-3p	FZD4	8322	NM_012193	90.3
hsa-miR-27a-3p	AQP11	282679	NM_173039	90.2
hsa-miR-27a-3p	KITLG	4254	NM_003994	90.2
hsa-miR-27a-3p	HSD17B12	51144	NM_016142	90.2
hsa-miR-27a-3p	KPNB1	3837	NM_001276453	90.2
hsa-miR-27a-3p	CAPN15	6650	NM_005632	90.1
hsa-miR-27a-3p	ZNF329	79673	NM_024620	90.1
hsa-miR-27a-3p	RARA	5914	NM_000964	90.1
hsa-miR-27a-3p	RSBN1L	222194	NM_198467	90.1
hsa-miR-27a-3p	CDH11	1009	NM_001308392	90.1
hsa-miR-27a-3p	RSPO3	84870	NM_032784	90.1
hsa-miR-27a-3p	TMEM9B	56674	NM_001286094	90.1
hsa-miR-451a	OSR1	130497	NM_145260	91.7
hsa-miR-199a-5p	DDR1	780	NM_001202521	99.5
hsa-miR-199a-5p	SLC25A23	79085	NM_024103	99.5
hsa-miR-199a-5p	MYRF	745	NM_001127392	99.0
hsa-miR-199a-5p	NAA40	79829	NM_001300800	98.8
hsa-miR-199a-5p	ZNF763	284390	NM_001012753	98.8
hsa-miR-199a-5p	LIN7C	55327	NM_018362	97.9
hsa-miR-199a-5p	SLC24A3	57419	NM_020689	97.6
hsa-miR-199a-5p	MAP3K11	4296	NM_002419	97.1
hsa-miR-199a-5p	GCNT2	2651	NM_001491	96.8
hsa-miR-199a-5p	AKAP1	8165	NM_001242902	96.5
hsa-miR-199a-5p	ZNF189	7743	NM_001278240	96.4
hsa-miR-199a-5p	CDCA7L	55536	NM_001127370	96.3
hsa-miR-199a-5p	KL	9365	NM_004795	95.6
hsa-miR-199a-5p	RASSF2	9770	NM_170774	95.4
hsa-miR-199a-5p	BCAM	4059	NM_001013257	95.3
hsa-miR-199a-5p	ZNF439	90594	NM_152262	95.1
hsa-miR-199a-5p	MARCHF8	220972	NM_001282866	95.1
hsa-miR-199a-5p	ZNF773	374928	NM_001304336	95.0
hsa-miR-199a-5p	ZNF426	79088	NM_001318056	94.7
hsa-miR-199a-5p	ITGA3	3675	NM_002204	94.5
hsa-miR-199a-5p	ZNF544	27300	NM_001320777	94.0
hsa-miR-199a-5p	GNG5	2787	NM_005274	94.0
hsa-miR-199a-5p	ZFYVE27	118813	NM_001002261	93.4
hsa-miR-199a-5p	ZNF440	126070	NM_152357	93.3
hsa-miR-199a-5p	ARHGAP21	57584	NM_020824	93.3

hsa-miR-199a-5p	ECE1	1889	NM_001113347	92.9
hsa-miR-199a-5p	GPRC5A	9052	NM_003979	92.9
hsa-miR-199a-5p	TSPAN6	7105	NM_001278740	92.8
hsa-miR-199a-5p	ZNF516	9658	NM_014643	92.7
hsa-miR-199a-5p	RBM47	54502	NM_001098634	92.7
hsa-miR-199a-5p	TUBG1	7283	NM_001070	92.6
hsa-miR-199a-5p	ACVR2B	93	NM_001106	92.2
hsa-miR-199a-5p	M6PR	4074	NM_001207024	92.2
hsa-miR-199a-5p	VPS26A	9559	NM_001035260	92.1
hsa-miR-199a-5p	WAPL	23063	NM_001318328	92.1
hsa-miR-199a-5p	PDPN	10630	NM_001006624	91.9
hsa-miR-199a-5p	NSG1	27065	NM_001040101	91.7
hsa-miR-199a-5p	AUTS2	26053	NM_001127231	91.5
hsa-miR-199a-5p	FER	2241	NM_001308028	91.4
hsa-miR-199a-5p	ZNF563	147837	NM_145276	91.2
hsa-miR-199a-5p	KPNA4	3840	NM_002268	91.2
hsa-miR-199a-5p	CCDC88C	440193	NM_001080414	91.1
hsa-miR-199a-5p	SOS2	6655	NM_006939	91.0
hsa-miR-199a-5p	HMCN1	83872	NM_031935	90.9
hsa-miR-199a-5p	NFIL3	4783	NM_001289999	90.6
hsa-miR-199a-5p	ASRGL1	80150	NM_001083926	90.6
hsa-miR-199a-5p	CCNL1	57018	NM_020307	90.6
hsa-miR-199a-5p	FZD6	8323	NM_001164615	90.5
hsa-miR-199a-5p	CSDC2	27254	NM_014460	90.5
hsa-miR-199a-5p	POU3F2	5454	NM_005604	90.4
hsa-miR-199a-5p	PLXNA2	5362	NM_025179	90.4
hsa-miR-199a-5p	BTRC	8945	NM_001256856	90.4
hsa-miR-199a-5p	TXNL1	9352	NM_004786	90.4
hsa-miR-199a-5p	RASSF3	283349	NM_178169	90.2
hsa-miR-30b-5p	STRIP1	85369	NM_001270768	90.4
hsa-miR-20a-5p	ZNFX1	57169	NM_021035	99.9
hsa-miR-20a-5p	FYCO1	79443	NM_024513	99.7
hsa-miR-20a-5p	GPR137C	283554	NM_001099652	99.7
hsa-miR-20a-5p	ENPP5	59084	NM_001290072	99.6
hsa-miR-20a-5p	PKD2	5311	NM_000297	99.6
hsa-miR-20a-5p	ANKRD52	283373	NM_173595	99.5
hsa-miR-20a-5p	NAPEPLD	222236	NM_001122838	99.5
hsa-miR-20a-5p	ZFYVE26	23503	NM_015346	99.5
hsa-miR-20a-5p	PDCD1LG2	80380	NM_025239	99.5
hsa-miR-20a-5p	SLC40A1	30061	NM_014585	99.5
hsa-miR-20a-5p	KCNB1	3745	NM_004975	99.4
hsa-miR-20a-5p	PTPN4	5775	NM_002830	99.3
hsa-miR-20a-5p	STK17B	9262	NM_004226	99.2
hsa-miR-20a-5p	RUFY2	55680	NM_017987	99.2
hsa-miR-20a-5p	TXNIP	10628	NM_001313972	99.2
hsa-miR-20a-5p	ARHGAP12	94134	NM_001270695	99.1
hsa-miR-20a-5p	TBC1D9	23158	NM_015130	99.1
hsa-miR-20a-5p	ZNF827	152485	NM_178835	99.1
hsa-miR-20a-5p	BRMS1L	84312	NM_032352	99.1
hsa-miR-20a-5p	RAB22A	57403	NM_020673	99.1

hsa-miR-20a-5p	VLDLR	7436	NM_001018056	99.0
hsa-miR-20a-5p	TBC1D20	128637	NM_144628	99.0
hsa-miR-20a-5p	NPAT	4863	NM_001321307	99.0
hsa-miR-20a-5p	KCNK10	54207	NM_021161	99.0
hsa-miR-20a-5p	NPAS2	4862	NM_002518	98.9
hsa-miR-20a-5p	SAR1B	51128	NM_001033503	98.9
hsa-miR-20a-5p	GPR6	2830	NM_001286099	98.8
hsa-miR-20a-5p	ITPRIPL2	162073	NM_001034841	98.8
hsa-miR-20a-5p	EZH1	2145	NM_001321079	98.8
hsa-miR-20a-5p	RRAGD	58528	NM_021244	98.8
hsa-miR-20a-5p	DENND10	404636	NM_001303111	98.7
hsa-miR-20a-5p	PLEKHA3	65977	NM_019091	98.7
hsa-miR-20a-5p	EPHA5	2044	NM_001281765	98.5
hsa-miR-20a-5p	ANKIB1	54467	NM_019004	98.4
hsa-miR-20a-5p	MKRN1	23608	NM_013446	98.4
hsa-miR-20a-5p	RGL1	23179	NM_001297669	98.4
hsa-miR-20a-5p	FCHO2	115548	NM_001146032	98.3
hsa-miR-20a-5p	RNF128	79589	NM_024539	98.3
hsa-miR-20a-5p	KIAA0513	9764	NM_001286565	98.2
hsa-miR-20a-5p	ZNF512B	57473	NM_020713	98.2
hsa-miR-20a-5p	ZNF367	195828	NM_153695	98.1
hsa-miR-20a-5p	NCOA3	8202	NM_001174087	98.0
hsa-miR-20a-5p	E2F1	1869	NM_005225	97.9
hsa-miR-20a-5p	EPHA4	2043	NM_001304536	97.9
hsa-miR-20a-5p	AKTIP	64400	NM_001012398	97.8
hsa-miR-20a-5p	FBXL5	26234	NM_001193534	97.8
hsa-miR-20a-5p	EMSY	56946	NM_001300942	97.7
hsa-miR-20a-5p	VANGL1	81839	NM_001172411	97.7
hsa-miR-20a-5p	ZBTB4	57659	NM_001128833	97.6
hsa-miR-20a-5p	SAMD12	401474	NM_001101676	97.6
hsa-miR-20a-5p	ATXN1L	342371	NM_001137675	97.4
hsa-miR-20a-5p	CCND1	595	NM_053056	97.4
hsa-miR-20a-5p	BNIP2	663	NM_001320674	97.3
hsa-miR-20a-5p	VASH2	79805	NM_001136474	97.3
hsa-miR-20a-5p	ZFYVE9	9372	NM_004799	97.3
hsa-miR-20a-5p	ATG16L1	55054	NM_001190266	97.3
hsa-miR-20a-5p	ELK4	2005	NM_001973	97.2
hsa-miR-20a-5p	EIF5A2	56648	NM_020390	97.2
hsa-miR-20a-5p	ITGA4	3676	NM_000885	97.1
hsa-miR-20a-5p	SLC24A2	25769	NM_001193288	97.1
hsa-miR-20a-5p	LRIG1	26018	NM_015541	97.0
hsa-miR-20a-5p	MFSD8	256471	NM_152778	96.9
hsa-miR-20a-5p	FGD4	121512	NM_001304480	96.9
hsa-miR-20a-5p	RASGRF2	5924	NM_006909	96.9
hsa-miR-20a-5p	NIBAN1	116496	NM_052966	96.8
hsa-miR-20a-5p	EFCAB14	9813	NM_014774	96.8
hsa-miR-20a-5p	ZXDA	7789	NM_007156	96.8
hsa-miR-20a-5p	GLIS3	169792	NM_001042413	96.7
hsa-miR-20a-5p	FRS2	10818	NM_001042555	96.7
hsa-miR-20a-5p	MYT1L	23040	NM_001303052	96.7

hsa-miR-20a-5p	GPR63	81491	NM_001143957	96.7
hsa-miR-20a-5p	TRIP10	9322	NM_001288962	96.6
hsa-miR-20a-5p	KMT2B	9757	NM_014727	96.5
hsa-miR-20a-5p	NR2C2	7182	NM_001291694	96.5
hsa-miR-20a-5p	CMTR2	55783	NM_001099642	96.5
hsa-miR-20a-5p	TNFRSF21	27242	NM_014452	96.5
hsa-miR-20a-5p	FJX1	24147	NM_014344	96.5
hsa-miR-20a-5p	RBL2	5934	NM_005611	96.5
hsa-miR-20a-5p	ANKRD29	147463	NM_001308238	96.4
hsa-miR-20a-5p	AGTPBP1	23287	NM_001286715	96.4
hsa-miR-20a-5p	ZNF704	619279	NM_001033723	96.4
hsa-miR-20a-5p	CHRM2	1129	NM_000739	96.4
hsa-miR-20a-5p	TRIM36	55521	NM_001300752	96.4
hsa-miR-20a-5p	VSX1	30813	NM_014588	96.3
hsa-miR-20a-5p	TGFBR2	7048	NM_001024847	96.2
hsa-miR-20a-5p	ATAD2	29028	NM_014109	96.2
hsa-miR-20a-5p	DPYSL5	56896	NM_001253723	96.1
hsa-miR-20a-5p	MAP3K14	9020	NM_003954	96.1
hsa-miR-20a-5p	UBXN2A	165324	NM_181713	96.0
hsa-miR-20a-5p	PRRG1	5638	NM_000950	96.0
hsa-miR-20a-5p	USP31	57478	NM_020718	96.0
hsa-miR-20a-5p	ZFP91	80829	NM_001197051	96.0
hsa-miR-20a-5p	SPRED1	161742	NM_152594	95.9
hsa-miR-20a-5p	LAPTM4A	9741	NM_014713	95.9
hsa-miR-20a-5p	ZNF652	22834	NM_001145365	95.9
hsa-miR-20a-5p	U2SURP	23350	NM_001080415	95.8
hsa-miR-20a-5p	SERTAD2	9792	NM_014755	95.7
hsa-miR-20a-5p	FGD5	152273	NM_001320276	95.7
hsa-miR-20a-5p	CNOT4	4850	NM_001008225	95.7
hsa-miR-20a-5p	ZBTB7A	51341	NM_001317990	95.6
hsa-miR-20a-5p	EGLN3	112399	NM_001308103	95.5
hsa-miR-20a-5p	HS3ST5	222537	NM_153612	95.5
hsa-miR-20a-5p	EPHA7	2045	NM_001288629	95.5
hsa-miR-20a-5p	CEP97	79598	NM_001303401	95.4
hsa-miR-20a-5p	LAMA3	3909	NM_000227	95.4
hsa-miR-20a-5p	SALL3	27164	NM_171999	95.4
hsa-miR-20a-5p	ANKRD33B	651746	NM_001164440	95.3
hsa-miR-20a-5p	NAGK	55577	NM_017567	95.3
hsa-miR-20a-5p	PPP6C	5537	NM_001123355	95.3
hsa-miR-20a-5p	PTHLH	5744	NM_198965	95.2
hsa-miR-20a-5p	PGM2L1	283209	NM_173582	95.2
hsa-miR-20a-5p	TRIP11	9321	NM_001321851	95.2
hsa-miR-20a-5p	NCKAP5	344148	NM_207363	95.2
hsa-miR-20a-5p	F3	2152	NM_001178096	95.1
hsa-miR-20a-5p	RAB11FIP5	26056	NM_015470	95.1
hsa-miR-20a-5p	SLC17A7	57030	NM_020309	95.1
hsa-miR-20a-5p	RASL11B	65997	NM_023940	95.0
hsa-miR-20a-5p	IRF9	10379	NM_006084	95.0
hsa-miR-20a-5p	FSD1L	83856	NM_001145313	94.9
hsa-miR-20a-5p	UEVLD	55293	NM_001040697	94.9

hsa-miR-20a-5p	SPOPL	339745	NM_001001664	94.9
hsa-miR-20a-5p	PFKP	5214	NM_001242339	94.9
hsa-miR-20a-5p	SALL1	6299	NM_001127892	94.7
hsa-miR-20a-5p	DUSP2	1844	NM_004418	94.7
hsa-miR-20a-5p	HAS2	3037	NM_005328	94.7
hsa-miR-20a-5p	LIMK1	3984	NM_001204426	94.7
hsa-miR-20a-5p	MKNK2	2872	NM_017572	94.6
hsa-miR-20a-5p	MASTL	84930	NM_001172303	94.6
hsa-miR-20a-5p	ARMC8	25852	NM_001267041	94.5
hsa-miR-20a-5p	NKIRAS1	28512	NM_020345	94.5
hsa-miR-20a-5p	LIMA1	51474	NM_001113546	94.5
hsa-miR-20a-5p	FAT2	2196	NM_001447	94.4
hsa-miR-20a-5p	SLC46A3	283537	NM_181785	94.4
hsa-miR-20a-5p	SNTB2	6645	NM_006750	94.4
hsa-miR-20a-5p	CLIP4	79745	NM_001287527	94.4
hsa-miR-20a-5p	TOPORS	10210	NM_001195622	94.4
hsa-miR-20a-5p	DDHD1	80821	NM_001160147	94.3
hsa-miR-20a-5p	AGO1	26523	NM_001317123	94.3
hsa-miR-20a-5p	ANKH	56172	NM_054027	94.3
hsa-miR-20a-5p	ADARB1	104	NM_001112	94.3
hsa-miR-20a-5p	KLHL2	11275	NM_001161521	94.2
hsa-miR-20a-5p	TMEM127	55654	NM_001193304	94.2
hsa-miR-20a-5p	PDE3B	5140	NM_000922	94.2
hsa-miR-20a-5p	PAK5	57144	NM_020341	94.2
hsa-miR-20a-5p	PCDH15	65217	NM_001142771	94.1
hsa-miR-20a-5p	PPP1R3B	79660	NM_001201329	94.1
hsa-miR-20a-5p	GPR137B	7107	NM_003272	94.1
hsa-miR-20a-5p	KMT2A	4297	NM_001197104	94.0
hsa-miR-20a-5p	ZBTB20	26137	NM_001164342	94.0
hsa-miR-20a-5p	BTG3	10950	NM_001130914	94.0
hsa-miR-20a-5p	FBXO48	554251	NM_001024680	93.9
hsa-miR-20a-5p	JPT1	51155	NM_001002033	93.7
hsa-miR-20a-5p	UBE2Q2	92912	NM_001145335	93.7
hsa-miR-20a-5p	LDLRAP1	26119	NM_015627	93.7
hsa-miR-20a-5p	XRN1	54464	NM_001282857	93.7
hsa-miR-20a-5p	RAB5B	5869	NM_001252036	93.7
hsa-miR-20a-5p	RORC	6097	NM_001001523	93.7
hsa-miR-20a-5p	BTBD10	84280	NM_001297741	93.7
hsa-miR-20a-5p	HAUS8	93323	NM_001011699	93.7
hsa-miR-20a-5p	FAM219B	57184	NM_001321922	93.7
hsa-miR-20a-5p	TM2D2	83877	NM_001024380	93.6
hsa-miR-20a-5p	HBP1	26959	NM_001244262	93.6
hsa-miR-20a-5p	HEG1	57493	NM_020733	93.6
hsa-miR-20a-5p	NIN	51199	NM_020921	93.6
hsa-miR-20a-5p	KIF3B	9371	NM_004798	93.6
hsa-miR-20a-5p	PTPN3	5774	NM_001145368	93.5
hsa-miR-20a-5p	NRIP3	56675	NM_020645	93.4
hsa-miR-20a-5p	ARHGAP26	23092	NM_001135608	93.3
hsa-miR-20a-5p	CALD1	800	NM_004342	93.3
hsa-miR-20a-5p	TAOK3	51347	NM_016281	93.3

hsa-miR-20a-5p	DRD1	1812	NM_000794	93.3
hsa-miR-20a-5p	NEDD4L	23327	NM_001144964	93.3
hsa-miR-20a-5p	SLITRK3	22865	NM_001318810	93.3
hsa-miR-20a-5p	BNC2	54796	NM_001317939	93.3
hsa-miR-20a-5p	BEST3	144453	NM_001282614	93.2
hsa-miR-20a-5p	ANO6	196527	NM_001025356	93.2
hsa-miR-20a-5p	HSPA8	3312	NM_153201	93.1
hsa-miR-20a-5p	SLAIN2	57606	NM_020846	93.1
hsa-miR-20a-5p	CCNG2	901	NM_004354	93.1
hsa-miR-20a-5p	ATL3	25923	NM_001290048	93.0
hsa-miR-20a-5p	FAM189A1	23359	NM_015307	93.0
hsa-miR-20a-5p	ERAP1	51752	NM_016442	93.0
hsa-miR-20a-5p	SUSD6	9766	NM_014734	92.9
hsa-miR-20a-5p	DENND5B	160518	NM_001308339	92.8
hsa-miR-20a-5p	CD69	969	NM_001781	92.6
hsa-miR-20a-5p	STRIP2	57464	NM_020704	92.6
hsa-miR-20a-5p	PRDM6	93166	NM_001136239	92.6
hsa-miR-20a-5p	BMPR2	659	NM_001204	92.6
hsa-miR-20a-5p	ELK3	2004	NM_001303511	92.5
hsa-miR-20a-5p	RUNX3	864	NM_001031680	92.5
hsa-miR-20a-5p	CEP120	153241	NM_001166226	92.4
hsa-miR-20a-5p	SIKE1	80143	NM_001102396	92.4
hsa-miR-20a-5p	RAB11FIP1	80223	NM_025151	92.4
hsa-miR-20a-5p	LRCH1	23143	NM_001164213	92.4
hsa-miR-20a-5p	URI1	8725	NM_003796	92.4
hsa-miR-20a-5p	IGSF10	285313	NM_001178145	92.3
hsa-miR-20a-5p	CENPQ	55166	NM_018132	92.3
hsa-miR-20a-5p	EGR2	1959	NM_000399	92.3
hsa-miR-20a-5p	SRCIN1	80725	NM_025248	92.3
hsa-miR-20a-5p	TRPV6	55503	NM_018646	92.2
hsa-miR-20a-5p	OSM	5008	NM_001319108	92.2
hsa-miR-20a-5p	TMEM168	64418	NM_001287497	92.2
hsa-miR-20a-5p	L3MBTL3	84456	NM_001007102	92.1
hsa-miR-20a-5p	HPS5	11234	NM_007216	92.1
hsa-miR-20a-5p	RGMB	285704	NM_001012761	92.1
hsa-miR-20a-5p	NTN4	59277	NM_021229	92.1
hsa-miR-20a-5p	LRP8	7804	NM_001018054	92.0
hsa-miR-20a-5p	OCRL	4952	NM_001318784	92.0
hsa-miR-20a-5p	TNKS2	80351	NM_025235	91.9
hsa-miR-20a-5p	FAM102A	399665	NM_001035254	91.9
hsa-miR-20a-5p	TGM2	7052	NM_198951	91.9
hsa-miR-20a-5p	UNC80	285175	NM_032504	91.8
hsa-miR-20a-5p	C14orf28	122525	NM_001017923	91.8
hsa-miR-20a-5p	LASP1	3927	NM_001271608	91.8
hsa-miR-20a-5p	ACSL4	2182	NM_001318509	91.8
hsa-miR-20a-5p	SESN3	143686	NM_001271594	91.8
hsa-miR-20a-5p	ETV1	2115	NM_001163147	91.8
hsa-miR-20a-5p	CTSK	1513	NM_000396	91.7
hsa-miR-20a-5p	RACGAP1	29127	NM_001126103	91.7
hsa-miR-20a-5p	HECTD2	143279	NM_001284274	91.7

hsa-miR-20a-5p	AP2B1	163	NM_001030006	91.7
hsa-miR-20a-5p	TMEM64	169200	NM_001008495	91.7
hsa-miR-20a-5p	ISM2	145501	NM_182509	91.7
hsa-miR-20a-5p	DNAJC16	23341	NM_001287811	91.6
hsa-miR-20a-5p	MAPRE3	22924	NM_001303050	91.5
hsa-miR-20a-5p	KLF11	8462	NM_001177716	91.5
hsa-miR-20a-5p	MAP10	54627	NM_019090	91.5
hsa-miR-20a-5p	LDLR	3949	NM_000527	91.4
hsa-miR-20a-5p	LYST	1130	NM_000081	91.4
hsa-miR-20a-5p	USP28	57646	NM_001301029	91.4
hsa-miR-20a-5p	TIAM1	7074	NM_003253	91.3
hsa-miR-20a-5p	PRR15	222171	NM_175887	91.2
hsa-miR-20a-5p	APCDD1	147495	NM_153000	91.2
hsa-miR-20a-5p	CC2D1A	54862	NM_017721	91.2
hsa-miR-20a-5p	ZFAND4	93550	NM_001128324	91.2
hsa-miR-20a-5p	SMOC1	64093	NM_001034852	91.1
hsa-miR-20a-5p	LRRC55	219527	NM_001005210	91.1
hsa-miR-20a-5p	TRIM3	10612	NM_001248006	91.1
hsa-miR-20a-5p	GNB5	10681	NM_006578	91.1
hsa-miR-20a-5p	CERCAM	51148	NM_001286760	91.0
hsa-miR-20a-5p	UBE3C	9690	NM_014671	91.0
hsa-miR-20a-5p	PCDHAC1	56135	NM_018898	91.0
hsa-miR-20a-5p	PCDHAC2	56134	NM_018899	91.0
hsa-miR-20a-5p	PCDHA1	56147	NM_018900	91.0
hsa-miR-20a-5p	PCDHA10	56139	NM_018901	91.0
hsa-miR-20a-5p	PCDHA11	56138	NM_018902	91.0
hsa-miR-20a-5p	PCDHA12	56137	NM_018903	91.0
hsa-miR-20a-5p	PCDHA13	56136	NM_018904	91.0
hsa-miR-20a-5p	PCDHA2	56146	NM_018905	91.0
hsa-miR-20a-5p	PCDHA3	56145	NM_018906	91.0
hsa-miR-20a-5p	PCDHA4	56144	NM_018907	91.0
hsa-miR-20a-5p	PCDHA5	56143	NM_018908	91.0
hsa-miR-20a-5p	PCDHA6	56142	NM_018909	91.0
hsa-miR-20a-5p	PCDHA7	56141	NM_018910	91.0
hsa-miR-20a-5p	PCDHA8	56140	NM_018911	91.0
hsa-miR-20a-5p	DAB2	1601	NM_001244871	91.0
hsa-miR-20a-5p	CCDC71L	168455	NM_175884	91.0
hsa-miR-20a-5p	DERL2	51009	NM_001304779	90.9
hsa-miR-20a-5p	SLC16A9	220963	NM_194298	90.9
hsa-miR-20a-5p	ARHGAP1	392	NM_004308	90.9
hsa-miR-20a-5p	ANKFY1	51479	NM_001257999	90.9
hsa-miR-20a-5p	IL6ST	3572	NM_175767	90.9
hsa-miR-20a-5p	ERC1	23085	NM_001301248	90.8
hsa-miR-20a-5p	RPS6KA4	8986	NM_001006944	90.8
hsa-miR-20a-5p	KIAA1191	57179	NM_001079684	90.8
hsa-miR-20a-5p	CAPRIN2	65981	NM_001002259	90.7
hsa-miR-20a-5p	NBEA	26960	NM_015678	90.7
hsa-miR-20a-5p	RAB8B	51762	NM_016530	90.7
hsa-miR-20a-5p	ZBTB33	10009	NM_001184742	90.7
hsa-miR-20a-5p	THRA	7067	NM_199334	90.6

hsa-miR-20a-5p	STK38	11329	NM_001305102	90.6
hsa-miR-20a-5p	IQSEC2	23096	NM_001111125	90.6
hsa-miR-20a-5p	KLHL15	80311	NM_030624	90.4
hsa-miR-20a-5p	AMER2	219287	NM_152704	90.4
hsa-miR-20a-5p	P2RX4	5025	NM_001256796	90.4
hsa-miR-20a-5p	ENTPD4	9583	NM_001128930	90.4
hsa-miR-20a-5p	SEMA4B	10509	NM_001324029	90.3
hsa-miR-20a-5p	S1PR1	1901	NM_001320730	90.3
hsa-miR-20a-5p	ARHGEF11	9826	NM_014784	90.2
hsa-miR-20a-5p	ATP12A	479	NM_001185085	90.2
hsa-miR-20a-5p	AFG1L	246269	NM_001323005	90.1
hsa-miR-20a-5p	PLXDC2	84898	NM_001282736	90.1
hsa-miR-20a-5p	SSX2IP	117178	NM_001166293	90.1
hsa-miR-20a-5p	FBXO31	79791	NM_001282683	90.1
hsa-miR-20a-5p	KIF23	9493	NM_001281301	90.1
hsa-miR-20a-5p	PXK	54899	NM_001289095	90.0
hsa-miR-340-3p	TULP4	56995	NM_001007466	98.1
hsa-miR-340-3p	LRTOMT	220074	NM_001205138	97.2
hsa-miR-340-3p	TAPBP	6892	NM_003190	95.9
hsa-miR-340-3p	C15orf40	123207	NM_001160113	94.7
hsa-miR-340-3p	INMT	11185	NM_001199219	93.3
hsa-miR-340-3p	TLCD2	727910	NM_001164407	92.9
hsa-miR-340-3p	CYB5R3	1727	NM_000398	91.0
hsa-miR-340-3p	HUS1	3364	NM_004507	90.8
hsa-miR-340-3p	ZNF850	342892	NM_001193552	90.3
hsa-miR-30d-5p	CELSR3	1951	NM_001407	100.0
hsa-miR-30d-5p	WDR7	23335	NM_015285	100.0
hsa-miR-30d-5p	STOX2	56977	NM_020225	99.9
hsa-miR-30d-5p	ANKRA2	57763	NM_023039	99.8
hsa-miR-30d-5p	MIER3	166968	NM_001297598	99.8
hsa-miR-30d-5p	KLHL20	27252	NM_014458	99.8
hsa-miR-30d-5p	PPARGC1B	133522	NM_001172699	99.8
hsa-miR-30d-5p	MKRN3	7681	NM_005664	99.7
hsa-miR-30d-5p	DCUN1D3	123879	NM_173475	99.7
hsa-miR-30d-5p	SCN2A	6326	NM_001040142	99.7
hsa-miR-30d-5p	NFAT5	10725	NM_001113178	99.7
hsa-miR-30d-5p	LIN28B	389421	NM_001004317	99.7
hsa-miR-30d-5p	BRWD3	254065	NM_153252	99.7
hsa-miR-30d-5p	TWF1	5756	NM_001242397	99.7
hsa-miR-30d-5p	PTGFRN	5738	NM_020440	99.6
hsa-miR-30d-5p	B3GNT5	84002	NM_032047	99.6
hsa-miR-30d-5p	FZD3	7976	NM_017412	99.6
hsa-miR-30d-5p	EED	8726	NM_001308007	99.5
hsa-miR-30d-5p	KLHL28	54813	NM_001308112	99.5
hsa-miR-30d-5p	XPO1	7514	NM_003400	99.4
hsa-miR-30d-5p	LRRC17	10234	NM_001031692	99.3
hsa-miR-30d-5p	BRWD1	54014	NM_033656	99.3
hsa-miR-30d-5p	PDE7A	5150	NM_001242318	99.3
hsa-miR-30d-5p	LHX8	431707	NM_001001933	99.2
hsa-miR-30d-5p	PRDM1	639	NM_001198	99.2

hsa-miR-30d-5p	POLR3E	55718	NM_001258033	99.2
hsa-miR-30d-5p	C9orf72	203228	NM_001256054	99.2
hsa-miR-30d-5p	NT5E	4907	NM_001204813	99.2
hsa-miR-30d-5p	TNRC6A	27327	NM_014494	99.2
hsa-miR-30d-5p	USP37	57695	NM_020935	99.1
hsa-miR-30d-5p	COL25A1	84570	NM_198721	99.1
hsa-miR-30d-5p	CCNE2	9134	NM_057749	99.1
hsa-miR-30d-5p	RFX6	222546	NM_173560	99.1
hsa-miR-30d-5p	SH3PXD2A	9644	NM_014631	99.1
hsa-miR-30d-5p	ALG10	84920	NM_032834	99.0
hsa-miR-30d-5p	PIP4K2A	5305	NM_005028	99.0
hsa-miR-30d-5p	RARG	5916	NM_000966	98.9
hsa-miR-30d-5p	MEIOB	254528	NM_001163560	98.8
hsa-miR-30d-5p	FNDC3A	22862	NM_001079673	98.8
hsa-miR-30d-5p	GMNC	647309	NM_001146686	98.7
hsa-miR-30d-5p	RFX7	64864	NM_022841	98.7
hsa-miR-30d-5p	ZBTB41	360023	NM_194314	98.6
hsa-miR-30d-5p	NCAM1	4684	NM_000615	98.5
hsa-miR-30d-5p	LCLAT1	253558	NM_001002257	98.5
hsa-miR-30d-5p	CCDC97	90324	NM_052848	98.5
hsa-miR-30d-5p	SNX16	64089	NM_022133	98.5
hsa-miR-30d-5p	TMEM181	57583	NM_020823	98.5
hsa-miR-30d-5p	PEX5L	51555	NM_001256750	98.5
hsa-miR-30d-5p	GABRB1	2560	NM_000812	98.5
hsa-miR-30d-5p	RIMBP2	23504	NM_015347	98.4
hsa-miR-30d-5p	PLAGL2	5326	NM_002657	98.4
hsa-miR-30d-5p	RTKN2	219790	NM_145307	98.3
hsa-miR-30d-5p	SCML1	6322	NM_001037535	98.3
hsa-miR-30d-5p	ANO4	121601	NM_001286616	98.3
hsa-miR-30d-5p	HCFC2	29915	NM_013320	98.3
hsa-miR-30d-5p	ROR1	4919	NM_005012	98.2
hsa-miR-30d-5p	ADAMTS9	56999	NM_001318781	98.2
hsa-miR-30d-5p	STXBP5	134957	NM_001127715	98.2
hsa-miR-30d-5p	LMBR1L	55716	NM_001300750	98.2
hsa-miR-30d-5p	CYP24A1	1591	NM_000782	98.2
hsa-miR-30d-5p	ZNRF1	84937	NM_032268	98.2
hsa-miR-30d-5p	NEDD4	4734	NM_001284338	98.1
hsa-miR-30d-5p	LIMCH1	22998	NM_001112717	98.1
hsa-miR-30d-5p	PLPPR4	9890	NM_001166252	98.1
hsa-miR-30d-5p	UBN2	254048	NM_173569	98.1
hsa-miR-30d-5p	PLPP6	403313	NM_203453	98.0
hsa-miR-30d-5p	MYH11	4629	NM_001040113	98.0
hsa-miR-30d-5p	ELL2	22936	NM_012081	97.9
hsa-miR-30d-5p	LMBR1	64327	NM_022458	97.9
hsa-miR-30d-5p	FRZB	2487	NM_001463	97.9
hsa-miR-30d-5p	MTDH	92140	NM_178812	97.9
hsa-miR-30d-5p	PTPN13	5783	NM_006264	97.8
hsa-miR-30d-5p	CHL1	10752	NM_001253387	97.8
hsa-miR-30d-5p	SCARA5	286133	NM_173833	97.7
hsa-miR-30d-5p	RGS8	85397	NM_001102450	97.7

hsa-miR-30d-5p	BDP1	55814	NM_018429	97.7
hsa-miR-30d-5p	UBE2J1	51465	NM_016021	97.7
hsa-miR-30d-5p	ADRA2A	150	NM_000681	97.6
hsa-miR-30d-5p	DESI2	51029	NM_001297746	97.5
hsa-miR-30d-5p	SCN3A	6328	NM_001081676	97.5
hsa-miR-30d-5p	SLC35A3	23443	NM_001271684	97.5
hsa-miR-30d-5p	NECAP1	25977	NM_015509	97.4
hsa-miR-30d-5p	TNIK	23043	NM_001161560	97.4
hsa-miR-30d-5p	REEP3	221035	NM_001001330	97.4
hsa-miR-30d-5p	PPP1R2	5504	NM_001291504	97.4
hsa-miR-30d-5p	E2F7	144455	NM_203394	97.3
hsa-miR-30d-5p	FAM160B1	57700	NM_020940	97.3
hsa-miR-30d-5p	YOD1	55432	NM_001276320	97.3
hsa-miR-30d-5p	EML1	2009	NM_001008707	97.3
hsa-miR-30d-5p	STIM2	57620	NM_001169117	97.3
hsa-miR-30d-5p	DCTN4	51164	NM_001135643	97.2
hsa-miR-30d-5p	NFIB	4781	NM_001190737	97.2
hsa-miR-30d-5p	STK35	140901	NM_080836	97.2
hsa-miR-30d-5p	VIM	7431	NM_003380	97.2
hsa-miR-30d-5p	CARF	79800	NM_001104586	97.2
hsa-miR-30d-5p	SETD5	55209	NM_001080517	97.2
hsa-miR-30d-5p	PTP4A1	7803	NM_003463	97.1
hsa-miR-30d-5p	PHTF2	57157	NM_001127357	97.0
hsa-miR-30d-5p	TENT2	167153	NM_001114393	97.0
hsa-miR-30d-5p	ATG12	9140	NM_001277783	97.0
hsa-miR-30d-5p	FAP	2191	NM_001291807	96.9
hsa-miR-30d-5p	ACTR3C	653857	NM_001164458	96.9
hsa-miR-30d-5p	SPEN	23013	NM_015001	96.9
hsa-miR-30d-5p	ITPK1	3705	NM_001142593	96.9
hsa-miR-30d-5p	YPEL2	388403	NM_001005404	96.9
hsa-miR-30d-5p	PPP3R1	5534	NM_000945	96.8
hsa-miR-30d-5p	STK39	27347	NM_013233	96.8
hsa-miR-30d-5p	TLL2	7093	NM_012465	96.8
hsa-miR-30d-5p	RUNX1	861	NM_001001890	96.7
hsa-miR-30d-5p	SOX9	6662	NM_000346	96.7
hsa-miR-30d-5p	YTHDF3	253943	NM_001277813	96.7
hsa-miR-30d-5p	ADAMTS3	9508	NM_014243	96.6
hsa-miR-30d-5p	DOLPP1	57171	NM_001135917	96.6
hsa-miR-30d-5p	SPOCK3	50859	NM_001040159	96.6
hsa-miR-30d-5p	FOXG1	2290	NM_005249	96.5
hsa-miR-30d-5p	CNOT9	9125	NM_001271634	96.5
hsa-miR-30d-5p	PLEKHM3	389072	NM_001080475	96.5
hsa-miR-30d-5p	SLC35C1	55343	NM_001145265	96.5
hsa-miR-30d-5p	ADAM19	8728	NM_033274	96.4
hsa-miR-30d-5p	RUNX2	860	NM_001015051	96.4
hsa-miR-30d-5p	KLF12	11278	NM_007249	96.4
hsa-miR-30d-5p	CCDC117	150275	NM_001284263	96.3
hsa-miR-30d-5p	EXTL2	2135	NM_001033025	96.3
hsa-miR-30d-5p	SEC24A	10802	NM_021982	96.2
hsa-miR-30d-5p	OTUD6B	51633	NM_001286745	96.2

hsa-miR-30d-5p	CCDC43	124808	NM_001099225	96.2
hsa-miR-30d-5p	PLEKHO2	80301	NM_001195059	96.1
hsa-miR-30d-5p	ZBTB11	27107	NM_014415	96.1
hsa-miR-30d-5p	SOCS1	8651	NM_003745	96.1
hsa-miR-30d-5p	ANKHD1	54882	NM_017747	96.1
hsa-miR-30d-5p	CHIC1	53344	NM_001039840	96.1
hsa-miR-30d-5p	ASB3	51130	NM_001201965	96.1
hsa-miR-30d-5p	TMEM170B	100113407	NM_001100829	96.0
hsa-miR-30d-5p	FBXO45	200933	NM_001105573	96.0
hsa-miR-30d-5p	S100PBP	64766	NM_001256121	96.0
hsa-miR-30d-5p	EML4	27436	NM_001145076	96.0
hsa-miR-30d-5p	SLC12A6	9990	NM_001042494	95.9
hsa-miR-30d-5p	PNKD	25953	NM_015488	95.8
hsa-miR-30d-5p	CNKSR2	22866	NM_001168647	95.8
hsa-miR-30d-5p	CHST2	9435	NM_004267	95.7
hsa-miR-30d-5p	ITGA6	3655	NM_000210	95.7
hsa-miR-30d-5p	SRSF7	6432	NM_001031684	95.7
hsa-miR-30d-5p	FAM43A	131583	NM_153690	95.7
hsa-miR-30d-5p	SNX18	112574	NM_001102575	95.6
hsa-miR-30d-5p	SH2B3	10019	NM_001291424	95.6
hsa-miR-30d-5p	XPR1	9213	NM_001135669	95.6
hsa-miR-30d-5p	DDAH1	23576	NM_001134445	95.6
hsa-miR-30d-5p	ZMYND8	23613	NM_001281771	95.5
hsa-miR-30d-5p	GOLGA1	2800	NM_002077	95.5
hsa-miR-30d-5p	PON2	5445	NM_000305	95.5
hsa-miR-30d-5p	PHIP	55023	NM_017934	95.5
hsa-miR-30d-5p	CCNT2	905	NM_001241	95.5
hsa-miR-30d-5p	MEOX2	4223	NM_005924	95.5
hsa-miR-30d-5p	RRAD	6236	NM_001128850	95.5
hsa-miR-30d-5p	SEC23A	10484	NM_006364	95.4
hsa-miR-30d-5p	STAC	6769	NM_001292049	95.4
hsa-miR-30d-5p	CALCR	799	NM_001164737	95.4
hsa-miR-30d-5p	PRUNE2	158471	NM_015225	95.4
hsa-miR-30d-5p	TP53INP1	94241	NM_001135733	95.4
hsa-miR-30d-5p	FKBP3	2287	NM_002013	95.1
hsa-miR-30d-5p	ANKRD17	26057	NM_001286771	95.1
hsa-miR-30d-5p	TLCD4	148534	NM_001199679	95.1
hsa-miR-30d-5p	NEURL1B	54492	NM_001142651	95.1
hsa-miR-30d-5p	UBE2V2	7336	NM_003350	95.0
hsa-miR-30d-5p	BRD1	23774	NM_001304808	95.0
hsa-miR-30d-5p	RAP2C	57826	NM_001271186	95.0
hsa-miR-30d-5p	SEC22C	9117	NM_032970	95.0
hsa-miR-30d-5p	PCDH17	27253	NM_001040429	95.0
hsa-miR-30d-5p	ZCCHC2	54877	NM_017742	94.9
hsa-miR-30d-5p	SOCS3	9021	NM_003955	94.9
hsa-miR-30d-5p	MLXIP	22877	NM_014938	94.9
hsa-miR-30d-5p	WDR82	80335	NM_025222	94.9
hsa-miR-30d-5p	SMAD1	4086	NM_001003688	94.8
hsa-miR-30d-5p	DLG5	9231	NM_004747	94.8
hsa-miR-30d-5p	TNRC6B	23112	NM_001024843	94.7

hsa-miR-30d-5p	KIAA0408	9729	NM_014702	94.7
hsa-miR-30d-5p	RASA2	5922	NM_001303245	94.7
hsa-miR-30d-5p	SIX1	6495	NM_005982	94.7
hsa-miR-30d-5p	TBC1D10B	26000	NM_015527	94.7
hsa-miR-30d-5p	MARCHF6	10299	NM_001270660	94.6
hsa-miR-30d-5p	LPGAT1	9926	NM_001320808	94.6
hsa-miR-30d-5p	FRMPD1	22844	NM_014907	94.6
hsa-miR-30d-5p	COL13A1	1305	NM_001130103	94.6
hsa-miR-30d-5p	A1CF	29974	NM_001198818	94.5
hsa-miR-30d-5p	DSG2	1829	NM_001943	94.4
hsa-miR-30d-5p	OTUD4	54726	NM_001102653	94.4
hsa-miR-30d-5p	SACS	26278	NM_001278055	94.4
hsa-miR-30d-5p	LRRC8C	84230	NM_032270	94.4
hsa-miR-30d-5p	RAB8A	4218	NM_005370	94.3
hsa-miR-30d-5p	MAST4	375449	NM_001164664	94.3
hsa-miR-30d-5p	CD2AP	23607	NM_012120	94.3
hsa-miR-30d-5p	OSBPL8	114882	NM_001003712	94.3
hsa-miR-30d-5p	IL1RAPL2	26280	NM_017416	94.3
hsa-miR-30d-5p	MZT1	440145	NM_001071775	94.2
hsa-miR-30d-5p	RAB32	10981	NM_006834	94.1
hsa-miR-30d-5p	TEPSIN	146705	NM_144679	94.1
hsa-miR-30d-5p	VAT1L	57687	NM_020927	94.1
hsa-miR-30d-5p	BNIP3L	665	NM_004331	94.1
hsa-miR-30d-5p	R3HDM1	23518	NM_001282798	94.0
hsa-miR-30d-5p	TMOD2	29767	NM_001142885	94.0
hsa-miR-30d-5p	PAWR	5074	NM_002583	94.0
hsa-miR-30d-5p	KMT2C	58508	NM_170606	94.0
hsa-miR-30d-5p	ZNF608	57507	NM_020747	94.0
hsa-miR-30d-5p	NA	NA	NM_001017973	94.0
hsa-miR-30d-5p	P4HA2	8974	NM_001017974	94.0
hsa-miR-30d-5p	SNX33	257364	NM_001318146	94.0
hsa-miR-30d-5p	FNIP2	57600	NM_001323916	94.0
hsa-miR-30d-5p	CCNK	8812	NM_001099402	94.0
hsa-miR-30d-5p	ERLIN1	10613	NM_001100626	94.0
hsa-miR-30d-5p	ZPBP2	124626	NM_198844	93.9
hsa-miR-30d-5p	PAPOLA	10914	NM_001252006	93.9
hsa-miR-30d-5p	PSMD7	5713	NM_002811	93.9
hsa-miR-30d-5p	LGI1	9211	NM_001308275	93.9
hsa-miR-30d-5p	RAPGEF4	11069	NM_001100397	93.9
hsa-miR-30d-5p	MAB21L1	4081	NM_005584	93.8
hsa-miR-30d-5p	ADAMTS6	11174	NM_197941	93.8
hsa-miR-30d-5p	SHISA3	152573	NM_001080505	93.8
hsa-miR-30d-5p	PGM1	5236	NM_001172818	93.8
hsa-miR-30d-5p	RAP1B	5908	NM_001010942	93.8
hsa-miR-30d-5p	LPP	4026	NM_001167671	93.8
hsa-miR-30d-5p	PRLR	5618	NM_000949	93.7
hsa-miR-30d-5p	OXR1	55074	NM_001198532	93.7
hsa-miR-30d-5p	HNRNPUL2	221092	NM_001079559	93.7
hsa-miR-30d-5p	SEMA6B	10501	NM_032108	93.7
hsa-miR-30d-5p	KCTD8	386617	NM_198353	93.6

hsa-miR-30d-5p	LARGE1	9215	NM_004737	93.6
hsa-miR-30d-5p	SCN1A	6323	NM_001165963	93.6
hsa-miR-30d-5p	FLVCR2	55640	NM_001195283	93.6
hsa-miR-30d-5p	MYO1H	283446	NM_001101421	93.6
hsa-miR-30d-5p	MAP6	4135	NM_207577	93.6
hsa-miR-30d-5p	FRMD6	122786	NM_001042481	93.6
hsa-miR-30d-5p	SCAF4	57466	NM_001145444	93.5
hsa-miR-30d-5p	ZBTB44	29068	NM_014155	93.5
hsa-miR-30d-5p	CPNE8	144402	NM_153634	93.5
hsa-miR-30d-5p	FAM91A1	157769	NM_001317917	93.5
hsa-miR-30d-5p	NADK	65220	NM_001198993	93.3
hsa-miR-30d-5p	FBXO32	114907	NM_001242463	93.3
hsa-miR-30d-5p	SPAST	6683	NM_014946	93.2
hsa-miR-30d-5p	ME1	4199	NM_002395	93.2
hsa-miR-30d-5p	NTNG1	22854	NM_001113226	93.1
hsa-miR-30d-5p	LRRC40	55631	NM_017768	93.1
hsa-miR-30d-5p	SLC38A7	55238	NM_018231	93.1
hsa-miR-30d-5p	RAPH1	65059	NM_213589	93.1
hsa-miR-30d-5p	MAP3K21	84451	NM_032435	93.1
hsa-miR-30d-5p	PPARGC1A	10891	NM_013261	93.1
hsa-miR-30d-5p	FAM210B	116151	NM_080821	93.1
hsa-miR-30d-5p	TMEM229A	730130	NM_001136002	93.1
hsa-miR-30d-5p	LRRK2	120892	NM_198578	93.0
hsa-miR-30d-5p	WASHC4	23325	NM_001293640	93.0
hsa-miR-30d-5p	VPS26B	112936	NM_052875	93.0
hsa-miR-30d-5p	DOK5	55816	NM_018431	93.0
hsa-miR-30d-5p	PALM2AKAP2	445815	NM_001037293	93.0
hsa-miR-30d-5p	RASA1	5921	NM_002890	93.0
hsa-miR-30d-5p	TTLL7	79739	NM_024686	93.0
hsa-miR-30d-5p	YPEL5	51646	NM_001127399	93.0
hsa-miR-30d-5p	MYBL2	4605	NM_001278610	92.9
hsa-miR-30d-5p	MAN1A2	10905	NM_006699	92.9
hsa-miR-30d-5p	PFN2	5217	NM_002628	92.9
hsa-miR-30d-5p	LYPLAL1	127018	NM_001300769	92.9
hsa-miR-30d-5p	MCF2L	23263	NM_001112732	92.9
hsa-miR-30d-5p	CAND1	55832	NM_018448	92.9
hsa-miR-30d-5p	PIK3CD	5293	NM_005026	92.9
hsa-miR-30d-5p	DLL4	54567	NM_019074	92.9
hsa-miR-30d-5p	LOC283710	283710	NM_001243538	92.8
hsa-miR-30d-5p	PER2	8864	NM_022817	92.8
hsa-miR-30d-5p	UGT2A3	79799	NM_024743	92.8
hsa-miR-30d-5p	SIX4	51804	NM_017420	92.7
hsa-miR-30d-5p	UBN1	29855	NM_001079514	92.7
hsa-miR-30d-5p	AFAP1L2	84632	NM_001001936	92.7
hsa-miR-30d-5p	PTPDC1	138639	NM_001253829	92.7
hsa-miR-30d-5p	STX2	2054	NM_001980	92.7
hsa-miR-30d-5p	MBNL3	55796	NM_133486	92.7
hsa-miR-30d-5p	MAST3	23031	NM_015016	92.7
hsa-miR-30d-5p	BCL11B	64919	NM_001282237	92.6
hsa-miR-30d-5p	RUNDC3B	154661	NM_001134405	92.6

hsa-miR-30d-5p	FAM110B	90362	NM_147189	92.6
hsa-miR-30d-5p	GATM	2628	NM_001321015	92.6
hsa-miR-30d-5p	COL9A3	1299	NM_001853	92.6
hsa-miR-30d-5p	TMEM87B	84910	NM_032824	92.6
hsa-miR-30d-5p	RARRES1	5918	NM_206963	92.5
hsa-miR-30d-5p	TRIM13	10206	NM_001007278	92.5
hsa-miR-30d-5p	C4orf19	55286	NM_001104629	92.5
hsa-miR-30d-5p	MEX3B	84206	NM_032246	92.4
hsa-miR-30d-5p	RBM12	10137	NM_001198838	92.4
hsa-miR-30d-5p	GLDC	2731	NM_000170	92.3
hsa-miR-30d-5p	LRRC8D	55144	NM_001134479	92.2
hsa-miR-30d-5p	ACTC1	70	NM_005159	92.2
hsa-miR-30d-5p	ACTR1A	10121	NM_005736	92.2
hsa-miR-30d-5p	RNF165	494470	NM_001256758	92.2
hsa-miR-30d-5p	JPH4	84502	NM_001146028	92.1
hsa-miR-30d-5p	CPSF6	11052	NM_001300947	92.1
hsa-miR-30d-5p	CBLB	868	NM_001321790	92.1
hsa-miR-30d-5p	SETD7	80854	NM_030648	92.1
hsa-miR-30d-5p	MAP3K13	9175	NM_001242314	92.1
hsa-miR-30d-5p	ZNF280B	140883	NM_080764	92.1
hsa-miR-30d-5p	CEP41	95681	NM_001257158	92.1
hsa-miR-30d-5p	YY2	404281	NM_206923	92.1
hsa-miR-30d-5p	RAB38	23682	NM_022337	92.1
hsa-miR-30d-5p	NRIP1	8204	NM_003489	92.1
hsa-miR-30d-5p	SLC35F1	222553	NM_001029858	92.0
hsa-miR-30d-5p	ZFY	7544	NM_001145275	92.0
hsa-miR-30d-5p	UNC5C	8633	NM_003728	92.0
hsa-miR-30d-5p	TENM3	55714	NM_001080477	92.0
hsa-miR-30d-5p	CALU	813	NM_001130674	91.9
hsa-miR-30d-5p	MYO5A	4644	NM_000259	91.9
hsa-miR-30d-5p	LRFN2	57497	NM_020737	91.8
hsa-miR-30d-5p	ARHGEF6	9459	NM_001306177	91.8
hsa-miR-30d-5p	STX16	8675	NM_001001433	91.8
hsa-miR-30d-5p	FAM133A	286499	NM_001171109	91.7
hsa-miR-30d-5p	CAMKK2	10645	NM_172226	91.7
hsa-miR-30d-5p	BAHD1	22893	NM_001301132	91.7
hsa-miR-30d-5p	DNMT3A	1788	NM_001320893	91.7
hsa-miR-30d-5p	PRKAA2	5563	NM_006252	91.6
hsa-miR-30d-5p	ZNF518A	9849	NM_001278524	91.6
hsa-miR-30d-5p	FAM126B	285172	NM_173822	91.6
hsa-miR-30d-5p	TTBK1	84630	NM_032538	91.6
hsa-miR-30d-5p	EDNRA	1909	NM_001166055	91.6
hsa-miR-30d-5p	BNC1	646	NM_001301206	91.6
hsa-miR-30d-5p	CBX2	84733	NM_005189	91.6
hsa-miR-30d-5p	PHF13	148479	NM_153812	91.6
hsa-miR-30d-5p	PDSS1	23590	NM_001321978	91.5
hsa-miR-30d-5p	PAAF1	80227	NM_001267803	91.5
hsa-miR-30d-5p	SEPTIN7	989	NM_001011553	91.5
hsa-miR-30d-5p	ARID4A	5926	NM_002892	91.5
hsa-miR-30d-5p	APBA1	320	NM_001163	91.4

hsa-miR-30d-5p	ABL1	25	NM_005157	91.4
hsa-miR-30d-5p	CLOCK	9575	NM_004898	91.4
hsa-miR-30d-5p	PAXBP1	94104	NM_016631	91.4
hsa-miR-30d-5p	ASB4	51666	NM_016116	91.4
hsa-miR-30d-5p	MBTPS2	51360	NM_015884	91.3
hsa-miR-30d-5p	RAD23B	5887	NM_001244713	91.3
hsa-miR-30d-5p	MMD	23531	NM_012329	91.3
hsa-miR-30d-5p	SKP2	6502	NM_001243120	91.2
hsa-miR-30d-5p	RHOB	388	NM_004040	91.2
hsa-miR-30d-5p	IP6K3	117283	NM_001142883	91.2
hsa-miR-30d-5p	CACHD1	57685	NM_001293274	91.2
hsa-miR-30d-5p	RAB23	51715	NM_001278666	91.2
hsa-miR-30d-5p	EPG5	57724	NM_020964	91.2
hsa-miR-30d-5p	CAPZA1	829	NM_006135	91.2
hsa-miR-30d-5p	CDCA7	83879	NM_031942	91.2
hsa-miR-30d-5p	NDEL1	81565	NM_001025579	91.1
hsa-miR-30d-5p	VAT1	10493	NM_006373	91.1
hsa-miR-30d-5p	DLGAP1	9229	NM_001242761	91.1
hsa-miR-30d-5p	MAP11	55262	NM_001303470	91.1
hsa-miR-30d-5p	ZNF521	25925	NM_001308225	91.0
hsa-miR-30d-5p	GALNT1	2589	NM_020474	91.0
hsa-miR-30d-5p	GRM3	2913	NM_000840	90.9
hsa-miR-30d-5p	CAMK2D	817	NM_001221	90.9
hsa-miR-30d-5p	CSNK1A1	1452	NM_001025105	90.9
hsa-miR-30d-5p	RAI14	26064	NM_001145520	90.9
hsa-miR-30d-5p	SYPL1	6856	NM_006754	90.8
hsa-miR-30d-5p	MFSD6	54842	NM_017694	90.8
hsa-miR-30d-5p	LOX	4015	NM_001178102	90.8
hsa-miR-30d-5p	GNA13	10672	NM_001282425	90.8
hsa-miR-30d-5p	HDAC9	9734	NM_001204144	90.7
hsa-miR-30d-5p	PPP1R1C	151242	NM_001261424	90.7
hsa-miR-30d-5p	FAM83F	113828	NM_138435	90.7
hsa-miR-30d-5p	TOGARAM1	23116	NM_015091	90.7
hsa-miR-30d-5p	TSPAN2	10100	NM_001308315	90.7
hsa-miR-30d-5p	PDE4D	5144	NM_001104631	90.6
hsa-miR-30d-5p	OSTM1	28962	NM_014028	90.6
hsa-miR-30d-5p	FBXL20	84961	NM_001184906	90.6
hsa-miR-30d-5p	SLC7A10	56301	NM_019849	90.5
hsa-miR-30d-5p	SSH2	85464	NM_001282129	90.5
hsa-miR-30d-5p	GFPT2	9945	NM_005110	90.4
hsa-miR-30d-5p	AGO3	192669	NM_024852	90.4
hsa-miR-30d-5p	ELAVL2	1993	NM_001171195	90.4
hsa-miR-30d-5p	SLC25A36	55186	NM_001104647	90.4
hsa-miR-30d-5p	PPP3CA	5530	NM_000944	90.4
hsa-miR-30d-5p	MFHAS1	9258	NM_004225	90.4
hsa-miR-30d-5p	FOXD1	2297	NM_004472	90.4
hsa-miR-30d-5p	TFDP1	7027	NM_007111	90.4
hsa-miR-30d-5p	NUP93	9688	NM_001242795	90.3
hsa-miR-30d-5p	KDM3A	55818	NM_001146688	90.3
hsa-miR-30d-5p	NAA25	80018	NM_024953	90.3

hsa-miR-30d-5p	FLVCR1	28982	NM_014053	90.3
hsa-miR-30d-5p	HOXA1	3198	NM_153620	90.3
hsa-miR-30d-5p	SAMD4A	23034	NM_001161576	90.3
hsa-miR-30d-5p	MROH9	80133	NM_025063	90.3
hsa-miR-30d-5p	ADAM9	8754	NM_003816	90.3
hsa-miR-30d-5p	NR6A1	2649	NM_001278546	90.3
hsa-miR-30d-5p	CMTM4	146223	NM_181521	90.3
hsa-miR-30d-5p	MSI2	124540	NM_001322250	90.2
hsa-miR-30d-5p	OVOL1	5017	NM_004561	90.2
hsa-miR-30d-5p	PPP1R12A	4659	NM_001143885	90.2
hsa-miR-30d-5p	GNAI2	2771	NM_001166425	90.2
hsa-miR-30d-5p	TENM1	10178	NM_001163278	90.2
hsa-miR-30d-5p	NAALADL2	254827	NM_207015	90.2
hsa-miR-30d-5p	EDEM3	80267	NM_001319960	90.1
hsa-miR-30d-5p	GJA1	2697	NM_000165	90.0
hsa-miR-30d-5p	RHEBL1	121268	NM_001303126	90.0
hsa-miR-30d-5p	EXOC6	54536	NM_001013848	90.0
hsa-miR-30d-5p	LMLN	89782	NM_001136049	90.0
hsa-miR-30d-5p	RASD1	51655	NM_001199989	90.0
